# Measuring destination image dissonance: A compositional data analysis of cross-media representations in Sanya’s Tourism

**DOI:** 10.1371/journal.pone.0360383

**Published:** 2026-10-15

**Authors:** Xueli Li, Jianlan Ding, Yina Zhang, Tao Hu

**Affiliations:** 1 Xi’an Physical Education University, School of Sports Economics and Management, Xi’an, China; 2 Hainan University, School of International Tourism and Public Administration, Haikou, China; IESEG School of Management, FRANCE

## Abstract

This study examines Sanya’s tourism image disparities across cognitive, spatial, and affective dimensions in Press Generated Content (PGC), Tourist Generated Content (TGC), and Official Generated Content (OGC) (2013–2022), comparing pre-pandemic (2013–2019) and COVID-19 (2020–2022) periods. Results show: cognitive consistency in “tourism/hotels” but TGC focusing on “coastal + food” vs. PGC/OGC emphasizing policy elements; spatial misalignment between policy-driven spaces and tourists’ preferred spaces, revealing a systematic dissonance between institutional promotion and on-the-ground practices; affective dominance of “enjoyment/romance” but TGC expressing unmet needs and OGC suppressing negatives. Systematic cognitive divergence exists between official and tourist narratives. The Aitchison distance measuring this divergence jumps from 19.574 in the pre-pandemic phase (2013–2019) to 40.648 during the pandemic-impacted phase (2020–2022), demonstrating that structural gaps between the two narrative systems become more pronounced amid the public health crisis. Targeted strategies are accordingly proposed to narrow such narrative discrepancies.

## 1. Introduction

Since the concept of Tourist Destination Image (TDI) emerged, it has become a core element for understanding tourist behavior and destination competitiveness. A central proposition in this field is that the media plays a crucial role in the construction and dissemination of tourist destination images [1, 2]. Beyond the mere presentation of images, the consistency between a destination’s “projected image” and tourists’ “perceived image” is regarded as the foundation for building tourist trust and achieving tourist satisfaction [3,4]. Multiple studies consistently indicate that image inconsistency may lead to the disappointment of tourists’ expectations, ultimately reducing their satisfaction and loyalty [5,6]. Therefore, promoting the consistency of destination images conveyed by different media sources has become a key objective for destination marketers [7,8].

The digital age has given rise to a fragmented media landscape, spawning multiple parallel image-generating entities: these include Press Generated Content (PGC) such as news reports, Official Generated Content (OGC) released by destination marketing organizations, and Tourist Generated Content (TGC) in the form of reviews and social media posts [9,10]. While this diversity provides a wealth of data, it also complicates the management of a unified destination image, given that these information sources often adhere to different objectives and convey divergent narrative content.

Traditionally, research has largely relied on the “cognitive-affective” dualistic model to deconstruct destination images [11]. However, tourism geographers have long advocated for the integration of the “spatial” dimension into research, arguing that destination images inherently possess a “place attribute” and involve the cognition of spatial relationships and spatial patterns [12]. In recent years, the popularity of “internet-famous check-in spots” and “City Walk” has further highlighted the crucial importance of the spatial dimension in the digital age [13]. Nevertheless, few studies have systematically integrated the cognitive, affective, and spatial dimensions into a unified framework for cross-media comparative analysis.

At the methodological level, evaluating the inconsistency among these multi-source images remains challenging. Most existing studies adopt content analysis or semantic network analysis. While these methods have certain effectiveness, they often struggle to quantitatively capture the relative and associative characteristics of image elements in different media compositions [3]. Compositional Data Analysis (CoDA) offers a reliable solution to this problem, as it is specifically designed to analyze data that “represents the various parts of a whole” [8]. However, its application in tourism image research, particularly in the measurement of cross-media inconsistency, is still in its nascent stage.

To address the aforementioned theoretical and methodological gaps, this study adopts the Compositional Data Analysis (CoDA) framework to explore the image inconsistency of Sanya (China) as a tourist destination from three dimensions: cognitive, spatial, and affective. By comparing three types of media content, namely Press Generated Content (PGC, i.e., news reports), Tourist Generated Content (TGC, i.e., tourist reviews), and Official Generated Content (OGC, i.e., official social media content), over a ten-year period (2013−2022, divided into two phases: pre-COVID-19 and during COVID-19), this study aims to achieve the following objectives:

(1) To quantitatively measure the degree of differences in cognitive, spatial, and affective images among PGC, TGC, and OGC;
(2) To compare the cross-media image divergence patterns observed in the pre-COVID period and the pandemic-affected period through descriptive temporal contrast;
(3) To put forward targeted recommendations for bridging the image gap based on the data findings.

## 2. Literature review

### 2.1. Core dimensions of tourism destination image

Since Mayo (1973) proposed the concept of Tourist Destination Image (TDI) [14], the core research agenda of this field has consistently revolved around how destinations are perceived. Early theoretical studies have clarified its multidimensional nature. Gunn (1972) divided destination image into organic image and induced image [15]. Echtner & Ritchie (1991) proposed a continuous dimensional framework encompassing two dimensions, namely “attribute-based vs. holistic perception” and “functional attributes vs. psychological attributes” [16]. Among numerous theoretical models, the cognitive-affective model has gradually emerged as the dominant paradigm [17,18]. This model posits that cognitive evaluation (beliefs about a destination’s attributes) precedes and influences affective responses (feelings toward the destination), with both jointly shaping the overall image.

However, the dominant paradigm has long overlooked the indispensable spatiality inherent in tourist experiences. Lynch’s (1960) pioneering research on urban image has long pointed out that spatial cognition and mental maps are central to how people comprehend and navigate the environment [12]. In the tourism context, space is far more than a static backdrop; it is an active constituent of the experience that influences itinerary organization, travel planning, and place memory. In recent years, the rise of internet-famous check-in spots and the popularity of City Walk have vividly confirmed the critical role of spatial elements in shaping destination images in the digital age [13,19,20]. Despite the significance of the spatial dimension, most existing studies isolate it from cognition and affect, failing to develop a systematic integrated analytical framework. This study constructs a “cognitive-spatial-affective” tripartite framework to more comprehensively capture the complex composition of destination images.

### 2.2. Multi-subject representation and cross-media image differences

The fragmentation of the digital media ecosystem has spawned diverse image-generating entities. Traditionally, destination narratives were dominated by Official Generated Content (OGC) and Press Generated Content (PGC); the rise of social media has established Tourist Generated Content (TGC) as a core force in co-constructing destination images, thus breaking the original centralized image-building model [9,21].

The diversification of representational subjects inevitably leads to image inconsistency. Numerous empirical studies have confirmed the discrepancy between projected images and perceived images. For instance, Stepchenkova & Zhan (2013) revealed systematic differences in visual representations between destination marketing organizations (DMOs) and tourists through image content analysis [22]; Wu et al. (2018) employed semantic network analysis to examine cognitive and affective misalignments between official websites and travel blogs [23]. In recent years, the research scope has been further expanded from simple OGC-TGC binary comparisons [24–26] to incorporating the resident perspective [27,28] and the perceptual heterogeneity of different tourist groups such as local residents, visited tourists and potential tourists [29–33]. Research media has also expanded from pure text to integrating visual and spatial analyses [34–36].

Despite the continuous deepening of research, a notable limitation remains as most studies only focus on comparisons between two types of subjects or two media channels and have not yet conducted simultaneous and systematic examination of the three core media types (PGC, OGC, TGC) within the integrated cognitive-spatial-affective framework.

### 2.3. The evolution of methodology and the introduction of compositional data analysis

Research methods for destination image have evolved alongside the advancement of data types and technologies. From traditional content analysis and semantic network analysis to the emerging topic modeling and machine learning in recent years, these methods have achieved remarkable results in identifying the content of image representations [3,37]. However, they struggle to effectively handle the compositional nature of image data, which means the characteristics of an image are defined by the relative importance (proportional structure) of each element rather than their absolute frequencies.

Raw keyword absolute frequencies are converted into proportional values; the resulting proportional datasets of image elements fall under compositional data subject to the constant sum constraint where the total sum of proportions within one medium equals 100 percent. Applying traditional statistical methods such as correlation analysis and Euclidean distance to such constrained proportional data can lead to spurious correlation [38].

Compositional Data Analysis (CoDA) provides a rigorous mathematical foundation for such relative value data. It maps compositional data from a simplex space to a real space through log-ratio transformation, thereby enabling the safe application of standard statistical methods. Its core indicator, the Aitchison distance, is specifically designed to measure the overall differences between compositional combinations. Although CoDA is regarded as a promising method for tourism analysis [8,39], its application to the specific issue of multidimensional and cross-media image difference measurement requires further exploration.

## 3. Methodology

Sanya is China’s only international tourist city with tropical coastal characteristics. Since the 1980s, Sanya’s tourism has always been oriented towards internationalization, rapidly gaining popularity through the strategy of “beauty and attention economy”. In recent years, Sanya has rapidly gained popularity across major networks by virtue of its online marketing strength, winning numerous accolades and ranking among China’s most influential urban tourism destinations. From 2021 to 2024, it has consistently ranked among the top 5 in China’s urban tourism influence rankings and first among coastal tourist destinations. The rising online buzz demonstrates Sanya’s high exposure in the digital media era, and the process and characteristics of digital media in its image construction are typical. Taking Sanya as a case study provides reference for the digital image construction and marketing promotion of other similar tourist destinations in the digital-intelligent era.

This study aims to reveal the representational dissonance of Sanya’s destination image by comparing the compositional differences across three types of media (PGC, TGC, OGC) in terms of cognitive, spatial and affective dimensions. To achieve this objective, this study adopts the systematic research workflow illustrated in the following figure, which encompasses the entire process from data collection and preprocessing to quantification and analysis (Fig 1).

**Fig 1:**
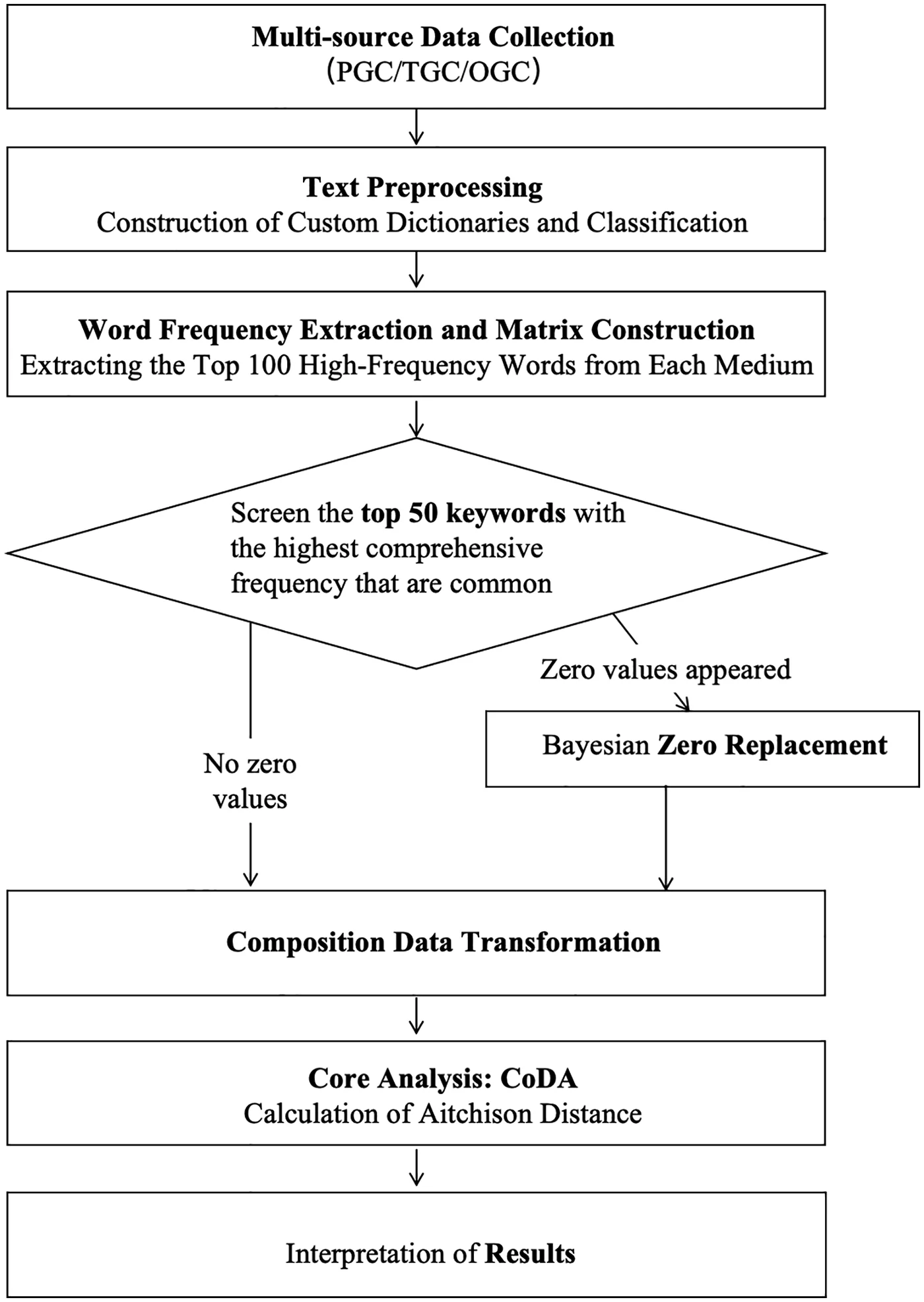
Data analysis process.

### 3.1. Data sources and collection

This study selected three types of media data from 2013 to 2022. Press Generated Content (PGC) was sourced from the WiseSearch Chinese News Database, representing the image portrayed by authoritative media reports; Tourist Generated Content (TGC) was obtained from travelogues on the Ctrip platform, representing the image perceived by tourists; Official Generated Content (OGC) was collected from the official WeChat public account of “*Sanya Tourism and Culture*”, representing the projected image of the destination management organization.

The three categories of data form mutually complementary discourse systems: PGC reflects mass media’s objective reporting perspective, OGC reflects official destination marketing positioning, and TGC reflects tourists’ real experiential feelings. Tripartite comparison avoids the one-sided bias of dual-source comparison in most existing studies.

To compare cross-media destination image patterns across distinct time contexts, the research period was divided into two phases. Phase 1 (2013–2019) was the normal development period before the pandemic, and Phase 2 (2020–2022) was the period of pandemic impact and recovery. The collection and analysis processes of the data used in this study—including key fields such as publication time, title, and full text, and covering 13,427 valid news reports, 5,221 travelogues, and 18,800 official articles—are fully compliant with the Terms of Service of WiseSearch, the User Agreement of the Ctrip Platform, and the Platform Rules of WeChat Official Accounts. These data are exclusively used for academic research purposes, with no unauthorized use, reproduction, or distribution conducted.

#### 3.1.1. Data of news reported image.

Despite the rapid development of new media, the importance of newspapers cannot be overshadowed. Compared with the low entry threshold of new media, news events and opinions published in newspapers exhibit greater authority [40]. Founded in 1998, WiseSearch, which is based on information processing technology from The Chinese University of Hong Kong, provides information services and owns a global Chinese news information database covering 470,000 information sources, holding absolute advantages in content breadth and media resource diversity [41]. As its core product, the WiseSearch news database includes over 1,200 print media resources, news information from more than 10,000 websites/forums/blogs, and 1,500 + social media platforms, enabling users to access news reports from a panoramic perspective.

Thus, news data about Sanya tourism, sourced from digital newspapers and covering the period 2000-01-01–2022-12-31, were collected from WiseSearch’s online newspapers and news apps (hereinafter referred to as “news reports”), considering Sanya’s tourism development timeline, news record characteristics, and data accessibility. A total of 13,427 news reports were obtained (excluding duplicates, foreign-language reports, and content from overseas media), covering 188 key newspapers (including central, local, and industry publications). The collected fields include publication time, newspaper name, news title, and content, totaling over 6.55 million words. Classified as Press Generated Content (PGC), this data source is hereinafter referred to as the “news-reported image”.

#### 3.1.2. Data of tourist-perceived image.

With the increasing trend of tourist interaction needs, multiple online travel application platforms have set up functional sections such as “travel reviews, comments, and travelogues”, featuring extensive Tourist Generated Content (TGC). Examples include Ctrip, Qunar, Mafengwo, Dianping, and Xiaohongshu. According to Alexa ranking queries, “Ctrip” ranks first among China’s online travel applications with the highest activity level. Founded in 1999, Ctrip.com was successfully listed on NASDAQ in 2003 and went public on the Hong Kong Stock Exchange as Ctrip Group in 2021. During this period, it has engaged in in-depth strategic collaborations with Tongcheng, Tuniu, e-Long, Qunar, and the renowned foreign online travel platform TripAdvisor through acquisitions, investments, and mergers.

Industry research data verify Ctrip’s dominant position among domestic OTA platforms. QuestMobile’s official 2025 Panoramic Ecological Traffic Semi-Annual Report confirms Ctrip maintains the largest unique user base and the highest volume of travel notes across all domestic travel applications [42]. Given its leading market penetration and massive tourist contributor pool, Ctrip travelogues can adequately reflect mainstream domestic tourists’ authentic destination perceptions.

The earliest travelogue about Sanya on Ctrip’s travelogue section was published in 2012. Thus, Sanya travelogues from January 1, 2012, to December 31, 2022, were collected, including four fields: publication time, travelogue title, content, and blogger. A total of 5,221 travelogues (19.44 million words) were obtained, hereinafter referred to as the “tourist-perceived image”.

#### 3.1.3. Data of official projected image.

Based on the query of weight and Alexa ranking for applications representing official projected images on Chinaz.com, the results show that “WeChat” ranks first in the activity list of China’s APP applications with an activity score of 23. The official WeChat public account “Sanya Tourism and Culture” (Sanya Tourism, Culture, Radio, Television and Sports Bureau’s interactive platform) is government-certified, publishing 3–6 daily articles related to Sanya tourism since 2013. Meanwhile, a research report by *Sojern* indicates that a significant proportion of marketing expenditure by tourism enterprises is allocated to WeChat channels, leading to the selection of WeChat public accounts as the official projection medium.

Thus, data from January 1, 2013, to December 31, 2022, were collected, including three fields: publication time, article title, and content. A total of 18,800 relevant articles (20.22 million words) were obtained. Classified as Official Generated Content (OGC), this data source is hereinafter referred to as the “official projected image”.

### 3.2. Text preprocessing and keyword extraction

To construct a high-quality dictionary that accurately reflects Sanya’s tourism image, we adopted the following systematic iterative process to ensure the reliability and validity of keywords:

#### 3.2.1. Initial vocabulary generation and preprocessing.

Using Python’s Jieba library, text segmentation and denoising were performed based on the integration of stop-word lists from Harbin Institute of Technology, Baidu, and ROST.CM.

#### 3.2.2. Dictionary optimization and category determination.

To enhance the validity of classification, a double-blind coding process involving two researchers was adopted. First, based on preliminary word frequency analysis and background knowledge of Sanya’s tourism, an initial classification framework covering three dimensions (cognitive, spatial, and affective) was developed. Subsequently, two coders familiar with tourism research independently categorized high-frequency words from a random sample into this framework. Classification discrepancies were resolved through discussion until a high level of consensus was reached (Cohen’s Kappa > 0.85), a process that significantly improved the content validity of the classification. Finally, we integrated the results to form a custom dictionary for Sanya’s tourism image—containing core entries—and a list of merged synonyms.

#### 3.2.3. Formal processing and word frequency statistics.

Using the finally confirmed dictionary, automated text segmentation and word frequency statistics were conducted on all texts, and the top 100 high-frequency words for each media source in each phase were extracted.

### 3.3. Compositional data analysis

The raw data collected in this study are absolute frequencies of keywords (e.g., the number of times “seafood” appears in TGC). Because different media sources have vastly different total word counts, we convert these absolute frequencies into relative frequencies (proportions) by dividing each keyword’s absolute frequency by the total frequency of all keywords in that source. These proportions sum to 1 for each source and thus constitute compositional data. Within a fixed total word volume per medium, an increase in the absolute frequency of a keyword (e.g., “seafood”) raises its own proportion and mechanically decreases the relative frequencies of all other keywords. This mutual restriction among proportions is the constant-sum constraint unique to proportional data rather than raw absolute counts. Applying traditional correlation analysis or Euclidean distance to data with such a constraint can lead to misleading results [38].

Compositional Data Analysis (CoDA) is a set of statistical methodologies specifically designed for this type of data. Through mathematical transformations such as log-ratio transformation, it maps compositional data from a “simplex space” to a real Euclidean space. This transformation enables the safe application of standard statistical tools to compare the overall image profiles across different media sources. Therefore, adopting CoDA to quantify image differences between media sources is methodologically necessary and rigorous. It can accurately address the core research question of how extensively the image structures constructed by different media sources differ overall.

Correspondence analysis (CA) is another mainstream statistical method suitable for raw keyword frequency matrices. Its core advantage is that it can natively fit the fluctuation characteristics of Poisson counts without additional zero-imputation preprocessing. Nevertheless, we still prioritize the compositional data analysis framework based on the core objectives of this study. This research aims to quantify the dissonance of proportional structures of destination image elements across different media platforms, and all keyword datasets inherently satisfy the closed constant-sum constraint of compositional data. Centered log-ratio (CLR) transformation and the derived Aitchison distance are standardized metrics specially developed to measure relative structural deviations within closed proportional systems. In contrast, correspondence analysis captures differences in absolute count distributions via chi-square distance and cannot separate compositional structural divergence from fluctuations in raw word frequencies.

Correspondence Analysis (CA) operates on raw count contingency tables using chi-square distance. Published literature has demonstrated that CA-based workflows can serve as feasible alternatives for compositional-like frequency-based datasets [43]. Nevertheless, standard CA does not explicitly address the closed constant-sum constraint central to Aitchison-style compositional-data analysis. Given our core objective of quantifying structural divergence within closed proportional keyword compositions, we adopt the centered log-ratio transformation and Aitchison distance within the CoDA framework in the present study.

Compositional data analysis encounters a key computational obstacle. Certain keywords exhibit zero frequency values, and since the natural logarithm of zero is mathematically undefined, standard log-ratio transformation cannot be directly performed. To address this issue while retaining the inherent compositional properties of the dataset, we performed standardized preprocessing using the Bayesian multiplicative zero replacement algorithm built into the R package zCompositions. This zero-imputation operation only serves as an auxiliary precondition to implement centered log-ratio transformation and subsequent Aitchison distance calculation under the compositional analysis paradigm.

Finally, it should be noted that our analysis compares only two aggregated time periods (pre-COVID vs. during-COVID). This design does not allow for statistical separation of secular time trends from pandemic-specific effects, nor does it provide sufficient temporal replications for formal significance testing. Consequently, the Aitchison distances reported in Section 4 are interpreted as descriptive measures of compositional dissimilarity between media types across the two phases, rather than as evidence of causal impact. We explicitly refrain from conducting regression-based significance tests, given the limited temporal granularity. Future studies with annual or quarterly data could extend this framework to enable rigorous hypothesis testing and trend decomposition.

#### 3.3.1. Construction of a unified analytical matrix.

To conduct cross-media comparisons of dominant destination image themes, this study adopts a standardized and reproducible procedure to select 50 core keywords as the analytical subset. First, we extract the top 100 keywords ranked by absolute frequency separately from three data sources: Press Generated Content (PGC), Tourist Generated Content (TGC), and Official Generated Content (OGC). Second, the three independent keyword lists are merged into a unified candidate pool, with duplicate keywords counted only once to eliminate data redundancy. Third, all unique keywords in the pool are sorted by their total cumulative absolute frequency across the three media types, ensuring the selected items capture the most prominent and widely recognized image attributes. Finally, the top 50 keywords with the highest overall absolute frequency are retained as the core sample set for subsequent compositional data analysis.

We further calculate keyword frequency coverage rates for the two research periods respectively, using the formula: Coverage rate = Cumulative absolute frequency of the top 50 core keywords/ Total cumulative absolute frequency of all unique keywords within the unified candidate pool. The coverage rate reaches 78.22% for Period 1 (2013–2019) and 79.23% for Period 2 (2020–2022). Liu et al. (2016) reported a heuristic empirical reference range of 70%‑80% cumulative word‑frequency coverage for keyword filtering in co‑word analysis [44]. Importantly, such coverage values are dataset‑specific and should not be treated as a universal fixed cutoff. To visually justify our keyword‑selection procedure, we provide cumulative word‑frequency‑coverage curves for each of the six dataset subsets (PGC, TGC, OGC across both time periods) based on their respective top‑100 candidate keywords (Fig 2). The curves illustrate how word frequency accumulates within each individual media‑period subset. Our selected top‑50 global keywords fall within a stable high‑coverage region and adequately represent dominant semantic features of Sanya‑tourism textual data, supporting the suitability of this keyword subset for subsequent compositional‑data analysis. In addition to frequency‑coverage metrics, semantic interpretability for cognitive‑spatial‑affective classification was also considered when determining the size of the final keyword subset. An excessively large keyword pool would introduce many low‑signal sporadic terms and compromise the reliability of dimensional classification.

**Fig 2:**
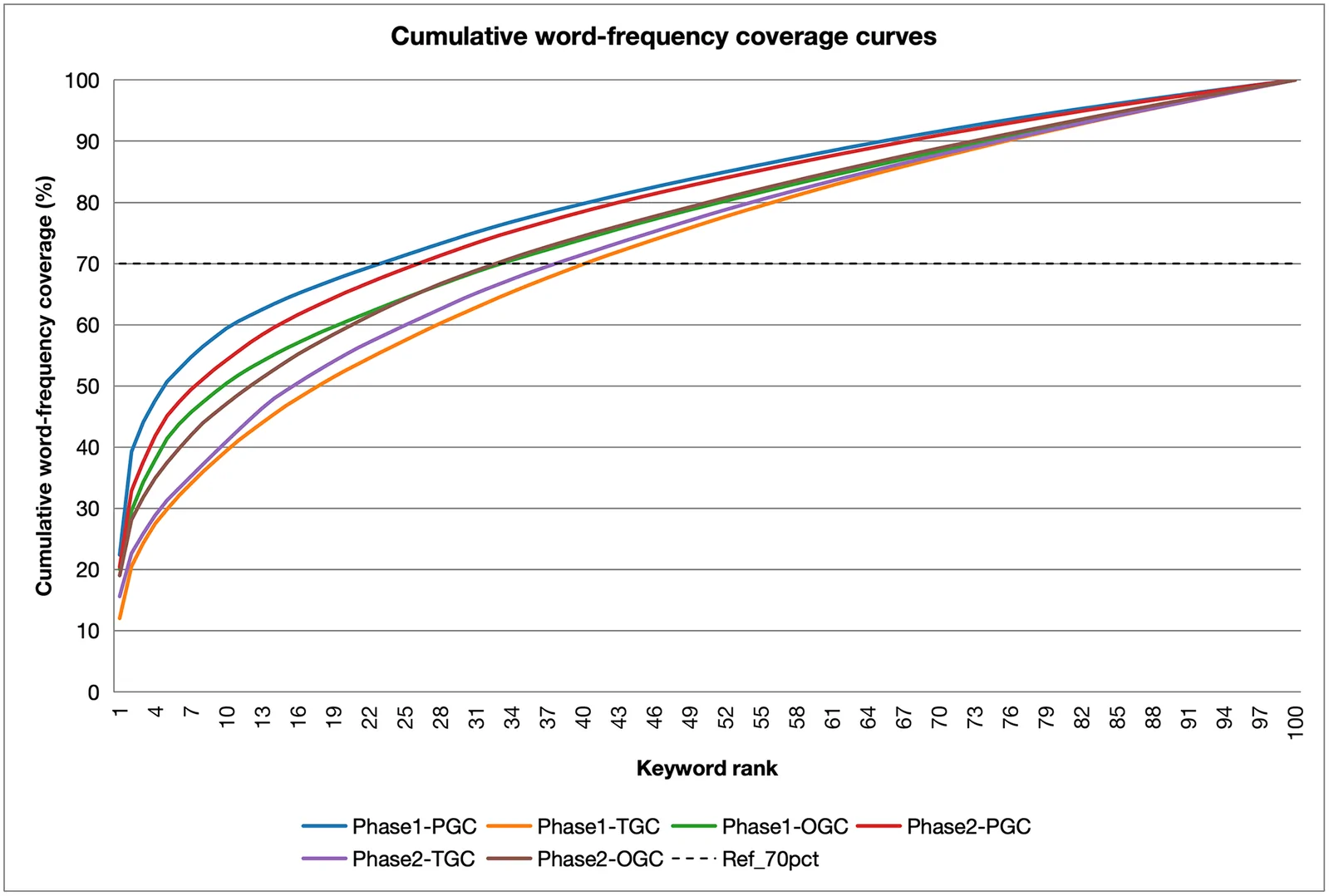
Cumulative word‑frequency‑coverage curves.

This screening procedure guarantees that the 50 core keywords are highly representative, stable, and comparable across media channels, laying a robust data foundation for subsequent Aitchison distance calculation and Dirichlet regression.

This selection criterion ensures that the image elements analyzed in this study are the most representative and prevalent across all media environments, minimizing sample selection bias and enabling the analysis to focus on core image elements processed via centered log-ratio transformation.

#### 3.3.2. Handling of zero values.

Compositional data analysis encounters a key computational obstacle. Certain keywords record zero absolute frequency within individual media datasets, and since the natural logarithm of zero is mathematically undefined, standard log-ratio transformation cannot be directly performed.

To resolve this constraint while preserving the inherent compositional characteristics of the dataset, we adopted the well-established Bayesian-multiplicative zero replacement algorithm proposed by Martín-Fernández et al. [35,45]. This method draws tiny positive values from a Dirichlet posterior distribution to substitute zero entries, and proportionally scales down non-zero components simultaneously to maintain the unit-sum constraint of compositional data. The entire zero-imputation procedure was fully implemented via the R package zCompositions (Palarea-Albaladejo & Martín-Fernández, 2015), following the complete algorithmic framework documented in the original methodological literature. This zero-handling approach maximally preserves the covariance structure of compositional data and is widely acknowledged as a standard practice for processing zero absolute counts in CoDA research.

#### 3.3.3. Calculation of Aitchison distance.

The imputed proportional data were processed through centred log-ratio transformation to eliminate distortions caused by the constant sum constraint unique to proportional data. After completing this transformation step, we calculated the Aitchison distance. This indicator is exclusively developed within the compositional data analysis system to measure the overall structural gap between two groups of proportional data, corresponding to the differentiated destination image characteristics shaped by different media sources. Larger calculated values mean more obvious divergence in the presentation of destination images between the two media channels.

## 4. Results

Aitchison distance is an important indicator for quantifying the specific structural differences in keyword proportions across media sources. A larger Aitchison distance value implies greater proportional differences, while a smaller value indicates smaller structural gaps between media. The row sums reflect the extent to which keywords help distinguish among the three media by their relative proportional compositions, and the column sums serve to distinguish the overall proportional disparities among the three media.

This study systematically measured and compared the differences in proportional structures of Sanya’s tourism image across Press Generated Content (PGC, news reports), Tourist Generated Content (TGC, tourist perceptions) and Official Generated Content (OGC, official projections) through Compositional Data Analysis (CoDA). The results clearly reveal the systematic dissonance in cross-media image representations across the three dimensions of cognitive, spatial and affective and highlight the exacerbating effect of the COVID-19 pandemic on these proportional discrepancies.

### 4.1. Cognitive image

We quantified the overall differences in cognitive images among the three types of media using Aitchison distance. As presented in Table 1 (Table 1, Main manuscript condensed table combining Table S1 and Table S2 from S1 Appendix. Full tables are available in S1 Appendix.), Press Generated Content (PGC), Tourist Generated Content (TGC), and Official Generated Content (OGC) exhibit distinct patterns where TGC shows substantial cognitive gaps with both PGC and OGC, while PGC and OGC show a high degree of consistency. Notably, the cognitive gap between TGC and OGC widened markedly after the pandemic, jumping from 19.574 to 40.648, which indicates that the crisis event has exacerbated the disconnect between official narratives and tourist experiences.

**Table 1:** Cognitive component analysis.

Stage	Contributions to distance between sources		
	P-T	P-O	T-O
2013-2019	30.462	6.939	19.574
2020-2022	38.282	5.752	40.648

As shown in Fig 3, the top 10 keywords before the epidemic were Tourism (8.080), Culture (5.826), Sea (4.530), Coconut (3.801), Service (3.102), etc. During this period, a basic yet classic coastal tourism destination image was constructed. As an alliance for the “projected image”, PGC and OGC dominated macro policy-oriented narratives such as “tourism” and “culture”. On this basis, TGC began to focus on specific natural landscapes like “sea”, “coconut”, and “beach”, as well as primary experiences such as “taste” and “swimming”. At this time, although the official narratives and tourists’ experiences had different focuses, they shared a consistent overall framework, jointly depicting a resort destination featuring sunshine, beaches and culture.

**Fig 3:**
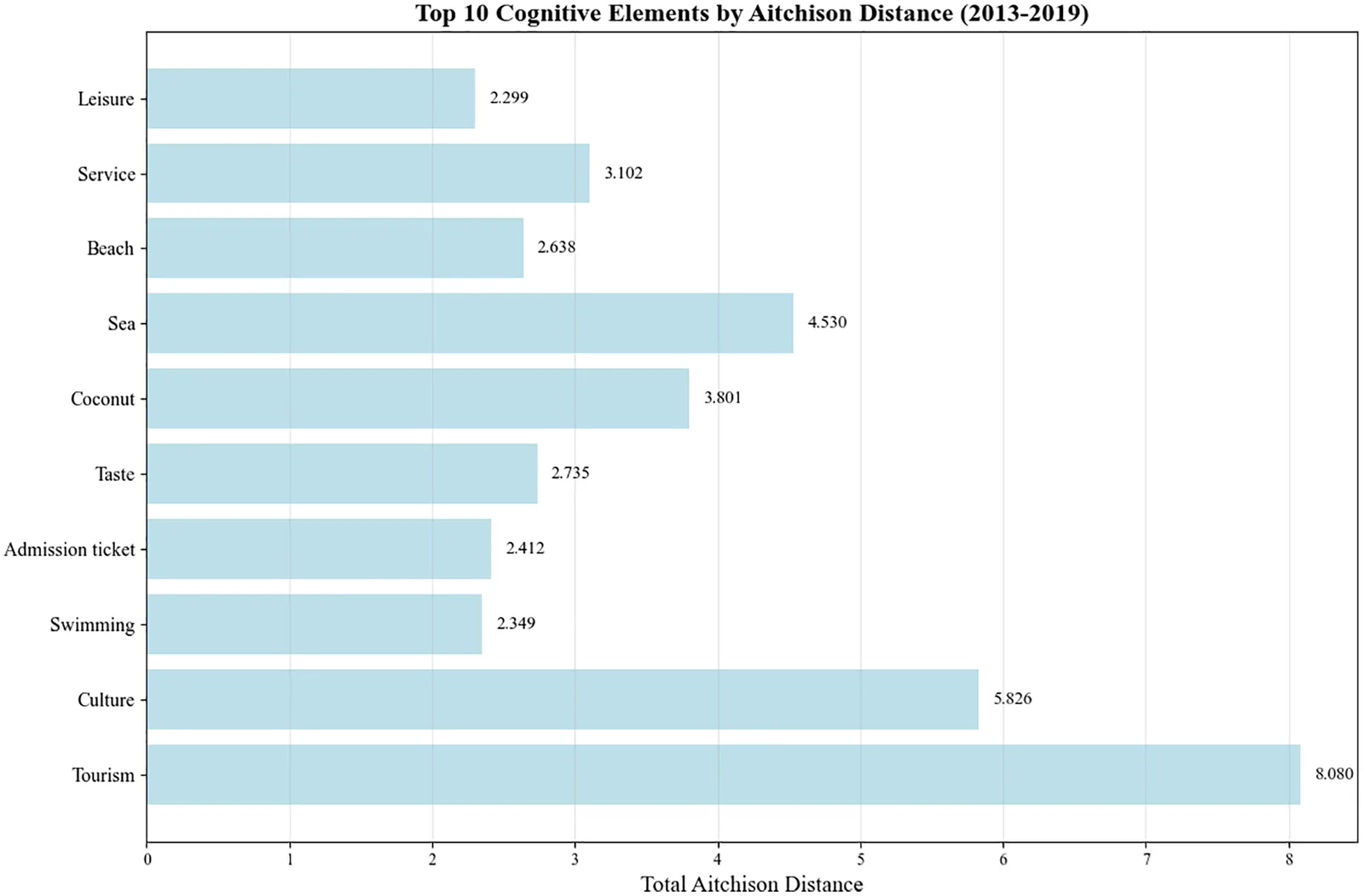
Comparison of top 10 keywords by Aitchison distance in cognitive image between two phases.

During the COVID-19 pandemic, the list of keywords underwent significant changes. Sports (8.875) and Epidemic (8.813) emerged as new keywords with the largest Aitchison distance. Meanwhile, the rankings of Seafood (5.468) and Restaurant (5.901) rose sharply, whereas the distance contributions of Tourism and Culture relatively decreased. As shown in Figs 4 and 5, the pandemic acted as a “catalyst” and “magnifying glass”.

**Fig 4:**
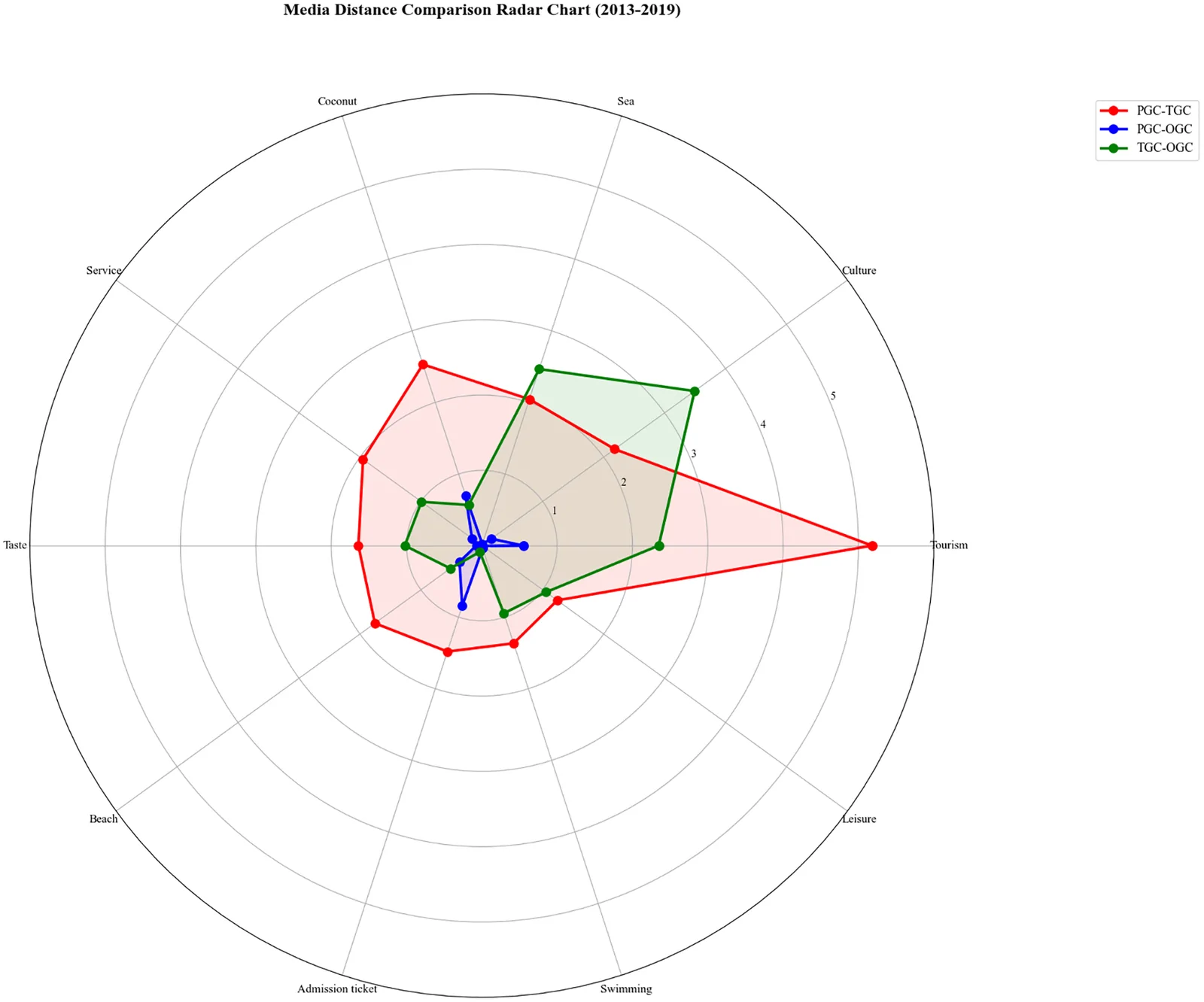
Radar chart of Aitchison distance for cognitive image.

**Fig 5:**
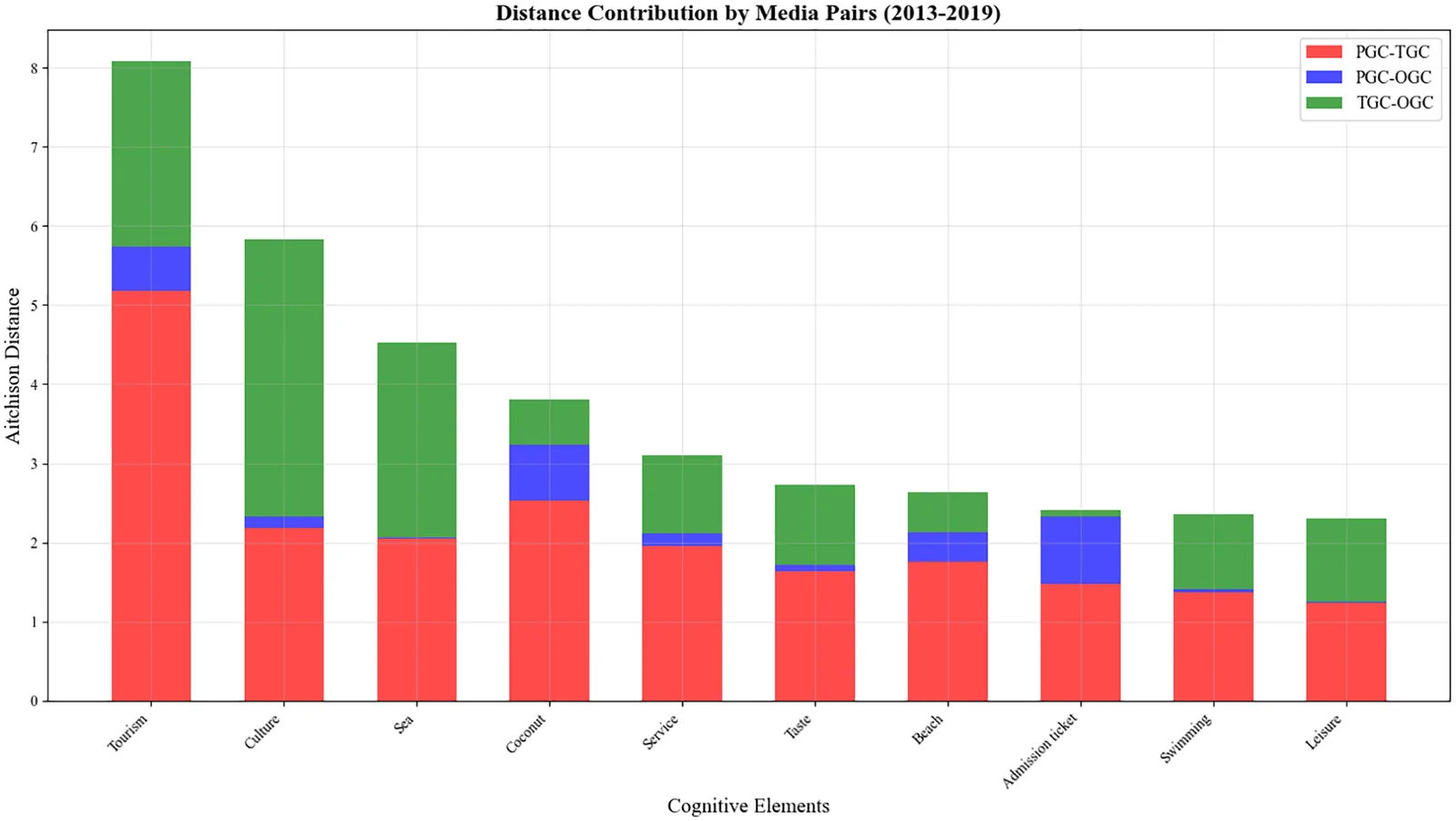
Pairwise comparative analysis chart of Aitchison distance for cognitive image across media.

Official bodies (OGC) and media outlets (PGC) shifted their focus to pandemic response: official authorities strongly promoted healthy and safe outdoor activity elements such as “sports” and “yachting”, in an attempt to reshape the destination image. At the same time, the “epidemic” itself became an unavoidable core of narratives, which led to a high degree of stability and synergy in the cognitive structures of PGC and OGC.

Fig 4 shows that there are significant differences in the cognitive profiles among the three types of media. The “sports”-oriented health image projected by official bodies (OGC) has not been effectively transformed into the core cognition of tourists. On the contrary, tourists are more concerned about localized and specific dining experiences.

The stacked bar chart in Fig 5 further indicates that from Phase 1 (2013–2019) to Phase 2 (2020–2022), the cognitive focus of TGC has evolved from a single “coastal” image to a dual-core structure of “coastal + cuisine”. This reflects the shift in tourists’ cognition from landscape sightseeing to in-depth experiences and sensory consumption, which is consistent with the upgrading of tourists’ demands under the experience economy theory. However, the cognitive structure of OGC remains stable, demonstrating the lag and inertia of institutional narratives relative to actual changes in the market.

### 4.2 Spatial image

Analysis of the spatial image reveals a significant mismatch between the spatial production and consumption of the destination. As shown in Table 2 (Table 2, Main manuscript condensed table combining Table S3 and Table S4 from S1 Appendix. Full tables are available in S1 Appendix.), the Aitchison distance between TGC and OGC became the largest among the three after the epidemic, indicating a disconnect between the space that official bodies strive to promote and the space that aligns with tourists’ actual preferences and activities.

**Table 2:** Spatial component analysis.

Stage	Contributions to distance between sources		
	P-T	P-O	T-O
2013-2019	11.798	3.326	9.276
2020-2022	12.306	2.504	12.716

Fig 6 shows that the spaces with the largest Aitchison distances are Dadonghai (3.929), Wuzhizhou Island (3.006), Banshan Peninsula (2.269), and Yalong Bay (2.198). These are traditional and well-developed core tourist areas in Sanya. During this stage, the spaces promoted by official bodies, covered by media reports, and visited by tourists largely overlapped, jointly constructing the spatial pattern of Sanya’s “Golden Coastline” for tourism.

**Fig 6:**
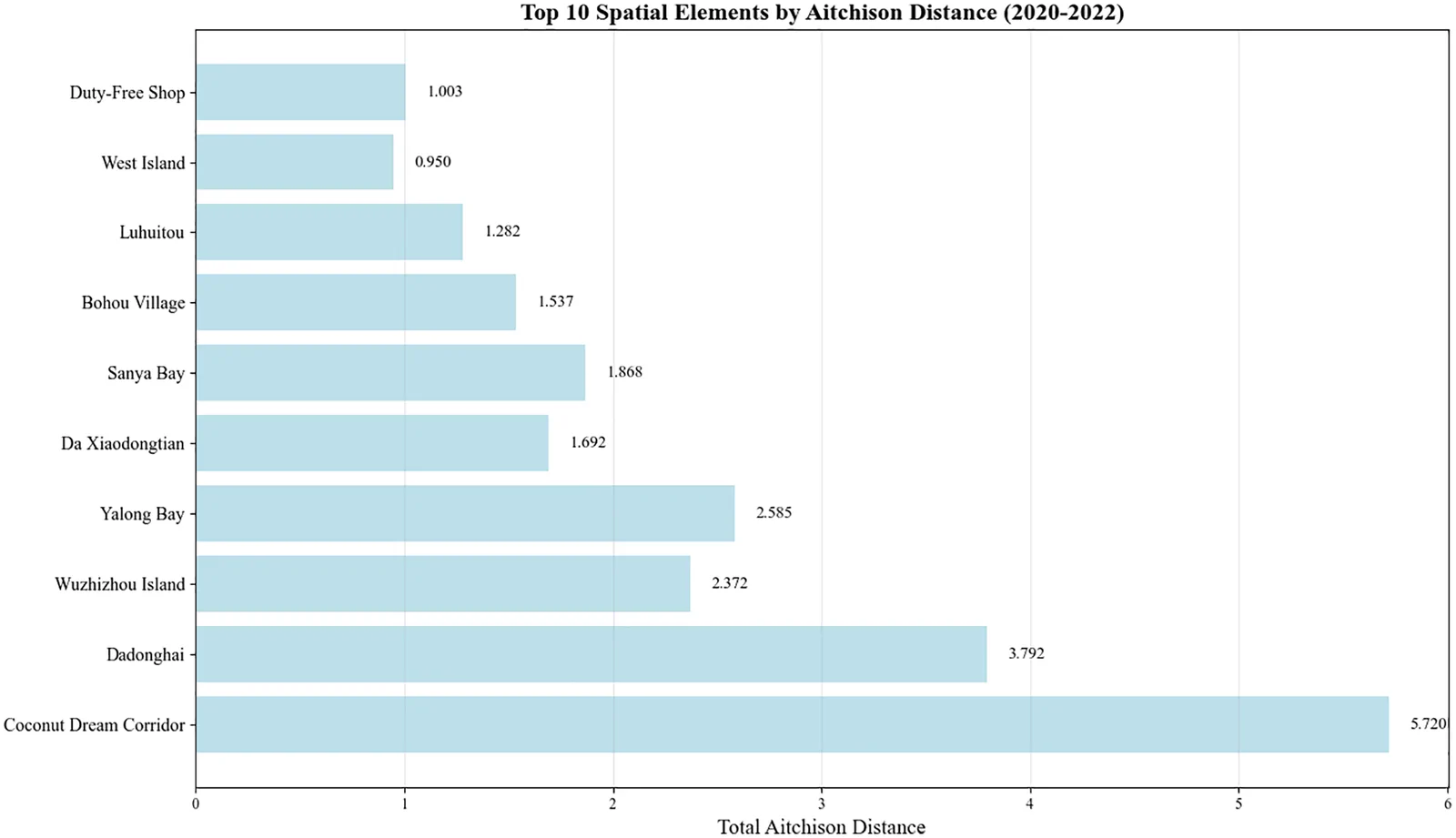
Comparison of top 10 keywords by Aitchison distance in spatial image between two phases.

The spatial image presents a profound mismatch between “production” and “consumption” (Fig 7). The “representational space” of official bodies (OGC) is manifested in OGC’s strong promotion of scenic spots such as “Duty-Free Shop” (in response to the offshore duty-free policy) and “Da Xiaodongtian”, attempting to drive spatial consumption through policy incentives. These spaces have also received positive responses in PGC, forming “policy-promoted islands”. In contrast, the “lived space” of tourists (TGC) is reflected in tourists’ de facto preferences, they favor free coastal landscape belts suitable for strolling and socializing, such as the “Coconut Dream Corridor”, as well as traditional and accessible public beaches like “Dadonghai”.

**Fig 7:**
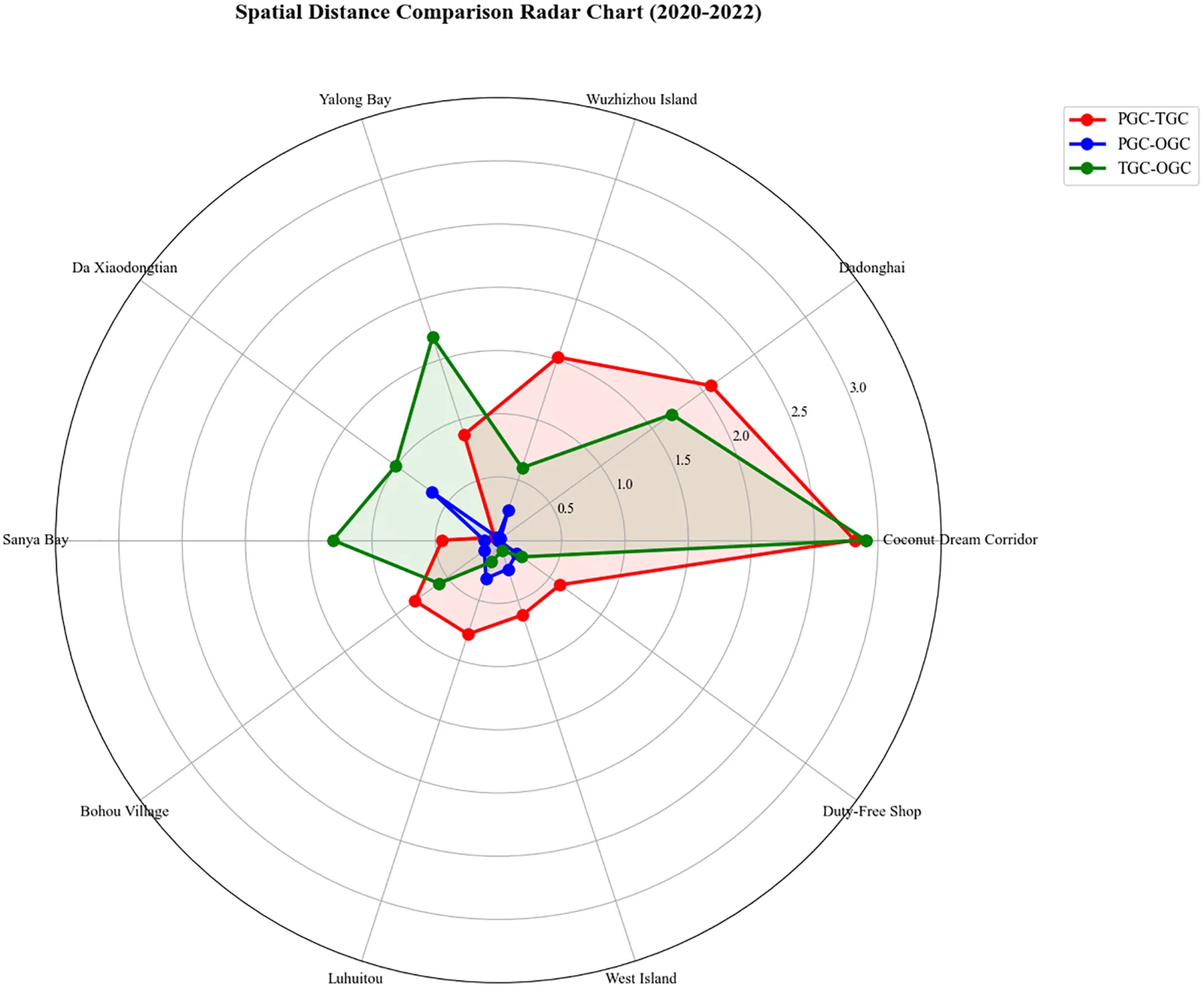
Radar chart of Aitchison distance for spatial image.

The complex profiles in Fig 8 intuitively illustrate this fragmentation. There is a severe disconnect between the space planned by official bodies (representational space) and the space that aligns with tourists’ actual experiences and preferences (lived space), and this gap has been widened after the COVID-19 pandemic.

**Fig 8:**
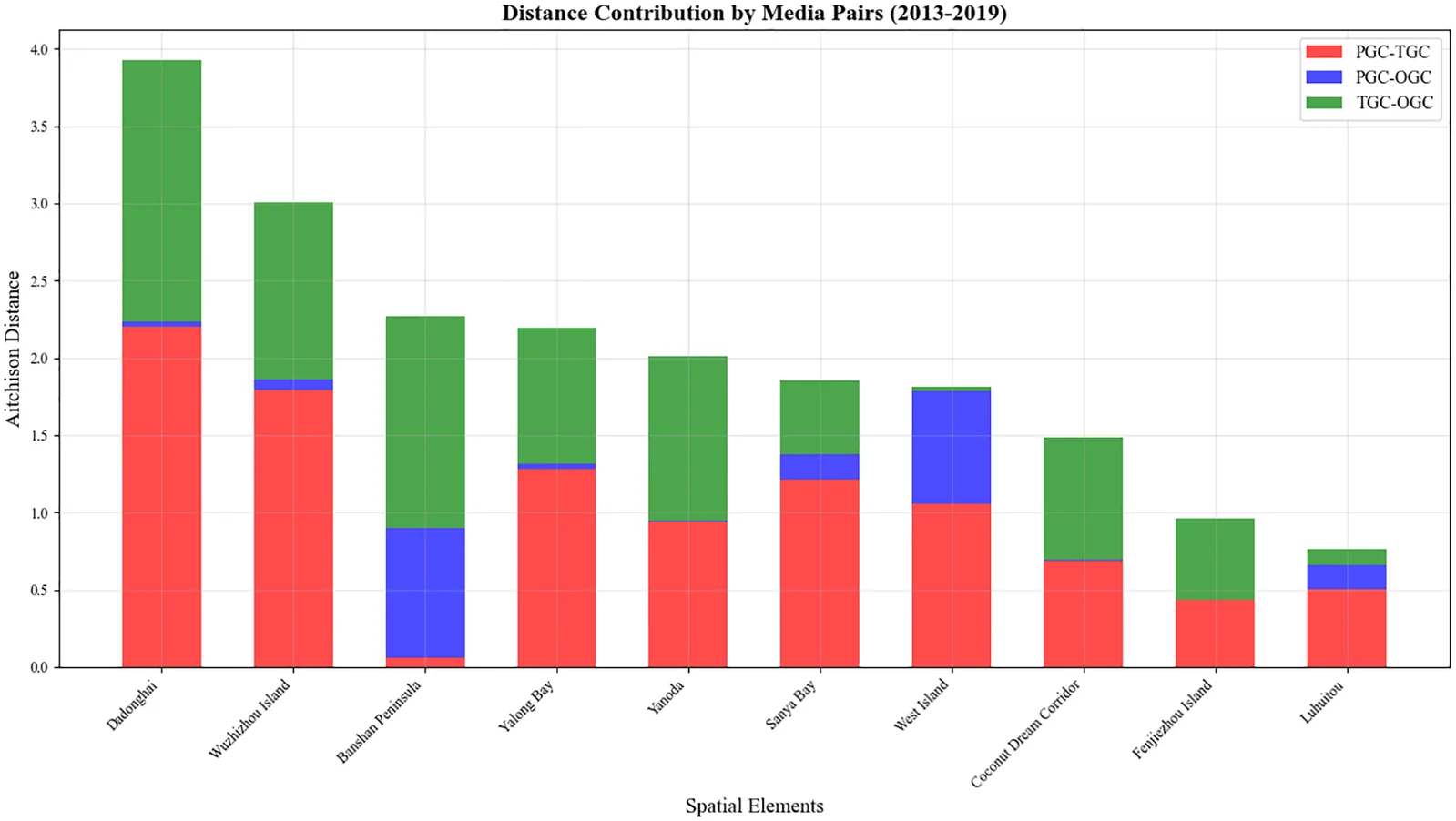
Pairwise comparative analysis chart of aitchison distance for spatial image across media.

### 4.3. Affective image

Although the overall proportion of affective keywords is relatively low, the affective image reveals the affective responses of media and tourists towards the destination. Differences in its structure point to significant marketing opportunities. As shown in Table 3 (Table 3, Main manuscript condensed table combining Table S5 and Table S6 from S1 Appendix. Full tables are available in S1 Appendix.), the affective distance between all pairs of media narrowed after the epidemic—most notably, the distance between TGC and OGC dropped from 3.551 to 1.029. This may stem from the tendency of affective expressions to become more conservative and homogeneous under the shared external pressure of the epidemic.

**Table 3:** Affective component analysis.

Stage	Contributions to distance between sources		
	P-T	P-O	T-O
2013-2019	1.383	2.046	3.551
2020-2022	0.627	1.241	1.029

As illustrated in Fig 9, “Enjoyment”, “Beauty”, and “Romance” are the shared affective keynotes, forming the destination’s positive affective image. OGC and PGC strictly control affective narratives, presenting almost exclusively curated positive emotions such as “enjoyment”, “beauty”, and “romance”. In contrast, TGC has begun to reveal more authentic and complex emotions—such as anxiety about “Queuing” and practical demands for “Convenience” (Fig 10). Here, the affective distance between TGC and OGC (as shown in Table 3) has exposed a disconnect between “managed positivity” and “authentic affective spectrum”.

**Fig 9:**
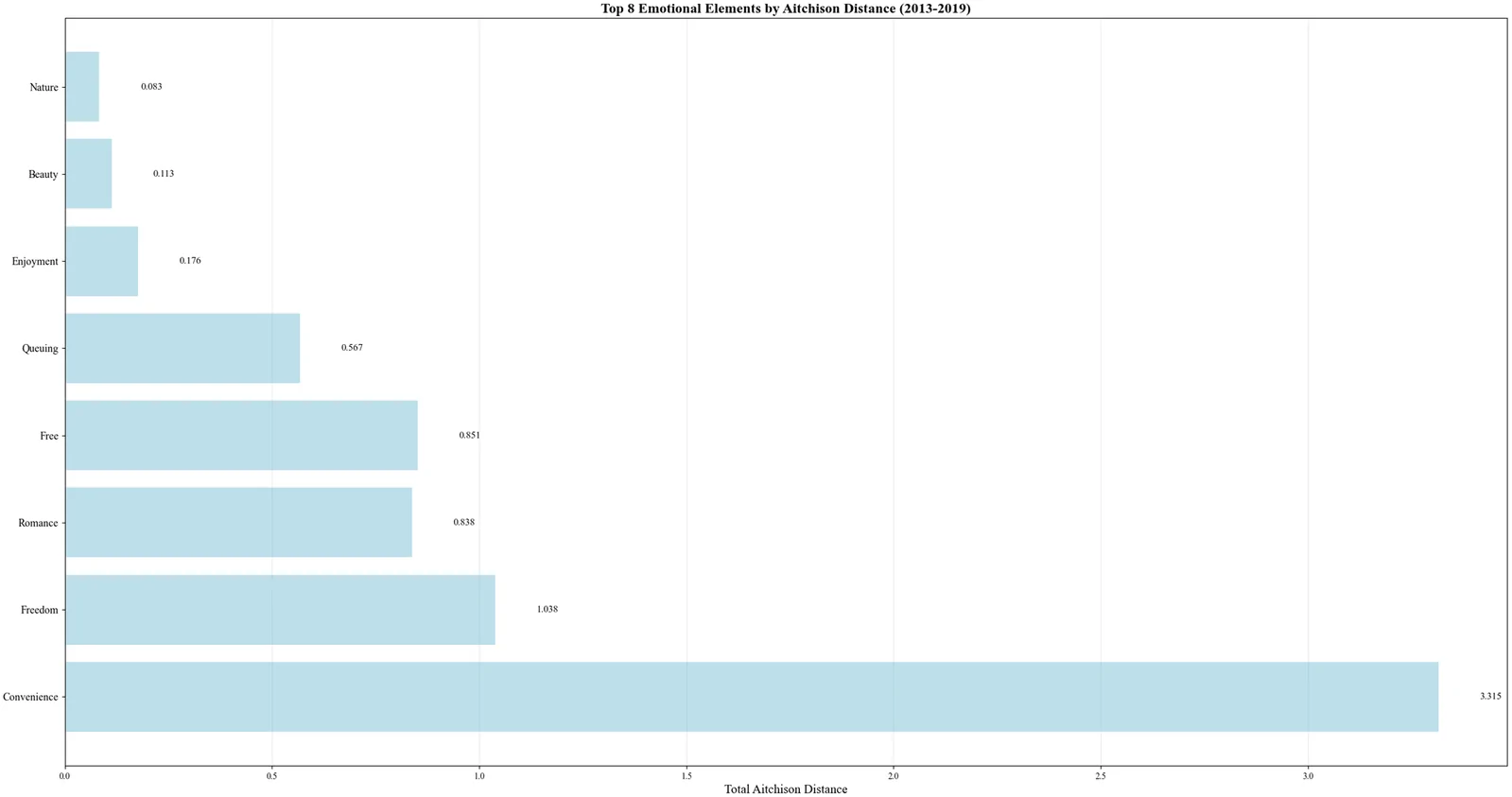
Comparison of top 8 keywords by aitchison distance in affective image between two phases.

**Fig 10:**
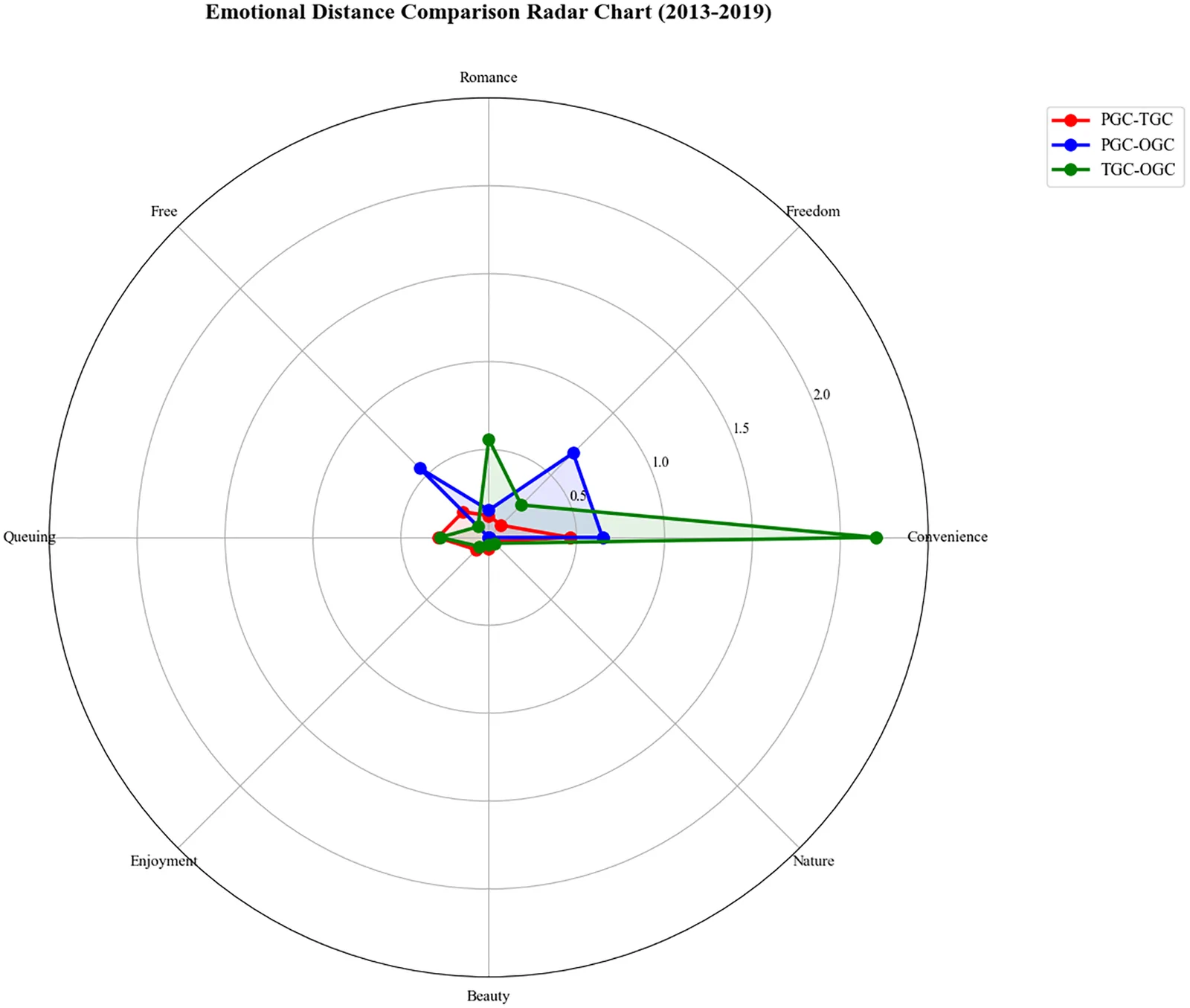
Radar chart of aitchison distance for affective image.

After the epidemic (Fig 9), a notable feature is that the Aitchison distances of all emotions have significantly narrowed across the board. “Excitement” (0.897) and “Queuing” (0.823) have become the emotions with the largest distances, while the distances for fundamental positive emotions such as “Romance” and “Beauty” have become extremely small.

Despite the narrowed distances, the differences in affective connotations remain profound. As shown in Figs 10 and 11, TGC has the most vivid affective profile—it not only retains affections like “Enjoyment” and “Beauty” but also strongly expresses the pursuit of high-arousal emotions such as “Freedom” (likely stemming from a sense of release after quarantine) and “Excitement”, as well as concerns about practical issues like “Affordability” and “Queuing”. In contrast, the affective profile of OGC is relatively simplistic and flat, almost completely suppressing key emotions expressed in TGC, such as “Excitement” and “Convenience”.

**Fig 11:**
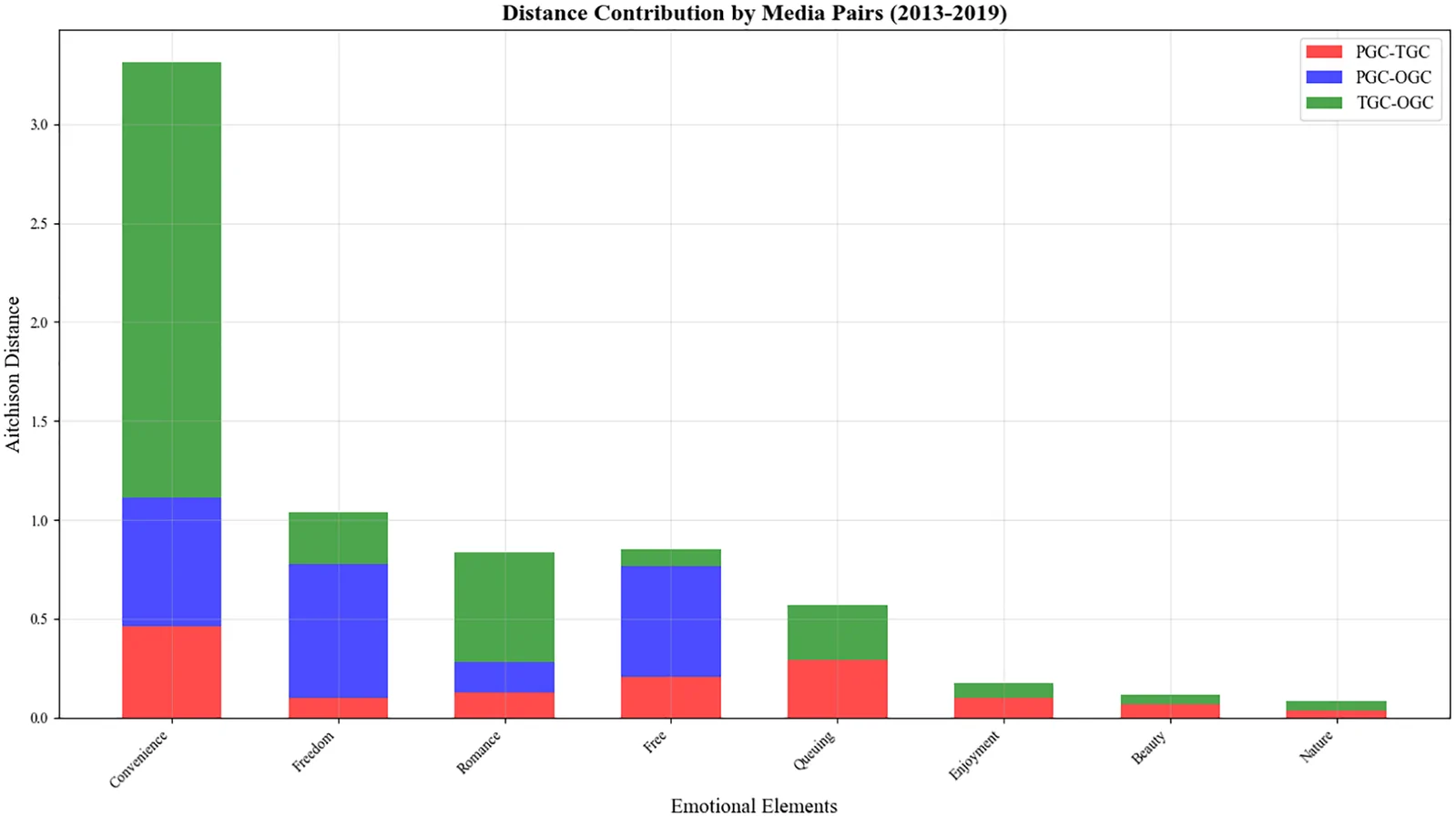
Pairwise comparative analysis chart of aitchison distance for affective image across media.

The “consistency” of the emotional image is more of a superficial phenomenon, a result of the strict narrative control imposed by OGC. The rich, dynamic, and authentic “affective spectrum” exhibited by TGC reveals an unmet gap between the affective supply from official bodies and the on-site affective needs of tourists.

## 5. Discussion

By introducing compositional data analysis (CoDA), this study conducts a ten-year longitudinal analysis of the cognitive, spatial, and emotional differences in Sanya’s tourism image across PGC (Press Generated Content), TGC (Tourist Generated Content), and OGC (Official Generated Content). The main conclusions are as follows:

The study finds that the three types of media exhibit a stable structural separation in image representation. PGC and OGC jointly form a highly consistent “institutional narrative alliance”, while TGC constitutes an independent “tourist experience narrative”. This finding aligns with Marine-Roig’s (2014) framework of multi-level construction of destination images in the Web 2.0 era [21], and empirically supports Kirilenko et al.’s (2024) view that evaluating destination images relying solely on a single platform or information source is one-sided and risky [46]. Through CoDA, this study quantifies the structural extent of such narrative separation, confirming that a destination image is not a static and unified perception but a complex construct dynamically shaped by multiple discursive systems.

Stage comparison reveals that the narrative discrepancy between TGC and OGC exhibits a notable expansion during the 2020–2022 period. Temporal difference extends Wut et al.’s (2021) research framework concerning tourism discourse evolution under crisis contexts. The comparative results indicate that turbulent public health periods not only generate new narrative elements (e.g., “epidemic”, “sports”) but also magnify pre-existing structural fractures between different stakeholder narratives [47].. Longitudinal observation further demonstrates an inherent tension between the path dependence and marketing inertia of institutional narratives in crisis response (e.g., continuing to promote policy-driven “duty-free shops” without promptly responding to tourists’ spontaneously emerging preference for “Coconut Dream Corridor”) and the on-site reality-based dynamic adaptability of tourist experiences. This systematic discourse mismatch provides localized empirical support for theories of crisis communication and destination image restoration research.

This study further unpacks the multi-dimensional imbalance of destination image across cognitive, spatial, and affective dimensions.. Cognitively, destination image presents a dual characteristic of “stable macro framework” and “fluid micro experiences”. Consistent with Li et al.’s (2023) verification of image consistency on tourist experience evaluation, this study further confirms that such cognitive divergence became more prominent during the 2020–2022 stage [27]. The deepening of TGC’s cognition from “coastal” sightseeing to “coastal + cuisine” experiences conforms to the trend of tourists pursuing emotional and sensory experiences proposed by Hosany & Gilbert (2010). However, OGC/PGC failed to respond synchronously to this change, resulting in a mismatch between the supply and demand of cognitive content [48].

The spatial image reveals a profound mismatch between the “policy-driven representational space” and the “lived space of tourist practices”, reflecting asynchronous positioning between official marketing priorities and tourists’ actual spatial preferences.. This finding supports the theory of “spatial production” in tourism geography, which posits an inherent tension between the “representation of space” shaped by official bodies and the “spatial practice” actually used by tourists [12]. Carolin et al. (2020) also observed similar spatial perception differences in their study of the Lucerne Lake area [26]; methodologically, this study further quantifies the magnitude of spatial mismatch using Aitchison distance and confirms substantial structural variations in spatial divergence between the pre-pandemic (2013–2019) and pandemic-stage (2020–2022) periods.

The affective image exposes a disconnect between “managed positivity” and the “authentic affective spectrum”. OGC strictly controls emotional narratives, almost completely suppressing key emotions expressed in TGC, such as “excitement”, “convenience”, and “queuing”. This enriches the dialogue with Stylidis et al.’s (2020) findings on the relationship between destination image, familiarity, and loyalty, demonstrating that “functional emotions” (e.g., convenience) overlooked by official bodies are also important variables affecting tourists’ on-site experiences and subsequent evaluations [49]. Notably, the reduced affective distance among different media sources in the later stage does not indicate improved narrative consistency. Instead, it reflects a convergent and conservative tendency of public and institutional emotional expression under shared external contextual disturbances.

## 6. Conclusions and implications

### 6.1. Conclusions

Through compositional data analysis of Press Generated Content (PGC), Tourist Generated Content (TGC), and Official Generated Content (OGC) related to Sanya between 2013 and 2022, this study draws the following three core conclusions:

PGC and OGC are highly consistent in image representation, jointly forming an official “institutional narrative alliance”; in contrast, TGC constitutes an independent, experience-centered “tourist narrative”. This confirms that destination image in the digital age is a complex construct differentially shaped by multiple discourse systems.

Image dissonance between TGC and OGC widened substantially in the pandemic-affected period (2020–2022) compared with the pre-pandemic phase (2013–2019), particularly in the cognitive dimension (e.g., “cuisine” vs. “sports”) and the spatial dimension (e.g., “Coconut Dream Corridor” vs. “duty-free shops”). This temporal contrast suggests that external disruptions may amplify existing narrative divides, though causal attribution awaits confirmation with higher temporal-resolution data.

Image dissonance manifests across three dimensions: cognitively, as the coexistence of stable macro-frameworks and fluid micro-experiences; spatially, as the systematic misalignment between policy-driven initiatives and tourists’ actual spatial preferences; and affectively, as the disconnect between institutionally curated positive narratives and tourists’ authentic emotional spectrum.

### 6.2. Theoretical implications

This study systematically introduces compositional data analysis (CoDA) into the field of tourism destination image (TDI) research. This provides a rigorous mathematical tool for quantifying “image differences” and, more importantly, advances the discussion on image consistency from traditional dimensions of “presence/absence” or “content” to the level of “overall structural relationships”. Through Aitchison distance, we can accurately measure the similarity of the overall image profiles across different narratives, thereby offering a new measurement dimension and theoretical language for the classic “projected-perceived” image framework.

Responding to the call from tourism geography, this study formally incorporates the spatial dimension into the core framework of image analysis. The findings indicate that space is not merely a container for image but a core component of the image itself; the misalignment in its production, dissemination, and perception constitutes a key source of image imbalance. This tripartite framework provides a more comprehensive analytical lens for future research, encouraging the TDI theory to move from dualistic opposition toward tripartite integration.

Extending beyond static image conceptualizations, this study observes that institutional narratives and tourist narratives do not evolve in full synchronously. The temporal contrast documented between the two phases suggests that institutional narratives tend to exhibit greater inertia, while tourist narratives demonstrate more dynamic responsiveness to on-the-ground experiential shifts. This finding supplements traditional theories of crisis communication and image restoration by highlighting that, in destination image management, the divergent evolutionary tendencies of institutional and tourist narratives warrant attention.

### 6.3. Practical implications

To translate the research findings into practical management applications, we propose the following specific and actionable recommendations:

Destination management organizations (DMOs) should consider developing a quarterly CoDA (Compositional Data Analysis) monitoring mechanism to operationalize the analytical approach of this study. By continuously collecting data from PGC, TGC, and OGC, practitioners can calculate Aitchison distances across key image dimensions and use these as core indicators to generate an “image health dashboard”. When the cognitive distance between TGC and OGC exceeds a pre-defined threshold (e.g., the pre-2020 benchmark level observed in this study), the system will issue early warnings to trigger timely adjustments to content strategies.

Regarding the cognitive gap between elements emphasized by TGC (such as “seafood” and “coconut”) and those promoted by OGC (such as “sports” and “yachting”), the recommended approach is scenario-based integration rather than one-way promotion. For example, DMOs could design and market themed products like “Sailing at Sea, Seeking Fresh Seafood at Fishery Markets”, which bundle officially advocated activities with tourist-favored food experiences, thereby creatively transform “institutional topics” into “tourist-centric narratives”. For spaces with high TGC popularity but low OGC promotion (e.g., “Coconut Dream Corridor”), OGC should incorporate them into officially recommended routes and enhance their visibility. For spaces with high OGC promotion but low TGC perception (e.g., Da Xiaodongtian), DMOs should carry out experience design upgrades, such as AR navigation, internet-famous photo spots, and immersive performances to bridge the “policy-promoted islands”.

TGC frequently expresses emotional keywords such as “convenience,” “queuing,” and “excitement,” which can serve as key “tourist experience touchpoints.” To address queuing-related concerns, DMOs could implement time-slot ticket reservation systems and promote “no queuing” as a marketing highlight. To meet the demand for excitement, events such as “Night Light and Shadow Art Festivals” at Coconut Dream Corridor could be organized. Through these measures, destinations can systematically translate tourists’ spontaneous affective expressions into concrete experience optimization and sensory marketing strategies.

### 6.4. Limitations and future directions

#### 6.4.1. Research limitations.

While CoDA (Compositional Data Analysis) excels at quantifying differences, it is inherently unable to reveal the causal relationships behind the formation of these differences. This study identifies the existence and extent of image imbalance, but the exploration of the complex social-psychological mechanisms underlying it remains superficial, precluding verification of how media image disparities influence travel decisions. Although the “institution-tourist” narrative analysis framework proposed in this paper reveals the role of power in image construction, it does not incorporate the narratives of other important stakeholders, such as local residents and tourism practitioners.

Several limitations of this study should be acknowledged. First, the temporal structure of our data is configured to compare two aggregated phases (2013–2019 and 2020–2022), which serves the primary objective of profiling stage-level contrasts in image composition. By design, this approach prioritizes cross-phase structural comparison over temporal disaggregation; it does not, however, permit statistical separation of secular time trends from pandemic-period fluctuations. The Aitchison distance expansions observed between the two phases are therefore offered as descriptive temporal contrasts rather than causal estimates of pandemic effects.

Second, the CoDA framework entails a specific computational trade-off. The log-ratio transformation is undefined for zero values; to address this, we followed standard practice by applying Bayesian-multiplicative imputation. While this procedure is widely accepted in compositional data analysis, it inevitably introduces minor estimation bias for keywords entirely absent from certain media sources. We have made both the raw absolute frequency matrices and the imputed proportional datasets publicly available on GitHub to ensure full transparency and replicability. It is also worth noting that CoDA, by its nature, quantifies structural dissimilarities in proportional compositions rather than uncovering the social-psychological mechanisms underlying these differences—an important question that awaits further qualitative or experimental investigation.

#### 6.4.2. Future research directions.

Future studies could extend the current framework in several directions. First, the use of annual or quarterly data would allow researchers to disentangle long-term trends from episodic shocks, enabling more robust causal attribution of how external events shape image dissonance over time. Second, questionnaire surveys or experimental designs could be introduced to develop and test structural equation models linking image dissonance to tourist expectations, satisfaction, and loyalty, thereby moving beyond correlational description toward causal explanation. Third, the integration of multi-modal data, including social media images, short videos, and audio materials alongside computer vision and audio sentiment analysis, could construct a more immersive “text-vision-audio” image model.

## Supporting information

S1 Appendix Six full tables underlying condensed Tables 1–3 in the main manuscript. (DOCX)
