# Supplementary material for: Measuring destination image dissonance: A compositional data analysis of cross-media representations in Sanya’s Tourism
Source: PLoS One. 2026 Oct 15;21(10):e0360383. doi: 10.1371/journal.pone.0360383 (PMC13671502; doi:10.1371/journal.pone.0360383)
Supplement: Supplementary file 1 [file pone.0360383.s003.pdf]

Review 1

1. Introduction

Weaknesses:

1. The opening paragraph is too descriptive and contains the history of TDI theory without explicitly emphasizing the *research gap* at the end of the section.
2. The logical flow towards the research goals is rather abrupt — the explanation of the "cognitive–affective–spatial" concept and the transition to the use *of Compositional Data Analysis (CoDA)* are less connected to the emptiness of previous research.
3. No *clear research question* or hypothesis is explicitly stated.

Suggestions for improvement:

Focus the introduction on *the problem statement*, show empirical gaps related to dissonance between media, and explain the theoretical and practical contributions of the research with a declarative sentence at the end of the section.

2. Literature Review

Weaknesses:

1. The structure of the chronological sub-chapters (period 1973–2015) is historical but less analytical; it has not shown a direct relationship with the research variables (cognitive-spatial-emotional).
2. Some classic quotes (Echtner 1991; Gartner 1993) are not updated with post-2020 literature relevant to *big data* and *cross-media analytics*.
3. The *Gaps in Representation* section mentions the gap between sources but does not affirm *why compositional data* is a methodological solution.

Suggestions for improvement:

Add cross-dimensional conceptual synthesis (cognitive–spatial-emotional) and CoDA contribution compared to traditional methods (content analysis, semantic networking, topic modeling). View a literature summary table to reinforce the gap argument.

3. Methodology

Weaknesses:

1. The technical explanation of CoDA is very detailed (formulas, transformations, formulas) but too mathematical without adequately explaining *why this method fits tourism data*.
2. The validity and reliability of *the keyword extraction* process (dictionary, stopword, synonym list) have not been empirically tested (e.g., inter-coder or pretest).
3. The criteria for selecting 50 *common keywords are not explained* — the potential for selection bias is quite high.
4. Spatial analysis is less equipped with visualizations (maps or heatmaps).

Suggestions for improvement:

Add the methodological reasons for selecting the CoDA, the data validation procedure, and include *the analysis flowchart*. Explain how the interpretation of results is linked to TDI theory, not just proportional statistics.

4. Results

Weaknesses:

1. The results are presented very long and the tables are dense with numbers, causing difficulties in capturing conceptual meaning.
2. Narrative analysis tends to be descriptive (calling it "top five keywords") without directly relating to the theory of destination perception or tourist behavior.
3. No significance tests or robustness were tested between periods.
4. Visualizations (graphs, Aitchison distance charts) are not available, although it is important to clarify cross-media dissonance.

Suggestions for improvement:

Summarize the numerical results with a comparison graph and add theoretical interpretations (e.g., implications for brand image consistency). Include additional analysis, such as clusters or *trend evolution* to reinforce the findings.

5. Conclusions and Implications

Weaknesses:

1. The conclusions affirm the existence of an "institutional-tourist narrative misalignment" but do not elaborate on its scientific contribution to the theory of TDI or tourism communication.
2. The policy implications are still general, not showing how destination governments can use the results of the CoDA operationally.
3. There are no limitations and future research agendas.

Suggestions for improvement:

Clarify the theoretical (e.g., integration of CoDA in the destination image framework) and practical (bridging strategies between OGC-TGC-PGC). Add a limitations *and future research directions section*.
