# Supplementary material for: Measuring destination image dissonance: A compositional data analysis of cross-media representations in Sanya’s Tourism
Source: PLoS One. 2026 Oct 15;21(10):e0360383. doi: 10.1371/journal.pone.0360383 (PMC13671502; doi:10.1371/journal.pone.0360383)
Supplement: Supplementary file 2 [file pone.0360383.s008.pdf]

Review of Measuring Destination Image Dissonance

The paper has a good point to make, namely that it is useful to understand different types of image disparity across cognitive, spatial and affective dimensions, but there are some serious issues that must be addressed before the paper is ready for publication

1. The abstract refers to a time lag, but the analysis fails to include the temporal dimension properly – it is impossible in this analysis to distinguish time trends from COVID impacts, making all the conclusions of COVID impact impossible to verify. This can be addressed by using yearly data, or at the very least dividing the pre-COVID period into multiple time periods. It would also greatly strengthen the analysis to add a post-COVID period, to see whether the disparities reduce.
2. The claim that the data is all available in gitlab is simply untrue. There is no labelling of the 50 words, in particular, as to which are cognitive, spatial and affective, let alone which link to the specific elements referenced in the analysis. Tourism, hotels, coastal food are referenced in the abstract without any connection to the data set provided! The dataset is already converted to relative frequencies and zeros removed, making it impossible to understand (for example), how the zero replacement affects the results. The dataset must include raw frequencies with full labellling provided.
3. Compositional data analysis (CoDA) (lines 130 onwards) is one way, not the only way to examine relative frequencies and correspondence analysis (CA) has a very specific advantage over CoDA when starting with frequency data in that it accounts for the count variability (i.e. for Poisson count data, variance is proportional to the mean) and avoids any need to make adjustments for zeros. This should be referenced as an alternative which avoids the problems of zeros and yet also provides a distance metric.
4. The paper confuses frequencies and relative frequencies in several places (see line 125) – relative frequencies add to 1 and hence have the compositional constraint, but this is not true for frequencies.
5. Data sources – there is no justification provided for the specific 3 sources chosen (e.g. what proportion of travellers use Ctrip?)
6. The paper makes the claim (line 283) that the criterion ensures the elements are the most prevalent, which is true, but fails to address whether 50 key words are sufficient, for example, what proportion of all key word references are included in the 50 key words selected?
7. Formulas 2 and 3 are both incomplete – what is s_i and what are the summation limits? Formula 3 is clearly faulty.
8. Unsupported claims (see line 352) – as mentioned above, this analysis can NOT distinguish time trends from COVID impacts.
9. The Dirichlet regression (lines 470 to 507) is clearly faulty – it ignores that Dirichlet models require conditional independence (see the Aitchison reference) and regression requires independent errors. Further, there are really only 2 data points as there are only 2 time periods, so I have no idea what analysis was done (and it is impossible to tell, given the dataset provided). The discussion linked to this analysis is hence clearly faulty as well.
