# Supplementary material for: Measuring destination image dissonance: A compositional data analysis of cross-media representations in Sanya’s Tourism
Source: PLoS One. 2026 Oct 15;21(10):e0360383. doi: 10.1371/journal.pone.0360383 (PMC13671502; doi:10.1371/journal.pone.0360383)
Supplement: Supplementary file 3 [file pone.0360383.s010.pdf]

Review of Measuring Destination Image Dissonance

The revision is greatly improved and address my major concerns – my 4 remaining minor concerns are

1. Abstract should initially use the full forms of PGC, TGC and OGC.
2. Comment 2 response sounds good, but "All data files are fully annotated with readme documents to ensure reproducibility of all keyword screening and CoDA procedures" is incorrect - there are no annotated readme documents and the labels are only in Chinese (no English, making it infeasible to match with the text of the paper). This must be fixed.
3. Comment 3 makes the incorrect claim that "In contrast, CA mainly identifies differences in raw count distributions based on chi-square distance, which cannot independently extract structural deviations of proportional compositions". There is no CA reference, suggesting that the authors simply do not know the CA literature. I would point the authors to this openly accessible paper:
   https://econ-papers.upf.edu/papers/1857.pdf
   which has subsequently been published in a revised form and illustrates that CA **can** be used instead of CoDA.
4. Comment 6 makes the incorrect claim that "cumulative word frequency coverage of 70% acts as the critical cutoff separating informative high- and medium-frequency terms from trivial long-tail low-frequency words."
   It is well known that the appropriate cutoff for word frequency coverage depends on the dataset and a plot of the cumulative coverage is the best tool to show that the cutoff is appropriate, so the reference is simply incorrect in the claim. I suggest adding a graph to show that the cutoff is appropriate for these 3 datasets.
