# Supplementary material for: Measuring destination image dissonance: A compositional data analysis of cross-media representations in Sanya’s Tourism
Source: PLoS One. 2026 Oct 15;21(10):e0360383. doi: 10.1371/journal.pone.0360383 (PMC13671502; doi:10.1371/journal.pone.0360383)
Supplement: Supplementary file 4 [file pone.0360383.s001.docx]

S1 Appendix. Six full tables underlying condensed Tables 1–3 in the main manuscript

Table S1 Cognitive component analysis from 2013 to 2019

|  | Percentage of keywords (%) within data source | | | Contributions to distance between sources | | | Row  sum |
| --- | --- | --- | --- | --- | --- | --- | --- |
|  | PGC | TGC | OGC | P-T | P-O | T-O |  |
| Fruits | 0.607% | 2.503% | 1.020% | 1.220 | 0.089 | 0.649 | 1.958 |
| Culture | 5.054% | 1.578% | 9.331% | 2.180 | 0.154 | 3.492 | 5.826 |
| Service | 5.132% | 1.736% | 4.284% | 1.950 | 0.161 | 0.991 | 3.102 |
| Leisure | 1.654% | 0.743% | 1.892% | 1.238 | 0.007 | 1.053 | 2.299 |
| Tropical rainforest | 0.397% | 1.013% | 0.786% | 0.390 | 0.213 | 0.026 | 0.629 |
| Mango | 1.223% | 1.505% | 0.879% | 0.011 | 0.303 | 0.199 | 0.513 |
| Diving | 1.202% | 2.346% | 1.395% | 0.126 | 0.005 | 0.183 | 0.315 |
| Countryside | 1.132% | 0.000% | 1.283% | 0.542 | 0.009 | 0.410 | 0.961 |
| Parent-child | 0.742% | 2.126% | 0.937% | 0.549 | 0.000 | 0.530 | 1.079 |
| Hot spring | 0.689% | 1.201% | 1.504% | 0.059 | 0.314 | 0.101 | 0.473 |
| Restaurant | 0.669% | 2.586% | 1.119% | 1.082 | 0.087 | 0.556 | 1.725 |
| Shopping | 1.670% | 0.936% | 0.935% | 0.795 | 0.641 | 0.008 | 1.444 |
| Tourism | 43.269% | 6.075% | 25.651% | 5.179 | 0.553 | 2.348 | 8.080 |
| Hotel | 9.031% | 23.354% | 9.741% | 0.406 | 0.021 | 0.612 | 1.040 |
| Scenic spot | 7.874% | 4.556% | 6.284% | 0.739 | 0.199 | 0.171 | 1.109 |
| Vacation | 4.449% | 4.727% | 4.038% | 0.064 | 0.101 | 0.004 | 0.169 |
| Price | 3.897% | 3.859% | 5.166% | 0.104 | 0.004 | 0.147 | 0.255 |
| Seafood | 2.957% | 8.677% | 4.421% | 0.584 | 0.033 | 0.339 | 0.956 |
| Beach | 1.248% | 6.416% | 2.852% | 1.755 | 0.367 | 0.517 | 2.638 |
| Cuisine | 1.015% | 2.319% | 2.441% | 0.264 | 0.431 | 0.021 | 0.715 |
| Ocean | 1.066% | 0.762% | 1.597% | 0.419 | 0.034 | 0.691 | 1.144 |
| Facility | 0.860% | 0.975% | 0.931% | 0.035 | 0.020 | 0.002 | 0.057 |
| Park | 0.700% | 1.182% | 1.336% | 0.045 | 0.181 | 0.046 | 0.271 |
| Sunshine | 0.687% | 1.777% | 1.422% | 0.407 | 0.257 | 0.017 | 0.682 |
| Fruits | 0.607% | 2.503% | 1.020% | 1.220 | 0.089 | 0.649 | 1.958 |
| Li nationality | 0.593% | 0.952% | 2.001% | 0.026 | 0.990 | 0.698 | 1.714 |
| Island | 0.575% | 0.692% | 0.732% | 0.016 | 0.000 | 0.022 | 0.038 |
| Swimming | 0.000% | 1.910% | 0.658% | 1.365 | 0.038 | 0.946 | 2.349 |
| Sea | 0.484% | 2.762% | 0.524% | 2.044 | 0.020 | 2.466 | 4.530 |
| Admission ticket | 0.516% | 2.379% | 1.610% | 1.480 | 0.843 | 0.089 | 2.412 |
| Coconut | 0.000% | 2.755% | 1.183% | 2.531 | 0.702 | 0.567 | 3.801 |
| Taste | 0.000% | 3.092% | 1.026% | 1.639 | 0.072 | 1.023 | 2.735 |
| Column totals (percentage and sum of squared Aitchison distances) | 100% | 100% | 100% | 30.462 | 6.939 | 19.574 |  |

Table S2 Cognitive component analysis from 2020 to 2022

|  | Percentage of keywords (%) within data source | | | Contributions to distance between sources | | | Row  sum |
| --- | --- | --- | --- | --- | --- | --- | --- |
|  | PGC | TGC | OGC | P-T | P-O | T-O |  |
| Beautiful scenery | 0.534% | 1.124% | 0.662% | 0.278 | 0.011 | 0.179 | 0.468 |
| Tourism | 32.716% | 6.323% | 25.066% | 3.460 | 0.142 | 2.200 | 5.802 |
| Hotel | 11.068% | 18.108% | 6.974% | 0.076 | 0.328 | 0.719 | 1.123 |
| Scenic spot | 8.574% | 4.834% | 8.509% | 0.623 | 0.014 | 0.451 | 1.088 |
| Service | 5.914% | 1.681% | 5.927% | 2.174 | 0.012 | 1.866 | 4.051 |
| Culture | 5.296% | 1.675% | 10.191% | 1.871 | 0.296 | 3.655 | 5.821 |
| Seafood | 1.449% | 8.442% | 1.328% | 2.390 | 0.039 | 3.039 | 5.468 |
| Coconut | 0.000% | 5.059% | 0.000% | 2.883 | 0.014 | 3.294 | 6.191 |
| Restaurant | 1.033% | 4.986% | 0.650% | 1.843 | 0.329 | 3.729 | 5.901 |
| Beach | 1.366% | 4.964% | 2.733% | 1.154 | 0.340 | 0.241 | 1.735 |
| Vacation | 3.088% | 4.901% | 2.317% | 0.060 | 0.158 | 0.414 | 0.632 |
| Cuisine | 1.294% | 4.683% | 1.251% | 1.144 | 0.021 | 1.475 | 2.640 |
| Event | 0.998% | 0.000% | 1.894% | 0.406 | 0.282 | 1.365 | 2.053 |
| Sea | 0.487% | 3.189% | 0.648% | 2.766 | 0.031 | 2.214 | 5.010 |
| Diving | 1.057% | 2.401% | 1.214% | 0.365 | 0.001 | 0.332 | 0.698 |
| Price | 1.995% | 2.301% | 1.147% | 0.005 | 0.442 | 0.349 | 0.796 |
| Sunshine | 0.463% | 2.289% | 0.943% | 1.908 | 0.361 | 0.609 | 2.878 |
| Check in | 1.081% | 2.081% | 1.278% | 0.192 | 0.003 | 0.145 | 0.341 |
| Island | 0.618% | 1.749% | 1.000% | 0.680 | 0.138 | 0.205 | 1.024 |
| Homestay | 3.895% | 1.572% | 3.034% | 1.264 | 0.130 | 0.583 | 1.977 |
| Child | 0.463% | 1.568% | 0.922% | 1.007 | 0.334 | 0.181 | 1.522 |
| Yacht | 3.800% | 1.445% | 4.272% | 1.401 | 0.000 | 1.416 | 2.817 |
| Admission ticket | 0.736% | 1.331% | 1.466% | 0.141 | 0.334 | 0.041 | 0.516 |
| Epidemic | 4.441% | 0.620% | 4.126% | 4.776 | 0.034 | 4.004 | 8.813 |
| Surfing | 2.114% | 1.112% | 1.665% | 0.738 | 0.122 | 0.260 | 1.120 |
| Seaview | 1.366% | 4.964% | 2.733% | 1.154 | 0.340 | 0.241 | 1.735 |
| Sightseeing | 0.986% | 1.559% | 1.061% | 0.059 | 0.001 | 0.078 | 0.138 |
| Sailing | 0.713% | 1.086% | 1.061% | 0.042 | 0.083 | 0.007 | 0.131 |
| Tropical rainforest | 0.523% | 2.006% | 0.599% | 1.274 | 0.001 | 1.217 | 2.492 |
| Sports | 1.164% | 0.000% | 3.411% | 2.106 | 0.931 | 5.837 | 8.875 |
| Internet celebrity | 0.772% | 0.993% | 0.622% | 0.001 | 0.106 | 0.130 | 0.238 |
| Li nationality | 0.000% | 0.953% | 1.296% | 0.040 | 0.377 | 0.171 | 0.587 |
| Column totals (percentage and sum of squared Aitchison distances) | 100% | 100% | 100% | 38.282 | 5.752 | 40.648 |  |

Table S3 Spatial component analysis from 2013 to 2019

|  | Percentage of keywords (%) within data source | | | Contributions to distance between sources | | | Row  sum |
| --- | --- | --- | --- | --- | --- | --- | --- |
|  | PGC | TGC | OGC | P-T | P-O | T-O |  |
| Sanya | 79.833% | 42.189% | 75.901% | 0.758 | 0.015 | 0.561 | 1.334 |
| Yalong Bay | 3.442% | 13.469% | 4.485% | 1.280 | 0.037 | 0.880 | 2.198 |
| Haitang Bay | 3.391% | 4.052% | 3.334% | 0.003 | 0.008 | 0.001 | 0.012 |
| Nanshan Temple | 2.335% | 2.178% | 2.093% | 0.092 | 0.033 | 0.015 | 0.139 |
| Tianya Haijiao  (End of the Earth) | 2.068% | 4.222% | 2.806% | 0.231 | 0.055 | 0.061 | 0.346 |
| Sanya Bay | 1.808% | 6.858% | 2.917% | 1.210 | 0.166 | 0.481 | 1.857 |
| Dadonghai | 1.101% | 6.130% | 1.420% | 2.203 | 0.034 | 1.693 | 3.929 |
| Luhuitou  (Deer Turning Head) | 0.869% | 2.234% | 1.378% | 0.506 | 0.152 | 0.103 | 0.761 |
| West Island | 0.574% | 2.024% | 1.447% | 1.055 | 0.728 | 0.030 | 1.813 |
| Wuzhizhou Island | 1.396% | 6.716% | 1.962% | 1.791 | 0.072 | 1.142 | 3.006 |
| Atlantis | 1.440% | 0.972% | 0.000% | 0.392 | 0.732 | 0.053 | 1.177 |
| Duty-Free Shop | 1.167% | 1.783% | 0.000% | 0.036 | 0.214 | 0.426 | 0.676 |
| Phoenix Island | 0.577% | 0.972% | 1.000% | 0.083 | 0.228 | 0.036 | 0.347 |
| Coconut Dream Corridor | 0.000% | 1.436% | 0.000% | 0.688 | 0.004 | 0.792 | 1.484 |
| Fenjiezhou Island | 0.000% | 0.982% | 0.000% | 0.435 | 0.004 | 0.519 | 0.958 |
| Tropical Paradise Forest Park | 0.000% | 1.479% | 0.000% | 0.028 | 0.004 | 0.052 | 0.084 |
| Yanoda | 0.000% | 2.306% | 0.000% | 0.943 | 0.004 | 1.065 | 2.012 |
| Banshan Peninsula | 0.000% | 0.000% | 1.259% | 0.064 | 0.838 | 1.366 | 2.269 |
| Column totals (percentage and sum of squared Aitchison distances) | 100% | 100% | 100% | 11.798 | 3.326 | 9.276 |  |

Table S4 Spatial component analysis from 2020 to 2022

|  | Percentage of keywords (%) within data source | | | Contributions to distance between sources | | | Row  sum |
| --- | --- | --- | --- | --- | --- | --- | --- |
|  | PGC | TGC | OGC | P-T | P-O | T-O |  |
| Sanya | 77.35% | 51.48% | 76.13% | 0.34 | 0.001 | 0.326 | 0.667 |
| Yalong Bay | 3.02% | 9.75% | 2.62% | 0.877 | 0.021 | 1.687 | 2.585 |
| Wuzhizhou Island | 1.42% | 6.11% | 2.35% | 1.522 | 0.248 | 0.602 | 2.372 |
| Sanya Bay | 2.47% | 5.78% | 1.77% | 0.445 | 0.114 | 1.309 | 1.868 |
| Haitang Bay | 2.54% | 5.26% | 2.05% | 0.297 | 0.047 | 0.581 | 0.925 |
| Dadonghai | 0.75% | 3.83% | 0.87% | 2.079 | 0.020 | 1.693 | 3.792 |
| Tianya Haijiao  (End of the Earth) | 2.79% | 3.18% | 2.96% | 0.003 | 0.003 | 0.012 | 0.018 |
| Luhuitou  (Deer Turning Head) | 0.91% | 2.94% | 1.61% | 0.782 | 0.319 | 0.181 | 1.282 |
| Coconut Dream Corridor | 0.00% | 2.49% | 0.00% | 2.815 | 0.001 | 2.904 | 5.72 |
| West Island | 0.00% | 2.48% | 1.54% | 0.621 | 0.240 | 0.089 | 0.95 |
| Atlantis | 2.71% | 2.04% | 1.57% | 0.219 | 0.303 | 0.007 | 0.529 |
| Duty-Free Shop | 3.05% | 1.50% | 2.01% | 0.601 | 0.177 | 0.225 | 1.003 |
| Nanshan Temple | 0.98% | 1.14% | 0.94% | 0.001 | 0.002 | 0 | 0.003 |
| Tropical Paradise Forest Park | 0.00% | 1.02% | 0.00% | 0.281 | 0.001 | 0.308 | 0.59 |
| Phoenix Island | 0.00% | 1.01% | 0.00% | 0.531 | 0.001 | 0.569 | 1.101 |
| Bohou Village | 2.02% | 0.00% | 1.40% | 0.816 | 0.139 | 0.582 | 1.537 |
| Museum | 0.00% | 0.00% | 1.11% | 0.038 | 0.218 | 0.638 | 0.894 |
| Da Xiaodongtian  (Big and Small Caves) | 0.00% | 0.00% | 1.06% | 0.038 | 0.651 | 1.003 | 1.692 |
| Column totals (percentage and sum of squared Aitchison distances) | 100% | 100% | 100% | 12.306 | 2.504 | 12.716 |  |

Table S5 Affective component analysis from 2013 to 2019

|  | Percentage of keywords (%) within data source | | | Contributions to distance between sources | | | Row  sum |
| --- | --- | --- | --- | --- | --- | --- | --- |
|  |  |  |  |  |  |  |  |
|  | PGC | TGC | OGC | P-T | P-O | T-O |  |
| Enjoyment | 22.138% | 15.588% | 21.925% | 0.100 | 0.002 | 0.074 | 0.176 |
| Beauty | 20.207% | 15.067% | 19.870% | 0.067 | 0.003 | 0.043 | 0.113 |
| Freedom | 14.272% | 10.082% | 6.479% | 0.098 | 0.679 | 0.261 | 1.038 |
| Romance | 10.363% | 7.037% | 15.904% | 0.124 | 0.155 | 0.558 | 0.838 |
| Nature | 12.294% | 9.887% | 13.185% | 0.034 | 0.001 | 0.048 | 0.083 |
| Free | 10.363% | 15.770% | 22.637% | 0.206 | 0.558 | 0.086 | 0.851 |
| Convenience | 10.363% | 19.768% | 0.000% | 0.463 | 0.647 | 2.205 | 3.315 |
| Queuing | 0.000% | 6.800% | 0.000% | 0.290 | 0.000 | 0.276 | 0.567 |
| Column totals (percentage and sum of squared Aitchison distances) | 100% | 100% | 100% | 1.383 | 2.046 | 3.551 |  |

Table S6 Affective component analysis from 2020 to 2022

|  | Percentage of keywords (%) within data source | | | Contributions to distance between sources | | | Row  sum |
| --- | --- | --- | --- | --- | --- | --- | --- |
|  |  |  |  |  |  |  |  |
|  | PGC | TGC | OGC | P-T | P-O | T-O |  |
| Enjoyment | 20.755% | 20.008% | 31.486% | 0.001 | 0.121 | 0.104 | 0.226 |
| Beauty | 12.264% | 15.486% | 15.297% | 0.087 | 0.023 | 0.021 | 0.131 |
| Romance | 15.330% | 14.436% | 15.475% | 0.000 | 0.004 | 0.004 | 0.007 |
| Free | 20.519% | 14.394% | 27.082% | 0.085 | 0.044 | 0.251 | 0.380 |
| Freedom | 11.792% | 13.484% | 10.660% | 0.039 | 0.029 | 0.134 | 0.201 |
| Cheap | 0.000% | 9.241% | 0.000% | 0.186 | 0.001 | 0.166 | 0.352 |
| Excitement | 10.377% | 6.763% | 0.000% | 0.134 | 0.598 | 0.166 | 0.897 |
| Queuing | 8.962% | 6.189% | 0.000% | 0.095 | 0.543 | 0.184 | 0.823 |
| Column totals (percentage and sum of squared Aitchison distances) | 100% | 100% | 100% | 0.627 | 1.241 | 1.029 |  |
