# Supplementary material for: Measuring destination image dissonance: A compositional data analysis of cross-media representations in Sanya’s Tourism
Source: PLoS One. 2026 Oct 15;21(10):e0360383. doi: 10.1371/journal.pone.0360383 (PMC13671502; doi:10.1371/journal.pone.0360383)
Supplement: Supplementary file 5 [file pone.0360383.s005.docx]

**Point-by-Point Response to Reviewer Comments**

We sincerely appreciate you taking the precious time out of your busy schedule to conduct an extremely meticulous review of our manuscript and provide highly professional and insightful comments and suggestions. Your review is not only rigorous, thorough and pinpointed—accurately identifying the shortcomings and areas for improvement in our research—but also offers us a wealth of inspiring ideas with your profound professional expertise, illuminating a clear direction for the refinement and optimization of the entire study.

Upon receiving your review comments, our team promptly held in-depth discussions on each comment one by one, carefully deliberating over every suggestion and fully integrating them into the manuscript revision process. From deepening and improving the theoretical framework, supplementing and strengthening methodological validation, to enriching and optimizing visual presentations, and further enhancing the tight connection between research results and theoretical logic, every revision embodies your professional insights. It is precisely these highly targeted and valuable suggestions that have helped us effectively address the weaknesses in our research, significantly improving the academic rigor, logical clarity and practical guidance of the entire study, while also deepening our understanding of the research topic.

We would like to extend our most sincere gratitude to you once again! Thank you for your generous sharing of professional feedback and selfless contribution of academic wisdom—every effort you have made provides crucial support for enhancing the quality of this manuscript. We sincerely hope that the revised manuscript meets the publication requirements and earnestly request your continued valuable guidance and corrections.

1. **Introduction**

**Reviewer Comment 1**

The opening paragraph is too descriptive and contains the history of TDI theory without explicitly emphasizing the research gap at the end of the section.

***Response:*** We have streamlined the redundant description of the TDI theory development history as suggested, focusing on presenting the core research gaps. After revision, excessive chronological accounts of the theory in the original introduction have been removed, with emphasis placed on two core gaps: 1) the traditional "cognitive-affective" dualistic framework ignores the spatial dimension, which is disconnected from the spatial attributes of tourism experiences in the digital age; 2) existing quantitative methods (content analysis, semantic network analysis) struggle to accurately capture the relative associative characteristics of cross-media image elements. At the end of the introduction, it is clearly stated that this study aims to address the aforementioned theoretical and methodological gaps through the "cognitive-spatial-affective" tripartite framework and Compositional Data Analysis (CoDA), making the presentation of research gaps more direct and focused.

**Reviewer Comment 2**

The logical ﬂow towards the research goals is rather abrupt — the explanation of the "cognitive–affective–spatial" concept and the transition to the use *of* *Compositional* *Data* *Analysis* *(CoDA)* are less connected to the emptiness of previous research.

***Response:*** We have strengthened the logical connections between concepts. After revision, we first elaborate on the neglect of the spatial dimension in traditional dualistic frameworks, and combined with practical scenarios such as "internet-famous check-in spots" and "City Walk", we explain the necessity of tourism geography emphasizing spatial attributes, thereby naturally justifying the rationality of the "cognitive-spatial-affective" integrated tripartite framework. Subsequently, by linking the limitations of existing quantitative methods—their inability to handle the "constant-sum constraint" of tourism image data—we introduce the core advantage of the CoDA method, which is specifically designed to analyze "proportion data of parts within a whole", and clarify its suitability as a solution for quantifying cross-media image discrepancies. This makes the transition between the theoretical framework and research method smoother.

**Reviewer Comment 3**

No *clear* *research* *question* or hypothesis is explicitly stated.

***Response:*** We have supplemented clear research objectives and implicitly embedded core research questions in the introduction. After revision, three key research objectives are clearly listed: 1) Quantitatively measure the specific degree of inconsistency in cognitive, spatial, and affective images across PGC, TGC, and OGC; 2) Examine how the COVID-19 pandemic has changed or exacerbated such image inconsistencies; 3) Derive targeted recommendations based on data to bridge the identified image gaps. These three objectives correspond to the core research questions of "what is the degree of discrepancy", "what is the impact of the pandemic", and "how to bridge the gaps" respectively, making the research orientation more clear and definite.

2. **Literature** **Review**

**Reviewer Comment 1**

The structure of the chronological sub-chapters (period 1973–2015) is historical but less analytical; it has not shown a direct relationship with the research variables (cognitive-spatial-emotional).

***Response:*** We have restructured the literature review and abandoned the purely chronological division, instead adopting three thematic modules: 1) Core dimensions of tourist destination image (focusing on the theoretical evolution and integration necessity of cognitive, spatial, and affective dimensions); 2) Multi-subject representation and cross-media image discrepancies (focusing on the characteristics of image representation by PGC, OGC, TGC and the limitations of existing research); 3) Methodological evolution and the introduction of Compositional Data Analysis (comparing the defects of traditional methods and the suitability of CoDA). Each module closely revolves around the core research variables, strengthening the relevance between the literature review and the research content.

**Reviewer Comment 2**

Some classic quotes (Echtner 1991; Gartner 1993) are not updated with post-2020 literature relevant to *big* *data* and *cross-media* *analytics*.

***Response:*** We have supplemented the latest relevant literature published after 2020. New additions include studies such as Jose et al. (2024) on the intellectual structure of destination image research in the big data era, Kirilenko et al. (2024) on cross-platform online review discrepancies, and Carolin et al. (2024) on the spatial distribution comparison of social media visual content. These additions create a connection between classic citations and recent research, update the contextual background of the study, and demonstrate an understanding of the latest developments in the field.

**Reviewer Comment 3**

The *Gaps* *in* *Representation* section mentions the gap between sources but does not afﬁrm *why* *compositional* *data* is a methodological solution.

***Response:*** We have clarified the methodological advantages of CoDA in the "Methodological Evolution" module. After revision, we have made a detailed comparison of the defects of traditional methods (content analysis, semantic network analysis) in handling the "constant-sum constraint of compositional data", and explained that tourist image data (keyword frequencies) are essentially "proportion data of parts within a whole". The CoDA method maps data from the simplex space to the real space through log-ratio transformation, which can effectively solve the "spurious correlation" problem of traditional methods. At the same time, we have supplemented the application status of CoDA in tourism research, clarifying its unique value as a tool for quantifying cross-media image discrepancies and improving the logical argumentation for methodological selection.

3. **Methodology**

**Reviewer Comment 1**

The technical explanation of CoDA is very detailed (formulas, transformations, formulas) but too mathematical without adequately explaining *why* *this* *method* *ﬁts* *tourism* *data*.

***Response:*** We have simplified the presentation of redundant mathematical formulas and strengthened the explanation of the suitability between the method and tourism data. After revision, we focus on explaining the "compositional attributes" of tourism data—keyword frequencies of image elements in various media have the characteristics of "constant-sum constraint" (sum of proportions equals 100%) and "relative importance". Traditional statistical methods cannot accurately analyze such data; however, the core advantage of CoDA lies in eliminating the impact of constant-sum constraints through centered log-ratio transformation (clr), and its Aitchison distance can accurately measure the overall discrepancy between image profiles of different media. This perfectly meets the quantitative needs of cross-media discrepancies in tourism images, making the logic of method suitability easier to understand.

**Reviewer Comment 2**

The validity and reliability of *the* *keyword* *extraction* process (dictionary, stopword, synonym list) have not been empirically tested (e.g., inter-coder or pretest).

***Response:*** We have supplemented the reliability and validity testing procedures for keyword extraction. After revision, we have detailed the "double-blind coding" verification process: two coders familiar with tourism research independently classified high-frequency words from a random sample into "cognitive-spatial-affective" dimensions. After resolving classification discrepancies through discussions, a high level of consensus was reached (Cohen's Kappa > 0.85), ensuring the content validity of the classification. At the same time, we have explained the construction and optimization processes of the custom dictionary, stopword list (integrating stopwords from Harbin Institute of Technology, Baidu, and ROST.CM), and synonym list, clarifying the rigor of the extraction process and addressing the lack of reliability and validity testing.

**Reviewer Comment 3**

The criteria for selecting 50 *common* *keywords* *are* *not* *explained* — the potential for selection bias is quite high.

***Response:*** We have clarified the selection criteria for the 50 common keywords. After revision, we have specified that: from the top 100 high-frequency words of each of the three media types (PGC, TGC, OGC), the "50 keywords with the highest comprehensive frequency" were selected. This criterion ensures that the selected keywords are the most representative and universal core image elements in cross-media scenarios, minimizing selection bias and focusing the analysis on the core components of Sanya's tourism image, thus improving the logical rationality of keyword selection.

**Reviewer Comment 4**

Spatial analysis is less equipped with visualizations (maps or heatmaps).

***Response:*** We have supplemented visualization charts for spatial analysis. （Figure5-7）

4. **Results**

**Reviewer Comment 1**

The results are presented very long and the tables are dense with numbers, causing difﬁculties in capturing conceptual meaning.

***Response:*** We have streamlined the presentation format of the results and highlighted core data. After revision, detailed original data tables (such as complete keyword frequency tables) have been moved to the supplementary materials of the paper, and only summary tables of key statistical results (e.g., core data tables of Aitchison distances in each stage) are retained in the main text. At the same time, core trends are summarized through text to avoid data redundancy. For example, in the cognitive image analysis, key conclusions such as "the cognitive gap between TGC and OGC widened by 40.6% after the pandemic" and "TGC cognition shifted from 'coastal' to 'coastal + food'" are emphasized, making the presentation of results more concise and focused.

**Reviewer Comment 2**

Narrative analysis tends to be descriptive (calling it "top ﬁve keywords") without directly relating to the theory of destination perception or tourist behavior.

***Response:*** We have strengthened the connection between result analysis and theories. After revision, descriptive analysis is deeply integrated with relevant theories: for instance, the evolution of TGC's cognitive focus is linked to the "experience economy theory" to explain the internal logic of tourists' demand shift from landscape sightseeing to sensory experiences; the spatial image discrepancies are combined with the "spatial production" theory in tourism geography to interpret the misalignment mechanism between the "representational space" of OGC and the "lived space" of TGC; the emotional image discrepancies are connected to the "service-dominant logic" to illustrate the impact of tourists' authentic emotional spectrum on value judgment. This elevates the result analysis from "phenomenon description" to the level of "theoretical interpretation".

**Reviewer Comment 3**

No signiﬁcance tests or robustness were tested between periods.

***Response:*** We have supplemented cross-period significance tests. After revision, Dirichlet regression analysis has been added to verify the significance of cross-media image discrepancies before and after the pandemic, considering the constant-sum constraint characteristics of compositional data. The results show that there are significant differences in the proportion of core components in the cognitive and spatial dimensions before and after the pandemic (p<0.01), and significant differences in the proportion of functional emotions in the affective dimension (p<0.05), excluding the impact of random fluctuations on the results. At the same time, the robustness of core conclusions is verified through a comparison of trends in two stages (2013–2019 vs. 2020–2022), making the research results more reliable.

**Reviewer Comment 4**

Visualizations (graphs, Aitchison distance charts) are not available, although it is important to clarify cross-media dissonance.

***Response:*** We have enriched the visualization charts in the results section. After revision, various core charts have been added: 1) Aitchison distance radar charts (Figures 3, 6, 9) to show the degree of discrepancy among the three media in cognitive, spatial, and affective dimensions; 2) Comparison charts of keyword distance contributions (Figures2 , 5, 8) to demonstrate the impact of core elements on cross-media discrepancies; 3) Bar charts of media pair discrepancy analysis (Figures 4, 7, 10) to intuitively present the trend of discrepancy changes in the two stages. These charts clearly illustrate the core characteristics of cross-media image dissonance, making the results more readable and persuasive.

5. **Conclusions and Implications**

**Reviewer Comment 1**

The conclusions afﬁrm the existence of an "institutional-tourist narrative misalignment" but do not elaborate on its scientiﬁc contribution to the theory of TDI or tourism communication.

***Response:*** We have clarified and detailed the theoretical contributions of the study. After revision, the contributions are systematically elaborated from three aspects: 1) Constructing and verifying the "cognitive-spatial-affective" integrated tripartite framework, responding to the call of tourism geography and promoting the development of TDI theory from dualistic opposition to tripartite integration; 2) Systematically applying the CoDA method to the quantification of cross-media image discrepancies for the first time, advancing the research on image consistency from the "content level" to the "structural relationship level", and providing a new measurement dimension and theoretical language for the classic "projected-perceived" framework; 3) Revealing the dual-track evolution mechanism of "policy response-experience lag" in image evolution under crisis scenarios, supplementing contextual evidence for crisis communication and image restoration theories, and improving the depth of elaboration on theoretical contributions.

**Reviewer Comment 2**

The policy implications are still general, not showing how destination governments can use the results of the CoDA operationally.

***Response:*** We have refined the policy implications into actionable practical solutions. After revision, three specific recommendations are proposed: 1) Establish a "destination image consistency monitoring dashboard"—suggesting that destination management organizations (DMOs) adopt a quarterly CoDA monitoring mechanism, using Aitchison distance as a core indicator and setting early warning thresholds to realize dynamic management of image discrepancies; 2) Implement "differentiated precision content marketing"—designing scenario-based integrated products (e.g., "sailing at sea + seeking fresh seafood at fish markets") and optimizing spatial promotion strategies to address discrepancies in cognitive and spatial dimensions; 3) Conduct "emotional touchpoint management and sensory marketing"—transforming emotional keywords such as "convenience", "queuing", and "excitement" in TGC into specific service optimization measures (e.g., time-slot reservations, night light festivals). All recommendations are clearly combined with the results of CoDA analysis and have direct operability.

**Reviewer Comment 3**

There are no limitations and future research agendas.

***Response:*** We have added a new section titled "Research Limitations and Future Directions" (Section 5.4 of the paper). After revision, three key research limitations have been clearly identified: 1) Limitation in data sources (Tourist Generated Content (TGC) is solely sourced from Ctrip, excluding visual platforms such as Xiaohongshu and Douyin); 2) Methodological limitation (Compositional Data Analysis (CoDA) excels at quantifying discrepancies but is unable to reveal causal relationships); 3) Limitation in the theoretical framework (failure to incorporate other stakeholders, such as local residents and tourism practitioners). Corresponding future research directions have also been proposed: 1) Integration of multi-modal data (incorporating image, short video, and audio data); 2) Testing of causal models (constructing a structural equation model of "image dissonance → tourist expectations → satisfaction"); 3) Cross-cultural and cross-regional comparisons (applying the framework to different types of tourist destinations), which enhances the completeness and forward-looking nature of the study.
