# Supplementary material for: Measuring destination image dissonance: A compositional data analysis of cross-media representations in Sanya’s Tourism
Source: PLoS One. 2026 Oct 15;21(10):e0360383. doi: 10.1371/journal.pone.0360383 (PMC13671502; doi:10.1371/journal.pone.0360383)
Supplement: Supplementary file 6 [file pone.0360383.s006.docx]

**Point-by-Point Response to Reviewer Comments**

Dear Reviewer,

We sincerely appreciate your thorough and insightful review of our manuscript. Your constructive comments have been invaluable in helping us to significantly strengthen the theoretical rigor, clarity, and overall structure of the paper. We have carefully considered and addressed each of your points. Our point-by-point responses and the corresponding revisions are detailed below.

**Comment A) *Introduction* *- Although the gap was improved, it is not theoretical sound in terms of justification. In some parts of your text, you have too many long sentences. For example, the paragraph about the aim. Consider making the sentences shorter and more direct.***

**Response:**We are very grateful for this critical observation. In response, we have substantially revised the Introduction section to enhance its theoretical foundation and improve readability.

1. Strengthening Theoretical Justification: To provide a more solid theoretical grounding, we have now explicitly cited key literature that underpins the cognitive-affective model (Baloglu & McCleary, 1999), justifies the inclusion of the spatial dimension (Lynch, 1960), and supports the use of Compositional Data Analysis (Aitchison, 1986; Coenders & Ferrer-Rosell, 2020). This strengthens the rationale for our tripartite “cognitive-spatial-affective” framework and methodological choice.
2. Simplifying Sentence Structure: As suggested, we have broken down lengthy and complex sentences into shorter, more direct statements. This is particularly evident in the paragraph stating the research aims, which has been rewritten into a concise introductory sentence followed by a clearly itemized list of objectives (see the second-to-last paragraph of the revised Introduction). This revision enhances clarity and eases comprehension.

**Comment B) *Literature review - Just call it “Tourism destination image”, since some core studies were neglected. Despite some improvements, this section needs improvements, in terms of destination image (DI) formation, and DI implications.***

**Response:** Thank you for this suggestion, which prompted us to refine the focus of our literature review.

Regarding the title, we have restructured the section to better align with the core theme. We believe this provides a more precise roadmap for our specific study design than a generic title. However, we are happy to adopt your suggested title if you deem it more appropriate upon review of our revisions.

Concerning the content, we understand your call for a broader discussion of DI formation and implications. Our literature review is intentionally structured to build a direct foundation for our study's core focus: the measurement of differences across sources and dimensions, rather than a comprehensive review of all TDI facets.

**Comment C) *I miss a specific discussion section.***

Response: We completely agree with this essential structural suggestion. As recommended, we have added a dedicated “Discussion” section as the new Chapter 5 (subsequent chapter numbers have been updated accordingly). In this section, we:

Interpret our key findings (e.g., the “institutional narrative alliance” vs. “tourist experiential narrative”) by engaging with relevant theories from tourism geography and media studies.

Explain how the COVID-19 pandemic acted as a “catalyst” amplifying existing dissonances, discussing underlying mechanisms like institutional path dependence.

Reflect on the methodological contributions and limitations of applying the CoDA framework to tourism image analysis.

Elucidate the broader theoretical and practical implications of our results.

**Comment D) *Conclusion - as is, it reads more as a discussion not a real conclusion. Revise this section.***

**Response:** Thank you for this correction. We have thoroughly revised the Conclusion section (now Section 6.1) to make it more concise and summative. We have removed explanatory and discursive content, which has been moved to the new Discussion chapter. The conclusion now concisely restates the three core findings of the study in direct, declarative statements, providing a clear answer to the original research questions. It ends with a brief sentence highlighting the main takeaway for destination image management, ensuring the section serves as a true summary of the paper’s outcomes.

We believe these revisions have comprehensively addressed your valuable comments and greatly improved the manuscript. Thank you again for your time and guidance. Wishing you and your family a happy New Year!

Sincerely,

The Authors
