# Supplementary material for: Measuring destination image dissonance: A compositional data analysis of cross-media representations in Sanya’s Tourism
Source: PLoS One. 2026 Oct 15;21(10):e0360383. doi: 10.1371/journal.pone.0360383 (PMC13671502; doi:10.1371/journal.pone.0360383)
Supplement: Supplementary file 7 [file pone.0360383.s007.docx]

**Point-by-Point Response to Reviewer Comments**

Dear Reviewer,

We sincerely appreciate the valuable comments and suggestions you have provided, which have greatly helped us improve the quality of our manuscript. We have carefully addressed all the questions raised by the reviewers, and the specific revisions are as follows:

**Response to Reviewer 1**

We are very grateful for your comments on the data availability of this manuscript. We fully agree with your suggestions and have made all the necessary revisions as required to comply with the data policies of PLOS ONE. A detailed response to your specific suggestions is provided below:

Response to Point 4 (Data Availability)

We have uploaded all processed datasets and the corresponding analysis codes to a publicly accessible repository (GitHub, URL: <https://github.com/x9w259pp86-stack/datesets-for-Plos-one.git> ). All data and codes are complete and available for free access, download and reuse by other researchers, ensuring the reproducibility of the study results.

**Response to Reviewer 2**

We highly appreciate your recognition of the improvements made to this manuscript as well as your valuable final comments. We have carefully noted your suggestion to avoid using numbered bullet points in the Discussion and Conclusion sections and have made the corresponding revisions accordingly.

Specifically, we have removed all numbered bullet points from the "Discussion" and "Conclusion" sections of the manuscript and reorganized the content of these two sections into coherent paragraph-style descriptions.

In summary, we have carefully addressed all the comments raised by the two reviewers. All revisions are based on the original research content and academic norms, aiming to further enhance the rigor and completeness of the manuscript. We sincerely hope that the revised manuscript meets the publication requirements of PLOS ONE. We would like to express our sincere gratitude again for your careful review and valuable suggestions.
