# Supplementary material for: Measuring destination image dissonance: A compositional data analysis of cross-media representations in Sanya’s Tourism
Source: PLoS One. 2026 Oct 15;21(10):e0360383. doi: 10.1371/journal.pone.0360383 (PMC13671502; doi:10.1371/journal.pone.0360383)
Supplement: Supplementary file 8 [file pone.0360383.s009.docx]

**Point-by-Point Response to Reviewer Comments**

Dear Reviewer,

We sincerely appreciate your thorough and insightful review of our manuscript. Your constructive comments have been invaluable in helping us to significantly strengthen the theoretical rigor, clarity, and overall structure of the paper. We have carefully considered and addressed each of your points. Our point-by-point responses and the corresponding revisions are detailed below.

**Comment 1) *First, the manuscript should resolve the inconsistency in the data-availability information: the response letter states that all processed datasets and code have been uploaded to a public GitHub repository, yet the data-availability statement still says the authors are unsure how to create a URL and are willing to share data by another method. These statements should be fully aligned and the repository link should be clearly presented in the manuscript.***

**Response:** We sincerely appreciate this careful and important comment. We have completely unified and standardized all statements related to data availability in both the manuscript and the response letter to ensure full consistency. All processed datasets and the corresponding analysis codes in this study have been uploaded to a publicly accessible GitHub repository, and the URL has been clearly labeled in the “Data Availability” section of the manuscript.

The public repository is available at:

<https://github.com/x9w259pp86-stack/datesets-for-Plos-one.git>

All data and codes are complete, freely accessible, downloadable, and reusable by other researchers, which fully ensures the reproducibility of the study results.

**Comment 2) *Second, the methods section would benefit from a clearer explanation of how the 50 keywords were finalized from the top-100 lists and how the “core components” used in the Dirichlet regression were selected, since the current description is still somewhat concise relative to the importance of these analytic decisions.***

Response:

Thank you for this constructive and professional suggestion. We have **substantially supplemented and refined the Method section** with detailed, rigorous, and replicable descriptions for both key analytical procedures:

1. We added a c**omplete and transparent procedure** for selecting the 50 core keywords from the top‑100 high-frequency word lists of PGC, TGC, and OGC, including the processes of extraction, merging, deduplication, cumulative frequency ranking, and final selection.
2. We added a **comprehensive and logical explanation of the variable selection logic for Dirichlet regression**, including two rigorous selection criteria (high contribution to Aitchison distance and theoretical importance in the three-dimensional destination image framework) and the selection of reference variables for log-ratio transformation.

The revised content ensures methodological transparency and matches the academic importance of these analytical decisions.

**Comment 3) *Third, the manuscript needs careful technical editing: there is terminology inconsistency in the Discussion where PGC is defined as “Professional Generated Content” rather than “Press Generated Content,” Table 3 under the affective-image section is mislabeled as “Spatial component analysis,” and the limitations section begins with an incomplete sentence (“This may have resulted…”).***

**Response:** We highly appreciate your meticulous review. We have comprehensively checked and revised all technical inconsistencies, labeling errors, and incomplete sentences throughout the manuscript:

1. Terminology consistency: We have unified the full name of PGC as Press Generated Content.
2. Table label correction: We have corrected the mislabeled title of Table 3 from “Spatial Component Analysis” to Affective Component Analysis.
3. Incomplete sentence revision: We have revised the incomplete and ungrammatical opening sentence in the “Limitations” section (originally “This may have resulted…”) to ensure grammatical completeness, fluency, and academic accuracy.

All revisions have been double‑checked to ensure clarity, standardization, and consistency.

We believe these revisions have comprehensively addressed your valuable comments and greatly improved the manuscript. Thank you again for your time and guidance.

Sincerely,

The Authors
