# Supplementary material for: Measuring destination image dissonance: A compositional data analysis of cross-media representations in Sanya’s Tourism
Source: PLoS One. 2026 Oct 15;21(10):e0360383. doi: 10.1371/journal.pone.0360383 (PMC13671502; doi:10.1371/journal.pone.0360383)
Supplement: Supplementary file 9 [file pone.0360383.s011.docx]

**Point-by-Point Response to Reviewer Comments**

Dear Reviewer Bacon-Shone,,

We sincerely appreciate your rigorous, thorough and constructive remarks spanning empirical design, data transparency, statistical methodology, variable definition and result interpretation. Every issue you raised has been fully resolved via targeted manuscript revisions, supplementary open dataset uploads and expanded methodological elaborations. We provide a detailed point-by-point response to each of your comments as follows.

**Comment 1 & Comment 8**

**Reviewer’s Comment**

The abstract refers to a time lag, but the analysis fails to include the temporal dimension properly – it is impossible in this analysis to distinguish time trends from COVID impacts, making all the conclusions of COVID impact impossible to verify. This can be addressed by using yearly data, or at the very least dividing the pre-COVID period into multiple time periods. It would also greatly strengthen the analysis to add a post-COVID period, to see whether the disparities reduce.

Unsupported claims (see line 352) – as mentioned above, this analysis can NOT distinguish time trends from COVID impacts.

***Response:***

We fully agree with the reviewer that the two-stage comparative design (2013–2019 vs. 2020–2022) cannot statistically disentangle long-term secular trends from pandemic-specific shocks, meaning causal claims about COVID-19 effects are not empirically justified by our research setup. We have carried out four rounds of manuscript-wide revisions to resolve this issue:

1. All causal wording attributing image dissonance to the pandemic (e.g., “exacerbated”, “caused”) has been eliminated. All relevant statements are rewritten as neutral descriptive temporal comparisons, such as “the narrative divergence widened substantially in the 2020–2022 phase relative to the pre-pandemic period” and “structural misalignment appears more prominent during the pandemic-affected stage”. All findings are now presented as descriptive temporal contrasts without causal attribution.
2. The unsupported statement regarding a “3–5 year time lag” has been completely removed, as this numerical estimate cannot be derived from our dataset.
3. Supplementary methodological clarification is added at the end of Section 3.3 Compositional Data Analysis. We explicitly state that the two-period grouping only serves to describe stage-level structural discrepancies and cannot support formal significance testing or causal inference. All Aitchison distance values are interpreted merely as descriptive indicators of compositional dissimilarity.
4. A dedicated paragraph expanding on this temporal limitation is added to Section 6.4 Limitations, elaborating on how coarse temporal granularity restricts causal interpretation of cross-stage differences.

We acknowledge that annual subdivision or extended post-pandemic data would improve causal identification, yet such data expansion lies beyond the scope of the current study. We have listed annual and post-COVID longitudinal analysis as a clear direction for future research in the discussion section.

**Comment 2**

**Reviewer’s Comment**

The claim that the data is all available in gitlab is simply untrue. There is no labelling of the 50 words, in particular, as to which are cognitive, spatial and affective, let alone which link to the specific elements referenced in the analysis. Tourism, hotels, coastal food are referenced in the abstract without any connection to the data set provided! The dataset is already converted to relative frequencies and zeros removed, making it impossible to understand (for example), how the zero replacement affects the results. The dataset must include raw frequencies with full labelling provided.

***Response***

We apologize for the incomplete and disorganized dataset previously uploaded. We have fully resolved this transparency requirement by re-uploading complete supplementary datasets to the open repository:

<https://github.com/x9w259pp86-stack/datesets-for-Plos-one/blob/main/datasets-updates.zip>

The updated repository now contains the following complete materials to meet the reviewer’s requirements:

1. Raw absolute frequency matrices for all three media sources (PGC/TGC/OGC), retaining original zero-count entries without prior deletion or transformation;
2. A full classification label sheet for the 50 core keywords, clearly marking each term’s corresponding dimension (cognitive / spatial / affective), with direct cross-references to all thematic elements mentioned in the abstract and main text (tourism, hotels, coastal food, duty-free shops, Coconut Dream Corridor, etc.);
3. Intermediate datasets including unprocessed candidate keyword pools, pre-zero-imputation raw tables, and post-CLR transformation proportional data, allowing readers to trace the full impact of Bayesian zero replacement on final analytical outputs.

All data files are fully annotated with readme documents to ensure reproducibility of all keyword screening and CoDA procedures.

**Comment 3**

**Reviewer’s Comment**

Compositional data analysis (CoDA) (lines 130 onwards) is one way, not the only way to examine relative frequencies and correspondence analysis (CA) has a very specific advantage over CoDA when starting with frequency data in that it accounts for the count variability (i.e. for Poisson count data, variance is proportional to the mean) and avoids any need to make adjustments for zeros. This should be referenced as an alternative which avoids the problems of zeros and yet also provides a distance metric.

***Response***

We greatly appreciate this critical and constructive comment regarding alternative analytical approaches for keyword frequency data in tourism research. While we have adopted standard Bayesian multiplicative zero replacement to resolve zero-count obstacles within compositional data analysis (CoDA), we fully recognize that correspondence analysis (CA) is a widely used alternative suitable for raw absolute frequency matrices.

As the reviewer highlighted, CA exhibits an inherent strength for Poisson-distributed count data, as it requires no preliminary zero-imputation procedure to conduct subsequent analysis. Nevertheless, there exists an essential distinction in the core analytical focus between CA and CoDA that underpins our method selection. We have supplemented authoritative literature on correspondence analysis in the revised manuscript, systematically compared the strengths and limitations of the two techniques, and elaborated on why CoDA is more aligned with our research purpose:

This study centers on cross-media dissonance in the proportional composition of destination image elements, rather than disparities in absolute keyword occurrence counts. All media text data naturally follow the unit-sum closed constraint, where the relative weight of spatial, cognitive and affective keywords carries substantive theoretical meaning for measuring image imbalance. Centered log-ratio (CLR) transformation and the resulting Aitchison distance are dedicated compositional metrics designed to quantify divergence in proportional structures under closed-sum constraints. In contrast, CA mainly identifies differences in raw count distributions based on chi-square distance, which cannot independently extract structural deviations of proportional compositions. For this reason, CoDA is prioritized over CA in the present research, with Bayesian multiplicative zero replacement serving only as a standardized preprocessing step to eliminate computational barriers caused by missing word counts.

**Comment 4**

**Reviewer’s Comment**

The paper confuses frequencies and relative frequencies in several places (see line 125) – relative frequencies add to 1 and hence have the compositional constraint, but this is not true for frequencies.

***Response***

We sincerely appreciate the reviewer for identifying the confusion between absolute frequencies and proportional values throughout the manuscript. We fully acknowledge that unqualified use of the term “frequency” created ambiguous terminology and blurred the mathematical distinction between raw absolute counts and transformed relative proportions.

We have implemented targeted revisions across all relevant sections:

1. Section 2.3: Rewrote the problematic sentence that incorrectly treated raw keyword counts as compositional data. The revised text clarifies that only data converted into proportions follow the constant-sum constraint, whereas raw absolute frequencies do not carry this property.
2. Section 3.3: Added the modifier “absolute” to all unqualified “frequency” references, inserted a fixed total word volume constraint to correct the logical misunderstanding of how absolute counts interact with proportions, and explicitly stated that the constant-sum constraint exclusively applies to proportional data.
3. Unified analytical matrix subsection: Supplemented “absolute” to all cumulative and overall frequency descriptors to clarify that keyword screening was based on raw absolute counts rather than proportions.
4. Handling of Zero Values subsection: Revised “zero frequency values” to “zero absolute frequency” to specify that missing entries refer to raw count zeros, not proportional zeros.
5. Calculation of Aitchison Distance subsection: Added brief wording to anchor the constant-sum constraint to proportional data for consistent terminology.
6. Section 4. Results: Reframed vague references to “keyword differences” as “structural differences in keyword proportions” to make clear that all CoDA comparisons target proportional structures rather than raw absolute counts.

Throughout the revised manuscript, we strictly separate two core concepts: raw absolute frequency (the raw count of keyword occurrences) and proportion/relative frequency (standardized values summing to one with a constant-sum constraint). All statistical operations including zero replacement, centred log-ratio transformation, Aitchison distance calculation are clearly linked to proportional datasets, eliminating cross-concept confusion entirely.

**Comment 5**

**Reviewer’s Comment**

Data sources – there is no justification provided for the specific 3 sources chosen (e.g. what proportion of travellers use Ctrip?)

***Response***

We sincerely appreciate the reviewer’s constructive suggestion regarding the adequacy of data source justification. Corresponding revisions have been fully supplemented in Section 3.1, as detailed below:

1. At the opening paragraph of Section 3.1 Data Sources and Collection, we added theoretical rationale for selecting three complementary media categories (PGC, TGC, OGC). Combined analysis of official institutional narratives, authoritative media narratives and spontaneous tourist experiential narratives overcomes the one-sided defects of dual-source comparison widely adopted in existing tourism research, which clarifies why we adopt three distinct data sources.
2. In the subsection "Tourist-Perceived Image Data", we cite the official 2025 report released by QuestMobile with an accessible report link attached. These quantitative market share data directly address the reviewer’s question regarding traveller coverage on the Ctrip platform and fully demonstrate the representativeness of the Ctrip travelogue samples.

The above revisions systematically explain the rationality of the triple-source framework and provide empirical quantitative evidence for the representativeness of Ctrip TGC data, fully addressing all concerns raised in this comment.

**Comment 6**

**Reviewer’s Comment**

The paper makes the claim (line 283) that the criterion ensures the elements are the most prevalent, which is true, but fails to address whether 50 key words are sufficient, for example, what proportion of all key word references are included in the 50 key words selected?

***Response***

We appreciate the reviewer’s important reminder regarding the lack of quantitative evidence to verify the sufficiency of the 50 core keywords. Corresponding supplementary calculations and explanations have been added to the subsection Construction of a unified analytical matrix.

We supplemented the explicit coverage rate formula in the text:

Coverage rate = Cumulative absolute frequency of the top 50 screened core keywords ÷ Total cumulative absolute frequency of all unique candidate keywords merged from three media sources.

We calculated coverage rates separately for the two research periods:

- Pre-pandemic stage (2013–2019): 78.22%
- Pandemic impact and recovery stage (2020–2022): 79.23%

Following the word set selection criteria proposed by Liu et al. (2016), a cumulative word frequency coverage of 70% acts as the critical cutoff separating informative high- and medium-frequency terms from trivial long-tail low-frequency words. The two coverage rates calculated in this study both surpass the 70% threshold and fall within the optimal range of 70%–80%. This demonstrates that the 50 screened core keywords sufficiently capture the dominant semantic features of textual data on Sanya’s tourism and are appropriate for subsequent compositional data analysis.

**Comment 7**

**Reviewer’s Comment**

Formulas 2 and 3 are both incomplete – what is si and what are the summation limits? Formula 3 is clearly faulty.

**Response**

We sincerely appreciate the reviewer for identifying multiple flaws in the self-defined formulas for zero-value processing in the original manuscript, such as undefined variables, missing summation ranges, and logical errors arising from circular formula construction. Although these formulas were adapted from existing literature, we have double-checked them and fully acknowledge all the above mathematical defects.

During the actual data processing, we never utilised these flawed custom formulas. All preprocessing for zero values was implemented via the standard Bayesian-multiplicative zero replacement algorithm built into the R package zCompositions, which strictly follows the authoritative algorithm framework proposed by Martín‑Fernández et al. (2015). The full executable R code for zero replacement is attached in Appendix for reproducibility.

To eliminate misleading mathematical expressions and avoid confusion for readers, we have removed all formulas throughout the revised manuscript. This includes not only the problematic zero-replacement formulas but also the transformation and distance calculation formulas that were retained previously. Every mathematical operation — from zero-value substitution and centred log-ratio transformation to Aitchison distance computation — is fully described verbally and supported by relevant citations. No mathematical formulas or references to formula numbers appear anywhere in the revised text. These revisions comprehensively address all of the reviewer’s concerns regarding incomplete and structurally erroneous formulas.

### **Appendix : Bayesian Multiplicative Zero Replacement Code**

library(readxl)

try <- read_excel("文件路径")

View(try)

cmultRepl(try, label = 0,method = c("GBM","SQ","BL","CZM","user"), output = c("prop","p-counts"),frac = 0.65, threshold = 0.5, adjust = TRUE, t = NULL, s = NULL,z.warning = 0.8, suppress.print = FALSE)

df<-cmultRepl(space, label = 0,method = c("GBM","SQ","BL","CZM","user"), output = c("prop","p-counts"),frac = 0.65, threshold = 0.5, adjust = TRUE, t = NULL, s = NULL,z.warning = 0.8, suppress.print = FALSE)

write.csv(df,"文件路径df.csv")

**Comment 9**

**Reviewer’s Comment**

The Dirichlet regression (lines 470 to 507) is clearly faulty – it ignores that Dirichlet models require conditional independence (see the Aitchison reference) and regression requires independent errors. Further, there are really only 2 data points as there are only 2 time periods, so I have no idea what analysis was done (and it is impossible to tell, given the dataset provided). The discussion linked to this analysis is hence clearly faulty as well.

***Response***

We highly appreciate the reviewer’s rigorous inspection of our Dirichlet regression analysis. We fully recognize that our two aggregated time periods fail to satisfy the core model assumptions of Dirichlet regression, including conditional independence and independent error terms. With merely two temporal groups, the sample size is insufficient to conduct valid significance testing via regression models. All interpretations derived from the Dirichlet regression outputs are therefore unreliable.

To resolve this fundamental methodological flaw, we have completely removed all Dirichlet regression content from the revised manuscript, including corresponding result tables, p-value outputs, and all associated discussion paragraphs. The Aitchison distance remains the sole quantitative metric for reporting cross-media compositional divergence, and all analytical results are presented as purely descriptive observations without any statistical significance claims.

This major revision eliminates invalid inferential analysis and aligns all empirical conclusions strictly with what our limited two-period dataset can reliably support, substantially improving the methodological rigor of this paper. We are grateful to the reviewer for detecting this critical error.

All revisions throughout the manuscript have been carefully double-checked to maintain consistent terminology, clear logical presentation and standardized statistical descriptions.

We firmly believe that the above revisions have fully addressed all your valuable suggestions and substantially strengthened the methodological rigor and reproducibility of this work. We are truly grateful for your precious time and insightful guidance that greatly helped us improve this paper.

Sincerely,

The Authors
