# Supplementary material for: Measuring destination image dissonance: A compositional data analysis of cross-media representations in Sanya’s Tourism
Source: PLoS One. 2026 Oct 15;21(10):e0360383. doi: 10.1371/journal.pone.0360383 (PMC13671502; doi:10.1371/journal.pone.0360383)
Supplement: Supplementary file 10 [file pone.0360383.s012.docx]

**Point-by-Point Response to Reviewer Comments**

Dear Prof. John Bacon‑Shone,

We are sincerely grateful for your thorough, constructive and insightful comments on our manuscript. Your careful feedback has greatly helped us improve the conceptual rigor, methodological clarity, and reproducibility of this work. We have addressed each of your concerns point‑by‑point below. All corresponding revisions have been implemented within the revised manuscript; modified text passages are quoted where applicable.

***Comment R5-1. In the Abstract, the abbreviations PGC, TGC, and OGC should be spelled out in full at their first occurrence.***

***Response***

We thank the reviewer for this reminder. In the revised Abstract, we have now spelled out all three abbreviations at their first occurrence: “Press Generated Content (PGC)”, “Tourist Generated Content (TGC)”, and “Official Generated Content (OGC)”. The revised sentence reads:

“This study examines Sanya’s tourism image disparities across cognitive, spatial, and affective dimensions in Press Generated Content (PGC), Tourist Generated Content (TGC), and Official Generated Content (OGC) (2013–2022), comparing pre-pandemic (2013–2019) and COVID-19 (2020–2022) periods.”

***Comment R5-2. The statement in the previous response that "All data files are fully annotated with readme documents to ensure reproducibility of all keyword screening and CoDA procedures" is inaccurate. In reality, there is no annotated readme file, and the data labels are only in Chinese (without an English version), making it impossible to match them against the manuscript text. This must be corrected.***

***Response***

We thank the reviewer for pointing out this inaccurate statement in our prior response. We sincerely apologize for this misrepresentation: no standalone readme document was prepared for the deposited dataset.

The original dataset and processing scripts originate from a Chinese‑language research workflow; hence variable labels and comments within the raw code are natively in Chinese. We have taken practical steps to improve cross‑referencing between dataset contents and the English manuscript text.

We have revised the deposited data files to add bilingual (Chinese‑English) labels for all core keyword entries and dimension categories (cognitive, spatial, affective) directly within the data tables. Each keyword is now paired with its English equivalent used in the manuscript, enabling reviewers and readers to match dataset entries with the descriptions presented in the article.

The complete executable processing R scripts have been uploaded to our public repository. Readers can trace all steps including keyword filtering, zero‑replacement, and Compositional Data Analysis procedures directly from the provided code.

Repository link:

<https://github.com/x9w259pp86-stack/datesets-for-Plos-one/blob/main/datasets-updates2026-0901.zip>

***Comment R5-3. The statement in the previous response that "CA mainly identifies differences in raw count distributions based on chi-square distance, which cannot independently extract structural deviations of proportional compositions" is incorrect. No CA-related literature is cited, suggesting unfamiliarity with existing CA research. The reviewer recommends the open-access paper at [https://econ-papers.upf.edu/papers/1857.pdf], which demonstrates that CA can serve as an alternative to CoDA.***

***Response***

We sincerely thank the reviewer for this valuable comment and for pointing out relevant alternative analytical workflows based on correspondence analysis.

Unfortunately, the direct working‑paper link provided by the reviewer was inaccessible. Following the reviewer’s note that this work has been released in a revised peer‑reviewed journal version, we have identified the formally published article by Greenacre (2024). This reference has been added to the reference list of our revised manuscript.

We recognise that our original statement was oversimplified and overly absolute, dismissing the analytical potential of CA‑based pipelines for compositional‑type research questions. We have revised Section 3.3 of the manuscript to acknowledge that CA‑based approaches can represent feasible alternatives for compositional‑like frequency‑based datasets.

Our choice to adopt the CoDA framework with centered log‑ratio transformation and Aitchison distance is driven by our specific research goal: measuring compositional structural divergence under the strict closed constant‑sum constraint for our keyword proportions. This selection does not imply that CA‑based methods are inherently invalid.

The revised text in Section 3.3 reads:

Correspondence Analysis (CA) operates on raw count contingency tables using chi-square distance. Published literature has demonstrated that CA-based workflows can serve as feasible alternatives for compositional-like frequency-based datasets [53]. Nevertheless, standard CA does not explicitly address the closed constant-sum constraint central to Aitchison-style compositional-data analysis. Given our core objective of quantifying structural divergence within closed proportional keyword compositions, we adopt the centered log-ratio transformation and Aitchison distance within the CoDA framework in the present study.

***Comment R5-4. The claim that "70% cumulative word frequency coverage acts as the critical cutoff separating informative high- and medium-frequency terms from trivial long-tail low-frequency words" is incorrect. The appropriate threshold is dataset-dependent, and the best way to justify a threshold is to present cumulative coverage curves. The authors should add a figure demonstrating that the threshold is reasonable for these three datasets.***

***Response***

We thank the reviewer for pointing out this important conceptual caveat. We fully agree that a cumulative word‑frequency coverage value of 70 % should not be treated as a universal critical cutoff, since suitable thresholds for keyword selection are inherently dataset‑dependent. Accordingly, we have removed this absolute claim from the revised manuscript. We now interpret the 70 %‑80 % range reported in Liu et al. (2016) only as a heuristic empirical reference for co‑word analysis, rather than a definitive fixed criterion.

To empirically justify our keyword‑selection procedure, we have added a new figure (Figure 2) showing cumulative word‑frequency‑coverage curves across all six dataset subsets (PGC, TGC, OGC for both the pre‑pandemic 2013‑2019 phase and the pandemic‑impacted 2020‑2022 phase. Curves are plotted based on each subset’s extracted top‑100 candidate keywords. It should be noted that our final 50 keywords used for compositional‑data analysis were selected from a merged global candidate pool aggregated across three media sources, sorted by summed absolute frequency across PGC, TGC and OGC, rather than directly truncating rank 1‑50 from each individual dataset subset, a horizontal dashed line indicates the heuristic 70 % reference level from prior literature. The curves visually illustrate the typical long‑tail distribution of word frequencies within each media‑period subset.

We would like to further clarify the rationality of selecting 50 keywords, and distinguish two different statistical scopes to avoid potential confusion.

First, Figure 2 visualizes cumulative word‑frequency coverage within each individual media‑period subset’s top‑100 candidate pool, which demonstrates word‑frequency long‑tail characteristics. In contrast, the reported coverage values of 78.22 % (Phase 1) and 79.23 % (Phase 2) are calculated on the merged, deduplicated global candidate pool aggregated from PGC, TGC and OGC. These two metrics are computed over different keyword populations and are therefore not expected to match numerically.

Second, we acknowledge that if we expanded the per‑media extraction threshold to top‑200 or top‑300 words, the size of the merged global candidate pool would increase, and the cumulative coverage of the global top‑50 keywords would indeed be diluted. This is exactly why we adopted top‑100 as the per‑media extraction boundary. Extracting substantially more keywords per source would introduce numerous sporadic low‑frequency noise terms with marginal semantic contribution to core destination‑image features. These extra terms would lower the global coverage metric without adding meaningful analytical value.

Third, the 70 %‑80 % heuristic reference range proposed by Liu et al. (2016) operates at this merged‑global‑candidate‑pool level. Our two observed values (78.22 % and 79.23 %) fall well within this empirical interval.

Beyond frequency‑based coverage, semantic interpretability forms another essential constraint for our study. Our analytical framework requires manual classification of each keyword into cognitive, spatial, or affective dimensions. A substantially larger keyword set would bring excessive low‑signal sporadic terms and impair the reliability of dimensional categorization. Conversely, retaining too few keywords would omit essential destination‑image dimensions. Therefore, selecting 50 keywords represents a practical balance between adequate frequency coverage and manageable semantic‑dimensional interpretability for our core goal of measuring structural destination‑image dissonance. Our analysis targets dominant semantic elements of destination images and does not aim to exhaust all infrequent long‑tail vocabulary.

The revised text within Section 3.3 “Construction of a unified analytical matrix” reads:

Liu et al. (2016) reported a heuristic empirical reference range of 70%‑80% cumulative word‑frequency coverage for keyword filtering in co‑word analysis. Importantly, such coverage values are dataset‑specific and should not be treated as a universal fixed cutoff. To visually justify our keyword‑selection procedure, we provide cumulative word‑frequency‑coverage curves for each of the six dataset subsets (PGC, TGC, OGC across both time periods) based on their respective top‑100 candidate keywords (Figure 2). The curves illustrate how word frequency accumulates within each individual media‑period subset. Our selected top‑50 global keywords fall within a stable high‑coverage region and adequately represent dominant semantic features of Sanya‑tourism textual data, supporting the suitability of this keyword subset for subsequent compositional‑data analysis. In addition to frequency‑coverage metrics, semantic interpretability for cognitive‑spatial‑affective classification was also considered when determining the size of the final keyword subset. An excessively large keyword pool would introduce many low‑signal sporadic terms and compromise the reliability of dimensional classification.

We once again thank Prof. Bacon‑Shone for your rigorous evaluation and valuable suggestions, which have substantially strengthened this manuscript. We hope the revised version is now suitable for further consideration for publication.

Sincerely,

The Authors
