# Supplementary material for: Measuring destination image dissonance: A compositional data analysis of cross-media representations in Sanya’s Tourism
Source: PLoS One. 2026 Oct 15;21(10):e0360383. doi: 10.1371/journal.pone.0360383 (PMC13671502; doi:10.1371/journal.pone.0360383)
Supplement: Peer review history

# Peer review: Decision Letter 0

**DOI:** 10.1371/journal.pone.0360383.r001

**Authors:** Jason Morgan

17 Oct 2025

Dear Dr. Li,

Please submit your revised manuscript by Nov 30 2025 11:59PM. If you will need more time than this to complete your revisions, please reply to this message or contact the journal office at plosone@plos.org. When you're ready to submit your revision, log on to https://www.editorialmanager.com/pone/ and select the 'Submissions Needing Revision' folder to locate your manuscript file.

- A rebuttal letter that responds to each point raised by the academic editor and reviewer(s). You should upload this letter as a separate file labeled 'Response to Reviewers'.
- A marked-up copy of your manuscript that highlights changes made to the original version. You should upload this as a separate file labeled 'Revised Manuscript with Track Changes'.
- An unmarked version of your revised paper without tracked changes. You should upload this as a separate file labeled 'Manuscript'.

If you would like to make changes to your financial disclosure, please include your updated statement in your cover letter. Guidelines for resubmitting your figure files are available below the reviewer comments at the end of this letter.

If applicable, we recommend that you deposit your laboratory protocols in protocols.io to enhance the reproducibility of your results. Protocols.io assigns your protocol its own identifier (DOI) so that it can be cited independently in the future. For instructions see: https://journals.plos.org/plosone/s/submission-guidelines#loc-laboratory-protocols. Additionally, PLOS ONE offers an option for publishing peer-reviewed Lab Protocol articles, which describe protocols hosted on protocols.io. Read more information on sharing protocols at https://plos.org/protocols?utm_medium=editorial-email&utm_source=authorletters&utm_campaign=protocols.

We look forward to receiving your revised manuscript.

Kind regards,

Dr Jason Morgan

Staff Editor

PLOS ONE

Journal Requirements:

When submitting your revision, we need you to address these additional requirements.

1. Please ensure that your manuscript meets PLOS ONE's style requirements, including those for file naming. The PLOS ONE style templates can be found at

https://journals.plos.org/plosone/s/file?id=wjVg/PLOSOne_formatting_sample_main_body.pdf and

https://journals.plos.org/plosone/s/file?id=ba62/PLOSOne_formatting_sample_title_authors_affiliations.pdf.

2. Please note that PLOS One has specific guidelines on code sharing for submissions in which author-generated code underpins the findings in the manuscript. In these cases, we expect all author-generated code to be made available without restrictions upon publication of the work. Please review our guidelines at https://journals.plos.org/plosone/s/materials-and-software-sharing#loc-sharing-code and ensure that your code is shared in a way that follows best practice and facilitates reproducibility and reuse.

3. In your Methods section, please include additional information about your dataset and ensure that you have included a statement specifying whether the collection and analysis method complied with the terms and conditions for the source of the data

4. Thank you for stating the following financial disclosure:

“This research was funded by Shaanxi Provincial Key Laboratory of Sport Management and Cognitive Decision Neuroscience (Higher Education Institutions). -From Jianlan Ding

This research was funded by Xi'an Social Sciences Planning Fund Projects, grant number 25TY85. -From Xueli Li”

Please state what role the funders took in the study.  If the funders had no role, please state: "The funders had no role in study design, data collection and analysis, decision to publish, or preparation of the manuscript."

If this statement is not correct you must amend it as needed.

Please include this amended Role of Funder statement in your cover letter; we will change the online submission form on your behalf.

5. Please ensure that you include a title page within your main document. You should list all authors and all affiliations as per our author instructions and clearly indicate the corresponding author.

6. If the reviewer comments include a recommendation to cite specific previously published works, please review and evaluate these publications to determine whether they are relevant and should be cited. There is no requirement to cite these works unless the editor has indicated otherwise.

[Note: HTML markup is below. Please do not edit.]

Reviewers' comments:

Reviewer's Responses to Questions

**Comments to the Author**

1. Is the manuscript technically sound, and do the data support the conclusions?

Reviewer #1: Yes

**********

2. Has the statistical analysis been performed appropriately and rigorously?

Reviewer #1: Yes

**********

3. Have the authors made all data underlying the findings in their manuscript fully available?

The PLOS Data policy

Reviewer #1: No

**********

4. Is the manuscript presented in an intelligible fashion and written in standard English?

Reviewer #1: Yes

**********

Reviewer #1: This manuscript presents an innovative effort to measure destination image dissonance through various media sources using Compositional Data Analysis (CoDA). The topics raised are relevant and methodologically promising, but some aspects need to be substantially revised to meet the standards of scientific publications. Conceptually, this research requires the formulation of clearer research questions and stronger justifications regarding the use of CoDA in capturing mismatches in perceptions between media. The relationship between compositional metrics and the cognitive–affective–spatial dimensions of destination imagery needs to be explained more explicitly. The literature review is informative but still descriptive and needs to be synergized with the latest studies (2020–2024) related to cross-media analysis and digital image formation. In terms of methodology, although the mathematical explanation of the CoDA has been correct, data validation and yield robustness tests have not been presented. The author needs to add information regarding the reliability of keyword extraction, handling of zero values, and statistical significance tests to strengthen the results claim. The results and discussion sections are still descriptive; interpretation needs to be linked to the existing Tourist Destination Image theory. The language and structure of the writing are quite good, but they need professional editing to be more consistent. In addition, the availability of data needs to be clarified through open repositories to support research replication.

**********

what does this mean?). If published, this will include your full peer review and any attached files.

If you choose “no”, your identity will remain anonymous but your review may still be made public.

**Do you want your identity to be public for this peer review?**  For information about this choice, including consent withdrawal, please see our Privacy Policy

Reviewer #1: No

**********

[NOTE: If reviewer comments were submitted as an attachment file, they will be attached to this email and accessible via the submission site. Please log into your account, locate the manuscript record, and check for the action link "View Attachments". If this link does not appear, there are no attachment files.]

While revising your submission, please upload your figure files to the Preflight Analysis and Conversion Engine (PACE) digital diagnostic tool, https://pacev2.apexcovantage.com/. PACE helps ensure that figures meet PLOS requirements. To use PACE, you must first register as a user. Registration is free. Then, login and navigate to the UPLOAD tab, where you will find detailed instructions on how to use the tool. If you encounter any issues or have any questions when using PACE, please email PLOS at figures@plos.org. Please note that Supporting Information files do not need this step.

Attachment Submitted filename: Review 1.pdf

# Author comment: Author response to Decision Letter 1

**DOI:** 10.1371/journal.pone.0360383.r002

17 Nov 2025

We sincerely appreciate you taking the precious time out of your busy schedule to conduct an extremely meticulous review of our manuscript and provide highly professional and insightful comments and suggestions. Your review is not only rigorous, thorough and pinpointed—accurately identifying the shortcomings and areas for improvement in our research—but also offers us a wealth of inspiring ideas with your profound professional expertise, illuminating a clear direction for the refinement and optimization of the entire study.

Upon receiving your review comments, our team promptly held in-depth discussions on each comment one by one, carefully deliberating over every suggestion and fully integrating them into the manuscript revision process. From deepening and improving the theoretical framework, supplementing and strengthening methodological validation, to enriching and optimizing visual presentations, and further enhancing the tight connection between research results and theoretical logic, every revision embodies your professional insights. It is precisely these highly targeted and valuable suggestions that have helped us effectively address the weaknesses in our research, significantly improving the academic rigor, logical clarity and practical guidance of the entire study, while also deepening our understanding of the research topic.

We would like to extend our most sincere gratitude to you once again! Thank you for your generous sharing of professional feedback and selfless contribution of academic wisdom—every effort you have made provides crucial support for enhancing the quality of this manuscript. We sincerely hope that the revised manuscript meets the publication requirements and earnestly request your continued valuable guidance and corrections.

Attachment Submitted filename: Point-by-Point Response to Reviewer Comments.docx

# Peer review: Decision Letter 1

**DOI:** 10.1371/journal.pone.0360383.r003

**Authors:** Sulemana Bankuoru Egala

22 Dec 2025

Dear Dr. Li,<o:p></o:p>

Thank you for submitting your manuscript to PLOS ONE. After careful consideration, we feel that a significant revision has been done on the manuscript. However, there are some few outstanding issues to address to fully meet PLOS ONE’s publication criteria as it currently stands. Therefore, we invite you to submit a revised version of the manuscript that addresses the few points raised during the review process particularly by reviewer 2.<o:p></o:p>

Please submit your revised manuscript by  Feb 05 2026 11:59PM. If you will need more time than this to complete your revisions, please reply to this message or contact the journal office at plosone@plos.org. When you're ready to submit your revision, log on to https://www.editorialmanager.com/pone/ and select the 'Submissions Needing Revision' folder to locate your manuscript file.<o:p></o:p>

Please include the following items when submitting your revised manuscript:<o:p></o:p>

- A rebuttal letter that responds to each point raised by the academic editor and reviewer(s). You should upload this letter as a separate file labeled 'Response to Reviewers'.<o:p></o:p>

- A marked-up copy of your manuscript that highlights changes made to the original version. You should upload this as a separate file labeled 'Revised Manuscript with Track Changes'.<o:p></o:p>

- An unmarked version of your revised paper without tracked changes. You should upload this as a separate file labeled 'Manuscript'.<o:p></o:p>

If you would like to make changes to your financial disclosure, please include your updated statement in your cover letter. Guidelines for resubmitting your figure files are available below the reviewer comments at the end of this letter.<o:p></o:p>

If applicable, we recommend that you deposit your laboratory protocols in protocols.io to enhance the reproducibility of your results. Protocols.io assigns your protocol its own identifier (DOI) so that it can be cited independently in the future. For instructions see: https://journals.plos.org/plosone/s/submission-guidelines#loc-laboratory-protocols. Additionally, PLOS ONE offers an option for publishing peer-reviewed Lab Protocol articles, which describe protocols hosted on protocols.io. Read more information on sharing protocols at https://plos.org/protocols?utm_medium=editorial-email&utm_source=authorletters&utm_campaign=protocols.<o:p></o:p>

Thank you for chosing PlosOne and we look forward to receiving your revised manuscript soon.<o:p></o:p>

Kind regards,

Sulemana Bankuoru Egala

Academic Editor

PLOS One

Journal Requirements:

1. If the reviewer comments include a recommendation to cite specific previously published works, please review and evaluate these publications to determine whether they are relevant and should be cited. There is no requirement to cite these works unless the editor has indicated otherwise.

2. Please review your reference list to ensure that it is complete and correct. If you have cited papers that have been retracted, please include the rationale for doing so in the manuscript text, or remove these references and replace them with relevant current references. Any changes to the reference list should be mentioned in the rebuttal letter that accompanies your revised manuscript. If you need to cite a retracted article, indicate the article’s retracted status in the References list and also include a citation and full reference for the retraction notice.

[Note: HTML markup is below. Please do not edit.]

Reviewers' comments:

Reviewer's Responses to Questions

**Comments to the Author**

Reviewer #1: All comments have been addressed

Reviewer #2: All comments have been addressed

**********

2. Is the manuscript technically sound, and do the data support the conclusions?

Reviewer #1: Yes

Reviewer #2: Partly

**********

3. Has the statistical analysis been performed appropriately and rigorously?

Reviewer #1: Yes

Reviewer #2: N/A

**********

4. Have the authors made all data underlying the findings in their manuscript fully available?

The PLOS Data policy

Reviewer #1: Yes

Reviewer #2: No

**********

5. Is the manuscript presented in an intelligible fashion and written in standard English?

Reviewer #1: Yes

Reviewer #2: No

**********

Reviewer #1: Based on the revised and reviewed draft of the article, no significant errors or deficiencies were found.

Reviewer #2: Dear author, thank you for the revisions made. I have a few more considerations about this revised manuscript.

A) Introduction - Although the gap was improved, it is not theoretical sound in terms of justification.

In some parts of your text, you have too many long sentences. For example, the paragraph about the aim.

Consider making the sentences shorter and more direct.

B) Literature review - Just call it “Tourism destination image”, since some core studies were neglected.

Despite some improvements, this section new improvements, in terms of destination image (DI) formation, and DI implications.

C) I miss a specific discussion section

D) Conclusion - as is, it reads more as a discussion not a real conclusion. Revise this section.

**********

what does this mean?). If published, this will include your full peer review and any attached files.

If you choose “no”, your identity will remain anonymous but your review may still be made public.

**Do you want your identity to be public for this peer review?**  For information about this choice, including consent withdrawal, please see our Privacy Policy

Reviewer #1: No

Reviewer #2: No

**********

[NOTE: If reviewer comments were submitted as an attachment file, they will be attached to this email and accessible via the submission site. Please log into your account, locate the manuscript record, and check for the action link "View Attachments". If this link does not appear, there are no attachment files.]

To ensure your figures meet our technical requirements, please review our figure guidelines: https://journals.plos.org/plosone/s/figures

You may also use PLOS’s free figure tool, NAAS, to help you prepare publication quality figures: https://journals.plos.org/plosone/s/figures#loc-tools-for-figure-preparation.

NAAS will assess whether your figures meet our technical requirements by comparing each figure against our figure specifications.

# Author comment: Author response to Decision Letter 2

**DOI:** 10.1371/journal.pone.0360383.r004

2 Jan 2026

Dear Reviewer,

We sincerely appreciate your thorough and insightful review of our manuscript. Your constructive comments have been invaluable in helping us to significantly strengthen the theoretical rigor, clarity, and overall structure of the paper. We have carefully considered and addressed each of your points. Our point-by-point responses and the corresponding revisions are detailed below.

Comment A) Introduction - Although the gap was improved, it is not theoretical sound in terms of justification. In some parts of your text, you have too many long sentences. For example, the paragraph about the aim. Consider making the sentences shorter and more direct.

Response:We are very grateful for this critical observation. In response, we have substantially revised the Introduction section to enhance its theoretical foundation and improve readability.

1.Strengthening Theoretical Justification: To provide a more solid theoretical grounding, we have now explicitly cited key literature that underpins the cognitive-affective model (Baloglu & McCleary, 1999), justifies the inclusion of the spatial dimension (Lynch, 1960), and supports the use of Compositional Data Analysis (Aitchison, 1986; Coenders & Ferrer-Rosell, 2020). This strengthens the rationale for our tripartite “cognitive-spatial-affective” framework and methodological choice.

2.Simplifying Sentence Structure: As suggested, we have broken down lengthy and complex sentences into shorter, more direct statements. This is particularly evident in the paragraph stating the research aims, which has been rewritten into a concise introductory sentence followed by a clearly itemized list of objectives (see the second-to-last paragraph of the revised Introduction). This revision enhances clarity and eases comprehension.

Comment B) Literature review - Just call it “Tourism destination image”, since some core studies were neglected. Despite some improvements, this section needs improvements, in terms of destination image (DI) formation, and DI implications.

Response: Thank you for this suggestion, which prompted us to refine the focus of our literature review.

Regarding the title, we have restructured the section to better align with the core theme. We believe this provides a more precise roadmap for our specific study design than a generic title. However, we are happy to adopt your suggested title if you deem it more appropriate upon review of our revisions.

Concerning the content, we understand your call for a broader discussion of DI formation and implications. Our literature review is intentionally structured to build a direct foundation for our study's core focus: the measurement of differences across sources and dimensions, rather than a comprehensive review of all TDI facets.

Comment C) I miss a specific discussion section.

Response: We completely agree with this essential structural suggestion. As recommended, we have added a dedicated “Discussion” section as the new Chapter 5 (subsequent chapter numbers have been updated accordingly). In this section, we:

Interpret our key findings (e.g., the “institutional narrative alliance” vs. “tourist experiential narrative”) by engaging with relevant theories from tourism geography and media studies.

Explain how the COVID-19 pandemic acted as a “catalyst” amplifying existing dissonances, discussing underlying mechanisms like institutional path dependence.

Reflect on the methodological contributions and limitations of applying the CoDA framework to tourism image analysis.

Elucidate the broader theoretical and practical implications of our results.

Comment D) Conclusion - as is, it reads more as a discussion not a real conclusion. Revise this section.

Response: Thank you for this correction. We have thoroughly revised the Conclusion section (now Section 6.1) to make it more concise and summative. We have removed explanatory and discursive content, which has been moved to the new Discussion chapter. The conclusion now concisely restates the three core findings of the study in direct, declarative statements, providing a clear answer to the original research questions. It ends with a brief sentence highlighting the main takeaway for destination image management, ensuring the section serves as a true summary of the paper’s outcomes.

We believe these revisions have comprehensively addressed your valuable comments and greatly improved the manuscript. Thank you again for your time and guidance. Wishing you and your family a happy New Year!

Sincerely,

The Authors

Attachment Submitted filename: Response to Reviewers.docx

# Peer review: Decision Letter 2

**DOI:** 10.1371/journal.pone.0360383.r005

**Authors:** Sulemana Bankuoru Egala

18 Jan 2026

Dear Dr. Li,

Thank you for submitting your manuscript to PLOS ONE. After careful consideration, we feel that it has merit but does not fully meet PLOS ONE’s publication criteria as it currently stands. Hence, we have reacehed a minor revision decision to enable you thoroughly revised the manuscript. Therefore, we invite you to submit a revised version of the manuscript that addresses the points raised during the review process. Particularly, reviewer 1 raised some minor but critical comments that needs to be addresed to improved on the quality of the manuscript.

Please submit your revised manuscript by Mar 04 2026 11:59PM. If you will need more time than this to complete your revisions, please reply to this message or contact the journal office at plosone@plos.org. When you're ready to submit your revision, log on to https://www.editorialmanager.com/pone/ and select the 'Submissions Needing Revision' folder to locate your manuscript file.

- A letter that responds to each point raised by the academic editor and reviewer(s). You should upload this letter as a separate file labeled 'Response to Reviewers'.
- A marked-up copy of your manuscript that highlights changes made to the original version. You should upload this as a separate file labeled 'Revised Manuscript with Track Changes'.
- An unmarked version of your revised paper without tracked changes. You should upload this as a separate file labeled 'Manuscript'.

If applicable, we recommend that you deposit your laboratory protocols in protocols.io to enhance the reproducibility of your results. Protocols.io assigns your protocol its own identifier (DOI) so that it can be cited independently in the future. For instructions see: https://journals.plos.org/plosone/s/submission-guidelines#loc-laboratory-protocols. Additionally, PLOS ONE offers an option for publishing peer-reviewed Lab Protocol articles, which describe protocols hosted on protocols.io. Read more information on sharing protocols at https://plos.org/protocols?utm_medium=editorial-email&utm_source=authorletters&utm_campaign=protocols.

We look forward to receiving your revised manuscript.

Kind regards,

Sulemana Bankuoru Egala

Academic Editor

PLOS One

Journal Requirements:

1. If the reviewer comments include a recommendation to cite specific previously published works, please review and evaluate these publications to determine whether they are relevant and should be cited. There is no requirement to cite these works unless the editor has indicated otherwise.

2. Please review your reference list to ensure that it is complete and correct. If you have cited papers that have been retracted, please include the rationale for doing so in the manuscript text, or remove these references and replace them with relevant current references. Any changes to the reference list should be mentioned in the rebuttal letter that accompanies your revised manuscript. If you need to cite a retracted article, indicate the article’s retracted status in the References list and also include a citation and full reference for the retraction notice.

[Note: HTML markup is below. Please do not edit.]

Reviewers' comments:

Reviewer's Responses to Questions

**Comments to the Author**

Reviewer #1: All comments have been addressed

Reviewer #2: All comments have been addressed

**********

2. Is the manuscript technically sound, and do the data support the conclusions?

Reviewer #1: Yes

Reviewer #2: Yes

**********

3. Has the statistical analysis been performed appropriately and rigorously?

Reviewer #1: Yes

Reviewer #2: Yes

**********

4. Have the authors made all data underlying the findings in their manuscript fully available?

The PLOS Data policy

Reviewer #1: No

Reviewer #2: No

**********

5. Is the manuscript presented in an intelligible fashion and written in standard English?

Reviewer #1: Yes

Reviewer #2: Yes

**********

Reviewer #1: Judgment for point 4:

No, the authors have not yet made all data underlying the findings fully available in a manner compliant with PLOS ONE policy.

Recommended action (Minor Revision – Mandatory Compliance Fix):

a. Deposit processed datasets and code in a public repository.

b. Revise the Data Availability Statement to include a working URL/DOI.

c. Clearly distinguish between restricted raw text data and fully shareable analytical outputs.

Reviewer #2: I believe improvements made to the manuscritp have strengthen it. My last comment is:

Avoid adding numbered bullets to your discussion and conclusion.

**********

what does this mean?). If published, this will include your full peer review and any attached files.

If you choose “no”, your identity will remain anonymous but your review may still be made public.

**Do you want your identity to be public for this peer review?**  For information about this choice, including consent withdrawal, please see our Privacy Policy

Reviewer #1: No

Reviewer #2: No

**********

[NOTE: If reviewer comments were submitted as an attachment file, they will be attached to this email and accessible via the submission site. Please log into your account, locate the manuscript record, and check for the action link "View Attachments". If this link does not appear, there are no attachment files.]

To ensure your figures meet our technical requirements, please review our figure guidelines: https://journals.plos.org/plosone/s/figures

You may also use PLOS’s free figure tool, NAAS, to help you prepare publication quality figures: https://journals.plos.org/plosone/s/figures#loc-tools-for-figure-preparation.

NAAS will assess whether your figures meet our technical requirements by comparing each figure against our figure specifications.

# Author comment: Author response to Decision Letter 3

**DOI:** 10.1371/journal.pone.0360383.r006

27 Jan 2026

Dear Reviewer,

We sincerely appreciate the valuable comments and suggestions you have provided, which have greatly helped us improve the quality of our manuscript. We have carefully addressed all the questions raised by the reviewers, and the specific revisions are as follows:

Response to Reviewer 1

We are very grateful for your comments on the data availability of this manuscript. We fully agree with your suggestions and have made all the necessary revisions as required to comply with the data policies of PLOS ONE. A detailed response to your specific suggestions is provided below:

Response to Point 4 (Data Availability)

We have uploaded all processed datasets and the corresponding analysis codes to a publicly accessible repository (GitHub, URL: https://github.com/x9w259pp86-stack/datesets-for-Plos-one.git ). All data and codes are complete and available for free access, download and reuse by other researchers, ensuring the reproducibility of the study results.

Response to Reviewer 2

We highly appreciate your recognition of the improvements made to this manuscript as well as your valuable final comments. We have carefully noted your suggestion to avoid using numbered bullet points in the Discussion and Conclusion sections and have made the corresponding revisions accordingly.

Specifically, we have removed all numbered bullet points from the "Discussion" and "Conclusion" sections of the manuscript and reorganized the content of these two sections into coherent paragraph-style descriptions.

In summary, we have carefully addressed all the comments raised by the two reviewers. All revisions are based on the original research content and academic norms, aiming to further enhance the rigor and completeness of the manuscript. We sincerely hope that the revised manuscript meets the publication requirements of PLOS ONE. We would like to express our sincere gratitude again for your careful review and valuable suggestions.

Attachment Submitted filename: Response_to_Reviewers_auresp_3.docx

# Peer review: Decision Letter 3

**DOI:** 10.1371/journal.pone.0360383.r007

**Authors:** Sulemana Bankuoru Egala

22 Apr 2026

Dear Dr.  Li,

Please submit your revised manuscript by Jun 06 2026 11:59PM. If you will need more time than this to complete your revisions, please reply to this message or contact the journal office at plosone@plos.org. When you're ready to submit your revision, log on to https://www.editorialmanager.com/pone/ and select the 'Submissions Needing Revision' folder to locate your manuscript file.

Please include the following items when submitting your revised manuscript:

- A letter that responds to each point raised by the academic editor and reviewer(s). You should upload this letter as a separate file labeled 'Response to Reviewers'.
- A marked-up copy of your manuscript that highlights changes made to the original version. You should upload this as a separate file labeled 'Revised Manuscript with Track Changes'.
- An unmarked version of your revised paper without tracked changes. You should upload this as a separate file labeled 'Manuscript'.

If you would like to make changes to your financial disclosure, please include your updated statement in your cover letter. Guidelines for resubmitting your figure files are available below the reviewer comments at the end of this letter.

If applicable, we recommend that you deposit your laboratory protocols in protocols.io to enhance the reproducibility of your results. Protocols.io assigns your protocol its own identifier (DOI) so that it can be cited independently in the future. For instructions see: https://journals.plos.org/plosone/s/submission-guidelines#loc-laboratory-protocols. Additionally, PLOS ONE offers an option for publishing peer-reviewed Lab Protocol articles, which describe protocols hosted on protocols.io. Read more information on sharing protocols at https://plos.org/protocols?utm_medium=editorial-email&utm_source=authorletters&utm_campaign=protocols.

As the corresponding author, your ORCID iD is verified in the submission system and will appear in the published article. PLOS supports the use of ORCID, and we encourage all coauthors to register for an ORCID iD and use it as well. Please encourage your coauthors to verify their ORCID iD within the submission system before final acceptance, as unverified ORCID iDs will not appear in the published article. *Only*  the individual author can complete the verification step; PLOS staff *cannot*  verify ORCID iDs on behalf of authors.

We look forward to receiving your revised manuscript.

Kind regards,

Sulemana Bankuoru Egala

Academic Editor

PLOS One

Journal Requirements:

1. If the reviewer comments include a recommendation to cite specific previously published works, please review and evaluate these publications to determine whether they are relevant and should be cited. There is no requirement to cite these works unless the editor has indicated otherwise.

2. Please review your reference list to ensure that it is complete and correct. If you have cited papers that have been retracted, please include the rationale for doing so in the manuscript text, or remove these references and replace them with relevant current references. Any changes to the reference list should be mentioned in the rebuttal letter that accompanies your revised manuscript. If you need to cite a retracted article, indicate the article’s retracted status in the References list and also include a citation and full reference for the retraction notice.

Additional Editor Comments:

While they commended the paper for its novelty, I recommend minor revisions. Aspects including aligning the data-availability statement with the GitHub repository link; clarifying the keyword selection and core components for the Dirichlet regression; and correcting several technical inconsistencies (e.g., terminology and labeling errors) as well as an incomplete sentence in the limitations section.

Once these revisions are made comprehensively, the paper will go throuhg another round of revision for verification and quality checks to ensure all the issues are addressed.

[Note: HTML markup is below. Please do not edit.]

Reviewer's Responses to Questions

**Comments to the Author**

Reviewer #1: All comments have been addressed

Reviewer #2: All comments have been addressed

Reviewer #3: All comments have been addressed

**********

2. Is the manuscript technically sound, and do the data support the conclusions?

Reviewer #1: Yes

Reviewer #2: Yes

Reviewer #3: Yes

**********

3. Has the statistical analysis been performed appropriately and rigorously?

Reviewer #1: Yes

Reviewer #2: N/A

Reviewer #3: Yes

**********

4. Have the authors made all data underlying the findings in their manuscript fully available?

The PLOS Data policy

Reviewer #1: No

Reviewer #2: Yes

Reviewer #3: No

**********

5. Is the manuscript presented in an intelligible fashion and written in standard English?

Reviewer #1: Yes

Reviewer #2: Yes

Reviewer #3: Yes

**********

Reviewer #1: The authors have not fully complied with the journal’s data availability requirements. Although they state that the data are available, no functional repository link, DOI, or accession number is provided. The indication that data may be shared upon request or embedded within the manuscript is insufficient and does not meet PLOS ONE’s policy on open data. The manuscript requires revision to ensure that all underlying data are deposited in a publicly accessible repository prior to publication.

Reviewer #2: (No Response)

Reviewer #3: The manuscript addresses an interesting and timely topic and makes a valuable contribution by integrating cognitive, spatial, and affective dimensions of destination image and applying compositional data analysis to compare PGC, TGC, and OGC over time. The longitudinal design and the focus on crisis-related shifts add practical relevance. Overall, the paper is promising and close to publishable, but I recommend minor revision before acceptance. First, the manuscript should resolve the inconsistency in the data-availability information: the response letter states that all processed datasets and code have been uploaded to a public GitHub repository, yet the data-availability statement still says the authors are unsure how to create a URL and are willing to share data by another method. These statements should be fully aligned and the repository link should be clearly presented in the manuscript.

Second, the methods section would benefit from a clearer explanation of how the 50 keywords were finalized from the top-100 lists and how the “core components” used in the Dirichlet regression were selected, since the current description is still somewhat concise relative to the importance of these analytic decisions.

Third, the manuscript needs careful technical editing: there is terminology inconsistency in the Discussion where PGC is defined as “Professional Generated Content” rather than “Press Generated Content,” Table 3 under the affective-image section is mislabeled as “Spatial component analysis,” and the limitations section begins with an incomplete sentence (“This may have resulted…”).

**********

what does this mean?). If published, this will include your full peer review and any attached files.

If you choose “no”, your identity will remain anonymous but your review may still be made public.

**Do you want your identity to be public for this peer review?**  For information about this choice, including consent withdrawal, please see our Privacy Policy

Reviewer #1: No

Reviewer #2: No

Reviewer #3: **Yes:**  Tingting Zhang

**********

[NOTE: If reviewer comments were submitted as an attachment file, they will be attached to this email and accessible via the submission site. Please log into your account, locate the manuscript record, and check for the action link "View Attachments". If this link does not appear, there are no attachment files.]

To ensure your figures meet our technical requirements, please review our figure guidelines: https://journals.plos.org/plosone/s/figures

You may also use PLOS’s free figure tool, NAAS, to help you prepare publication quality figures: https://journals.plos.org/plosone/s/figures#loc-tools-for-figure-preparation.

NAAS will assess whether your figures meet our technical requirements by comparing each figure against our figure specifications.

# Author comment: Author response to Decision Letter 4

**DOI:** 10.1371/journal.pone.0360383.r008

12 May 2026

Dear Reviewer,

We sincerely appreciate your thorough and insightful review of our manuscript. Your constructive comments have been invaluable in helping us to significantly strengthen the theoretical rigor, clarity, and overall structure of the paper. We have carefully considered and addressed each of your points. Our point-by-point responses and the corresponding revisions are detailed below.

Comment 1) First, the manuscript should resolve the inconsistency in the data-availability information: the response letter states that all processed datasets and code have been uploaded to a public GitHub repository, yet the data-availability statement still says the authors are unsure how to create a URL and are willing to share data by another method. These statements should be fully aligned and the repository link should be clearly presented in the manuscript.

Response: We sincerely appreciate this careful and important comment. We have completely unified and standardized all statements related to data availability in both the manuscript and the response letter to ensure full consistency. All processed datasets and the corresponding analysis codes in this study have been uploaded to a publicly accessible GitHub repository, and the URL has been clearly labeled in the “Data Availability” section of the manuscript.

The public repository is available at:

https://github.com/x9w259pp86-stack/datesets-for-Plos-one.git

All data and codes are complete, freely accessible, downloadable, and reusable by other researchers, which fully ensures the reproducibility of the study results.

Comment 2) Second, the methods section would benefit from a clearer explanation of how the 50 keywords were finalized from the top-100 lists and how the “core components” used in the Dirichlet regression were selected, since the current description is still somewhat concise relative to the importance of these analytic decisions.

Response:

Thank you for this constructive and professional suggestion. We have substantially supplemented and refined the Method section with detailed, rigorous, and replicable descriptions for both key analytical procedures:

1.We added a complete and transparent procedure for selecting the 50 core keywords from the top‑100 high-frequency word lists of PGC, TGC, and OGC, including the processes of extraction, merging, deduplication, cumulative frequency ranking, and final selection.

2.We added a comprehensive and logical explanation of the variable selection logic for Dirichlet regression, including two rigorous selection criteria (high contribution to Aitchison distance and theoretical importance in the three-dimensional destination image framework) and the selection of reference variables for log-ratio transformation.

The revised content ensures methodological transparency and matches the academic importance of these analytical decisions.

Comment 3) Third, the manuscript needs careful technical editing: there is terminology inconsistency in the Discussion where PGC is defined as “Professional Generated Content” rather than “Press Generated Content,” Table 3 under the affective-image section is mislabeled as “Spatial component analysis,” and the limitations section begins with an incomplete sentence (“This may have resulted…”).

Response: We highly appreciate your meticulous review. We have comprehensively checked and revised all technical inconsistencies, labeling errors, and incomplete sentences throughout the manuscript:

1.Terminology consistency: We have unified the full name of PGC as Press Generated Content.

2.Table label correction: We have corrected the mislabeled title of Table 3 from “Spatial Component Analysis” to Affective Component Analysis.

3.Incomplete sentence revision: We have revised the incomplete and ungrammatical opening sentence in the “Limitations” section (originally “This may have resulted…”) to ensure grammatical completeness, fluency, and academic accuracy.

All revisions have been double‑checked to ensure clarity, standardization, and consistency.

We believe these revisions have comprehensively addressed your valuable comments and greatly improved the manuscript. Thank you again for your time and guidance.

Sincerely,

The Authors

Attachment Submitted filename: Response_to_Reviewers_auresp_4.docx

# Peer review: Decision Letter 4

**DOI:** 10.1371/journal.pone.0360383.r009

**Authors:** Sulemana Bankuoru Egala

3 Jun 2026

Dear Dr. Li,

Specifically, reviewer 4 identifies two fatal flaws. 1) The analysis cannot distinguish time trends from COVID impacts because only two time periods are used (requiring yearly or post-COVID data), and the claimed GitLab dataset is incomplete, lacking word labelling, raw frequencies, and necessary metadata to verify any results. 2) Serious issues include faulty Dirichlet regression (ignoring conditional independence with only two data points), incomplete formulas, unsupported claims, and confusion between frequencies and relative frequencies.

Please submit your revised manuscript by Jul 18 2026 11:59PM. If you will need more time than this to complete your revisions, please reply to this message or contact the journal office at plosone@plos.org. When you're ready to submit your revision, log on to https://www.editorialmanager.com/pone/ and select the 'Submissions Needing Revision' folder to locate your manuscript file.

- A letter that responds to each point raised by the academic editor and reviewer(s). You should upload this letter as a separate file labeled 'Response to Reviewers'.
- A marked-up copy of your manuscript that highlights changes made to the original version. You should upload this as a separate file labeled 'Revised Manuscript with Track Changes'.
- An unmarked version of your revised paper without tracked changes. You should upload this as a separate file labeled 'Manuscript'.

If you would like to make changes to your financial disclosure, please include your updated statement in your cover letter. Guidelines for resubmitting your figure files are available below the reviewer comments at the end of this letter.

If applicable, we recommend that you deposit your laboratory protocols in protocols.io to enhance the reproducibility of your results. Protocols.io assigns your protocol its own identifier (DOI) so that it can be cited independently in the future. For instructions see: https://journals.plos.org/plosone/s/submission-guidelines#loc-laboratory-protocols. Additionally, PLOS ONE offers an option for publishing peer-reviewed Lab Protocol articles, which describe protocols hosted on protocols.io. Read more information on sharing protocols at https://plos.org/protocols?utm_medium=editorial-email&utm_source=authorletters&utm_campaign=protocols.

As the corresponding author, your ORCID iD is verified in the submission system and will appear in the published article. PLOS supports the use of ORCID, and we encourage all coauthors to register for an ORCID iD and use it as well. Please encourage your coauthors to verify their ORCID iD within the submission system before final acceptance, as unverified ORCID iDs will not appear in the published article. *Only*  the individual author can complete the verification step; PLOS staff *cannot*  verify ORCID iDs on behalf of authors.

We look forward to receiving your revised manuscript.

Kind regards,

Sulemana Bankuoru Egala (PhD)

Academic Editor

PLOS One

Journal Requirements:

If the reviewer comments include a recommendation to cite specific previously published works, please review and evaluate these publications to determine whether they are relevant and should be cited. There is no requirement to cite these works unless the editor has indicated otherwise.

Please review your reference list to ensure that it is complete and correct. If you have cited papers that have been retracted, please include the rationale for doing so in the manuscript text, or remove these references and replace them with relevant current references. Any changes to the reference list should be mentioned in the rebuttal letter that accompanies your revised manuscript. If you need to cite a retracted article, indicate the article’s retracted status in the References list and also include a citation and full reference for the retraction notice.

[Note: HTML markup is below. Please do not edit.]

Reviewers' comments:

Reviewer's Responses to Questions

**Comments to the Author**

Reviewer #1: All comments have been addressed

Reviewer #3: All comments have been addressed

Reviewer #4: (No Response)

**********

2. Is the manuscript technically sound, and do the data support the conclusions?

Reviewer #1: Yes

Reviewer #3: Yes

Reviewer #4: Partly

**********

3. Has the statistical analysis been performed appropriately and rigorously?

Reviewer #1: Yes

Reviewer #3: Yes

Reviewer #4: No

**********

4. Have the authors made all data underlying the findings in their manuscript fully available?

The PLOS Data policy

Reviewer #1: Yes

Reviewer #3: Yes

Reviewer #4: No

**********

5. Is the manuscript presented in an intelligible fashion and written in standard English?

Reviewer #1: Yes

Reviewer #3: Yes

Reviewer #4: Yes

**********

Reviewer #1: The author has attempted to improve all input and suggestions from reviewers regarding shortcomings in the previous draft of the article.

Reviewer #3: Thank you for your careful and constructive revisions. I appreciate the authors’ efforts to address the comments raised in the previous round of review. The revised manuscript is substantially improved in methodological transparency, data availability, and technical presentation. Congratulations!

Reviewer #4: Please see the attached file, which explains my reasons for requiring revisions before publication.

**********

what does this mean?). If published, this will include your full peer review and any attached files.

If you choose “no”, your identity will remain anonymous but your review may still be made public.

**Do you want your identity to be public for this peer review?**  For information about this choice, including consent withdrawal, please see our Privacy Policy

Reviewer #1: No

Reviewer #3: **Yes:**  Tingting Zhang

Reviewer #4: **Yes:**  John Bacon-Shone

**********

[NOTE: If reviewer comments were submitted as an attachment file, they will be attached to this email and accessible via the submission site. Please log into your account, locate the manuscript record, and check for the action link "View Attachments". If this link does not appear, there are no attachment files.]

To ensure your figures meet our technical requirements, please review our figure guidelines: https://journals.plos.org/plosone/s/figures

You may also use PLOS’s free figure tool, NAAS, to help you prepare publication quality figures: https://journals.plos.org/plosone/s/figures#loc-tools-for-figure-preparation.

NAAS will assess whether your figures meet our technical requirements by comparing each figure against our figure specifications.

Attachment Submitted filename: Review of Measuring Destination Image Dissonance.pdf

# Author comment: Author response to Decision Letter 5

**DOI:** 10.1371/journal.pone.0360383.r010

16 Jul 2026

Point-by-Point Response to Reviewer Comments

Dear Reviewer Bacon-Shone,,

We sincerely appreciate your rigorous, thorough and constructive remarks spanning empirical design, data transparency, statistical methodology, variable definition and result interpretation. Every issue you raised has been fully resolved via targeted manuscript revisions, supplementary open dataset uploads and expanded methodological elaborations. We provide a detailed point-by-point response to each of your comments as follows.

Comment 1 & Comment 8

Reviewer’s Comment

The abstract refers to a time lag, but the analysis fails to include the temporal dimension properly – it is impossible in this analysis to distinguish time trends from COVID impacts, making all the conclusions of COVID impact impossible to verify. This can be addressed by using yearly data, or at the very least dividing the pre-COVID period into multiple time periods. It would also greatly strengthen the analysis to add a post-COVID period, to see whether the disparities reduce.

Unsupported claims (see line 352) – as mentioned above, this analysis can NOT distinguish time trends from COVID impacts.

Response:

We fully agree with the reviewer that the two-stage comparative design (2013–2019 vs. 2020–2022) cannot statistically disentangle long-term secular trends from pandemic-specific shocks, meaning causal claims about COVID-19 effects are not empirically justified by our research setup. We have carried out four rounds of manuscript-wide revisions to resolve this issue:

1.All causal wording attributing image dissonance to the pandemic (e.g., “exacerbated”, “caused”) has been eliminated. All relevant statements are rewritten as neutral descriptive temporal comparisons, such as “the narrative divergence widened substantially in the 2020–2022 phase relative to the pre-pandemic period” and “structural misalignment appears more prominent during the pandemic-affected stage”. All findings are now presented as descriptive temporal contrasts without causal attribution.

2.The unsupported statement regarding a “3–5 year time lag” has been completely removed, as this numerical estimate cannot be derived from our dataset.

3.Supplementary methodological clarification is added at the end of Section 3.3 Compositional Data Analysis. We explicitly state that the two-period grouping only serves to describe stage-level structural discrepancies and cannot support formal significance testing or causal inference. All Aitchison distance values are interpreted merely as descriptive indicators of compositional dissimilarity.

4.A dedicated paragraph expanding on this temporal limitation is added to Section 6.4 Limitations, elaborating on how coarse temporal granularity restricts causal interpretation of cross-stage differences.

We acknowledge that annual subdivision or extended post-pandemic data would improve causal identification, yet such data expansion lies beyond the scope of the current study. We have listed annual and post-COVID longitudinal analysis as a clear direction for future research in the discussion section.

Comment 2

Reviewer’s Comment

The claim that the data is all available in gitlab is simply untrue. There is no labelling of the 50 words, in particular, as to which are cognitive, spatial and affective, let alone which link to the specific elements referenced in the analysis. Tourism, hotels, coastal food are referenced in the abstract without any connection to the data set provided! The dataset is already converted to relative frequencies and zeros removed, making it impossible to understand (for example), how the zero replacement affects the results. The dataset must include raw frequencies with full labelling provided.

Response

We apologize for the incomplete and disorganized dataset previously uploaded. We have fully resolved this transparency requirement by re-uploading complete supplementary datasets to the open repository:

https://github.com/x9w259pp86-stack/datesets-for-Plos-one/blob/main/datasets-updates.zip

The updated repository now contains the following complete materials to meet the reviewer’s requirements:

1.Raw absolute frequency matrices for all three media sources (PGC/TGC/OGC), retaining original zero-count entries without prior deletion or transformation;

2.A full classification label sheet for the 50 core keywords, clearly marking each term’s corresponding dimension (cognitive / spatial / affective), with direct cross-references to all thematic elements mentioned in the abstract and main text (tourism, hotels, coastal food, duty-free shops, Coconut Dream Corridor, etc.);

3.Intermediate datasets including unprocessed candidate keyword pools, pre-zero-imputation raw tables, and post-CLR transformation proportional data, allowing readers to trace the full impact of Bayesian zero replacement on final analytical outputs.

All data files are fully annotated with readme documents to ensure reproducibility of all keyword screening and CoDA procedures.

Comment 3

Reviewer’s Comment

Compositional data analysis (CoDA) (lines 130 onwards) is one way, not the only way to examine relative frequencies and correspondence analysis (CA) has a very specific advantage over CoDA when starting with frequency data in that it accounts for the count variability (i.e. for Poisson count data, variance is proportional to the mean) and avoids any need to make adjustments for zeros. This should be referenced as an alternative which avoids the problems of zeros and yet also provides a distance metric.

Response

We greatly appreciate this critical and constructive comment regarding alternative analytical approaches for keyword frequency data in tourism research. While we have adopted standard Bayesian multiplicative zero replacement to resolve zero-count obstacles within compositional data analysis (CoDA), we fully recognize that correspondence analysis (CA) is a widely used alternative suitable for raw absolute frequency matrices.

As the reviewer highlighted, CA exhibits an inherent strength for Poisson-distributed count data, as it requires no preliminary zero-imputation procedure to conduct subsequent analysis. Nevertheless, there exists an essential distinction in the core analytical focus between CA and CoDA that underpins our method selection. We have supplemented authoritative literature on correspondence analysis in the revised manuscript, systematically compared the strengths and limitations of the two techniques, and elaborated on why CoDA is more aligned with our research purpose:

This study centers on cross-media dissonance in the proportional composition of destination image elements, rather than disparities in absolute keyword occurrence counts. All media text data naturally follow the unit-sum closed constraint, where the relative weight of spatial, cognitive and affective keywords carries substantive theoretical meaning for measuring image imbalance. Centered log-ratio (CLR) transformation and the resulting Aitchison distance are dedicated compositional metrics designed to quantify divergence in proportional structures under closed-sum constraints. In contrast, CA mainly identifies differences in raw count distributions based on chi-square distance, which cannot independently extract structural deviations of proportional compositions. For this reason, CoDA is prioritized over CA in the present research, with Bayesian multiplicative zero replacement serving only as a standardized preprocessing step to eliminate computational barriers caused by missing word counts.

Comment 4

Reviewer’s Comment

The paper confuses frequencies and relative frequencies in several places (see line 125) – relative frequencies add to 1 and hence have the compositional constraint, but this is not true for frequencies.

Response

We sincerely appreciate the reviewer for identifying the confusion between absolute frequencies and proportional values throughout the manuscript. We fully acknowledge that unqualified use of the term “frequency” created ambiguous terminology and blurred the mathematical distinction between raw absolute counts and transformed relative proportions.

We have implemented targeted revisions across all relevant sections:

1.Section 2.3: Rewrote the problematic sentence that incorrectly treated raw keyword counts as compositional data. The revised text clarifies that only data converted into proportions follow the constant-sum constraint, whereas raw absolute frequencies do not carry this property.

2.Section 3.3: Added the modifier “absolute” to all unqualified “frequency” references, inserted a fixed total word volume constraint to correct the logical misunderstanding of how absolute counts interact with proportions, and explicitly stated that the constant-sum constraint exclusively applies to proportional data.

3.Unified analytical matrix subsection: Supplemented “absolute” to all cumulative and overall frequency descriptors to clarify that keyword screening was based on raw absolute counts rather than proportions.

4.Handling of Zero Values subsection: Revised “zero frequency values” to “zero absolute frequency” to specify that missing entries refer to raw count zeros, not proportional zeros.

5.Calculation of Aitchison Distance subsection: Added brief wording to anchor the constant-sum constraint to proportional data for consistent terminology.

6.Section 4. Results: Reframed vague references to “keyword differences” as “structural differences in keyword proportions” to make clear that all CoDA comparisons target proportional structures rather than raw absolute counts.

Throughout the revised manuscript, we strictly separate two core concepts: raw absolute frequency (the raw count of keyword occurrences) and proportion/relative frequency (standardized values summing to one with a constant-sum constraint). All statistical operations including zero replacement, centred log-ratio transformation, Aitchison distance calculation are clearly linked to proportional datasets, eliminating cross-concept confusion entirely.

Comment 5

Reviewer’s Comment

Data sources – there is no justification provided for the specific 3 sources chosen (e.g. what proportion of travellers use Ctrip?)

Response

We sincerely appreciate the reviewer’s constructive suggestion regarding the adequacy of data source justification. Corresponding revisions have been fully supplemented in Section 3.1, as detailed below:

1.At the opening paragraph of Section 3.1 Data Sources and Collection, we added theoretical rationale for selecting three complementary media categories (PGC, TGC, OGC). Combined analysis of official institutional narratives, authoritative media narratives and spontaneous tourist experiential narratives overcomes the one-sided defects of dual-source comparison widely adopted in existing tourism research, which clarifies why we adopt three distinct data sources.

2.In the subsection "Tourist-Perceived Image Data", we cite the official 2025 report released by QuestMobile with an accessible report link attached. These quantitative market share data directly address the reviewer’s question regarding traveller coverage on the Ctrip platform and fully demonstrate the representativeness of the Ctrip travelogue samples.

The above revisions systematically explain the rationality of the triple-source framework and provide empirical quantitative evidence for the representativeness of Ctrip TGC data, fully addressing all concerns raised in this comment.

Comment 6

Reviewer’s Comment

The paper makes the claim (line 283) that the criterion ensures the elements are the most prevalent, which is true, but fails to address whether 50 key words are sufficient, for example, what proportion of all key word references are included in the 50 key words selected?

Response

We appreciate the reviewer’s important reminder regarding the lack of quantitative evidence to verify the sufficiency of the 50 core keywords. Corresponding supplementary calculations and explanations have been added to the subsection Construction of a unified analytical matrix.

We supplemented the explicit coverage rate formula in the text:

Coverage rate = Cumulative absolute frequency of the top 50 screened core keywords ÷ Total cumulative absolute frequency of all unique candidate keywords merged from three media sources.

We calculated coverage rates separately for the two research periods:

�Pre-pandemic stage (2013–2019): 78.22%

�Pandemic impact and recovery stage (2020–2022): 79.23%

Following the word set selection criteria proposed by Liu et al. (2016), a cumulative word frequency coverage of 70% acts as the critical cutoff separating informative high- and medium-frequency terms from trivial long-tail low-frequency words. The two coverage rates calculated in this study both surpass the 70% threshold and fall within the optimal range of 70%–80%. This demonstrates that the 50 screened core keywords sufficiently capture the dominant semantic features of textual data on Sanya’s tourism and are appropriate for subsequent compositional data analysis.

Comment 7

Reviewer’s Comment

Formulas 2 and 3 are both incomplete – what is si and what are the summation limits? Formula 3 is clearly faulty.

Response

We sincerely appreciate the reviewer for identifying multiple flaws in the self-defined formulas for zero-value processing in the original manuscript, such as undefined variables, missing summation ranges, and logical errors arising from circular formula construction. Although these formulas were adapted from existing literature, we have double-checked them and fully acknowledge all the above mathematical defects.

During the actual data processing, we never utilised these flawed custom formulas. All preprocessing for zero values was implemented via the standard Bayesian-multiplicative zero replacement algorithm built into the R package zCompositions, which strictly follows the authoritative algorithm framework proposed by Martín‑Fernández et al. (2015). The full executable R code for zero replacement is attached in Appendix for reproducibility.

To eliminate misleading mathematical expressions and avoid confusion for readers, we have removed all formulas throughout the revised manuscript. This includes not only the problematic zero-replacement formulas but also the transformation and distance calculation formulas that were retained previously. Every mathematical operation — from zero-value substitution and centred log-ratio transformation to Aitchison distance computation — is fully described verbally and supported by relevant citations. No mathematical formulas or references to formula numbers appear anywhere in the revised text. These revisions comprehensively address all of the reviewer’s concerns regarding incomplete and structurally erroneous formulas.

Appendix : Bayesian Multiplicative Zero Replacement Code

library(readxl)

try <- read_excel("文件路径")

View(try)

cmultRepl(try, label = 0,method = c("GBM","SQ","BL","CZM","user"), output = c("prop","p-counts"),frac = 0.65, threshold = 0.5, adjust = TRUE, t = NULL, s = NULL,z.warning = 0.8, suppress.print = FALSE)

df<-cmultRepl(space, label = 0,method = c("GBM","SQ","BL","CZM","user"), output = c("prop","p-counts"),frac = 0.65, threshold = 0.5, adjust = TRUE, t = NULL, s = NULL,z.warning = 0.8, suppress.print = FALSE)

write.csv(df,"文件路径df.csv")

Comment 9

Reviewer’s Comment

The Dirichlet regression (lines 470 to 507) is clearly faulty – it ignores that Dirichlet models require conditional independence (see the Aitchison reference) and regression requires independent errors. Further, there are really only 2 data points as there are only 2 time periods, so I have no idea what analysis was done (and it is impossible to tell, given the dataset provided). The discussion linked to this analysis is hence clearly faulty as well.

Response

We highly appreciate the reviewer’s rigorous inspection of our Dirichlet regression analysis. We fully recognize that our two aggregated time periods fail to satisfy the core model assumptions of Dirichlet regression, including conditional independence and independent error terms. With merely two temporal groups, the sample size is insufficient to conduct valid significance testing via regression models. All interpretations derived from the Dirichlet regression outputs are therefore unreliable.

To resolve this fundamental methodological flaw, we have completely removed all Dirichlet regression content from the revised manuscript, including corresponding result tables, p-value outputs, and all associated discussion paragraphs. The Aitchison distance remains the sole quantitative metric for reporting cross-media compositional divergence, and all analytical results are presented as purely descriptive observations without any statistical significance claims.

This major revision eliminates invalid inferential analysis and aligns all empirical conclusions strictly with what our limited two-period dataset can reliably support, substantially improving the methodological rigor of this paper. We are grateful to the reviewer for detecting this critical error.

All revisions throughout the manuscript have been carefully double-checked to maintain consistent terminology, clear logical presentation and standardized statistical descriptions.

We firmly believe that the above revisions have fully addressed all your valuable suggestions and substantially strengthened the methodological rigor and reproducibility of this work. We are truly grateful for your precious time and insightful guidance that greatly helped us improve this paper.

Sincerely,

The Authors

Attachment Submitted filename: Response to Reviewer.docx

# Peer review: Decision Letter 5

**DOI:** 10.1371/journal.pone.0360383.r011

**Authors:** Sulemana Bankuoru Egala

23 Aug 2026

Dear Dr. Li,

plosone@plos.org. When you're ready to submit your revision, log on to https://www.editorialmanager.com/pone/ and select the 'Submissions Needing Revision' folder to locate your manuscript file.

- A letter that responds to each point raised by the academic editor and reviewer(s). You should upload this letter as a separate file labeled 'Response to Reviewers'.
- A marked-up copy of your manuscript that highlights changes made to the original version. You should upload this as a separate file labeled 'Revised Manuscript with Track Changes'.
- An unmarked version of your revised paper without tracked changes. You should upload this as a separate file labeled 'Manuscript'.

If you would like to make changes to your financial disclosure, please include your updated statement in your cover letter. Guidelines for resubmitting your figure files are available below the reviewer comments at the end of this letter.

If applicable, we recommend that you deposit your laboratory protocols in protocols.io to enhance the reproducibility of your results. Protocols.io assigns your protocol its own identifier (DOI) so that it can be cited independently in the future. For instructions see: https://journals.plos.org/plosone/s/submission-guidelines#loc-laboratory-protocols. Additionally, PLOS One offers an option for publishing peer-reviewed Lab Protocol articles, which describe protocols hosted on protocols.io. Read more information on sharing protocols at https://plos.org/protocols?utm_medium=editorial-email&utm_source=authorletters&utm_campaign=protocols.

As the corresponding author, your ORCID iD is verified in the submission system and will appear in the published article. PLOS supports the use of ORCID, and we encourage all coauthors to register for an ORCID iD and use it as well. Please encourage your coauthors to verify their ORCID iD within the submission system before final acceptance, as unverified ORCID iDs will not appear in the published article. *Only*  the individual author can complete the verification step; PLOS staff *cannot*  verify ORCID iDs on behalf of authors.

We look forward to receiving your revised manuscript.

Kind regards,

Sulemana Bankuoru Egala (PhD)

Academic Editor

PLOS One

Journal Requirements:

If the reviewer comments include a recommendation to cite specific previously published works, please review and evaluate these publications to determine whether they are relevant and should be cited. There is no requirement to cite these works unless the editor has indicated otherwise.

Please review your reference list to ensure that it is complete and correct. If you have cited papers that have been retracted, please include the rationale for doing so in the manuscript text, or remove these references and replace them with relevant current references. Any changes to the reference list should be mentioned in the rebuttal letter that accompanies your revised manuscript. If you need to cite a retracted article, indicate the article’s retracted status in the References list and also include a citation and full reference for the retraction notice.

[Note: HTML markup is below. Please do not edit.]

Reviewers' comments:

Reviewer's Responses to Questions

**Comments to the Author**

Reviewer #4: (No Response)

**********

2. Is the manuscript technically sound, and do the data support the conclusions?

Reviewer #4: Yes

**********

3. Has the statistical analysis been performed appropriately and rigorously?

Reviewer #4: Yes

**********

4. Have the authors made all data underlying the findings in their manuscript fully available?

The PLOS Data policy

Reviewer #4: No

**********

5. Is the manuscript presented in an intelligible fashion and written in standard English?

Reviewer #4: Yes

**********

Reviewer #4: Please see the attached file - the remaining issues are minor and should be addressable very fast by the authors

**********

what does this mean?). If published, this will include your full peer review and any attached files.

If you choose “no”, your identity will remain anonymous but your review may still be made public.

**Do you want your identity to be public for this peer review?**  For information about this choice, including consent withdrawal, please see our Privacy Policy

Reviewer #4: **Yes:**  Prof John Bacon-Shone

**********

[NOTE: If reviewer comments were submitted as an attachment file, they will be attached to this email and accessible via the submission site. Please log into your account, locate the manuscript record, and check for the action link "View Attachments". If this link does not appear, there are no attachment files.]

To ensure your figures meet our technical requirements, please review our figure guidelines: https://journals.plos.org/plosone/s/figures

You may also use PLOS’s free figure tool, NAAS, to help you prepare publication quality figures: https://journals.plos.org/plosone/s/figures#loc-tools-for-figure-preparation.

NAAS will assess whether your figures meet our technical requirements by comparing each figure against our figure specifications.

Attachment Submitted filename: Review of Measuring Destination Image Dissonance revised.pdf

# Author comment: Author response to Decision Letter 6

**DOI:** 10.1371/journal.pone.0360383.r012

4 Sep 2026

Point-by-Point Response to Reviewer Comments

Dear Prof. John Bacon‑Shone,

We are sincerely grateful for your thorough, constructive and insightful comments on our manuscript. Your careful feedback has greatly helped us improve the conceptual rigor, methodological clarity, and reproducibility of this work. We have addressed each of your concerns point‑by‑point below. All corresponding revisions have been implemented within the revised manuscript; modified text passages are quoted where applicable.

Comment R5-1. In the Abstract, the abbreviations PGC, TGC, and OGC should be spelled out in full at their first occurrence.

Response

We thank the reviewer for this reminder. In the revised Abstract, we have now spelled out all three abbreviations at their first occurrence: “Press Generated Content (PGC)”, “Tourist Generated Content (TGC)”, and “Official Generated Content (OGC)”. The revised sentence reads:

“This study examines Sanya’s tourism image disparities across cognitive, spatial, and affective dimensions in Press Generated Content (PGC), Tourist Generated Content (TGC), and Official Generated Content (OGC) (2013–2022), comparing pre-pandemic (2013–2019) and COVID-19 (2020–2022) periods.”

Comment R5-2. The statement in the previous response that "All data files are fully annotated with readme documents to ensure reproducibility of all keyword screening and CoDA procedures" is inaccurate. In reality, there is no annotated readme file, and the data labels are only in Chinese (without an English version), making it impossible to match them against the manuscript text. This must be corrected.

Response

We thank the reviewer for pointing out this inaccurate statement in our prior response. We sincerely apologize for this misrepresentation: no standalone readme document was prepared for the deposited dataset.

The original dataset and processing scripts originate from a Chinese‑language research workflow; hence variable labels and comments within the raw code are natively in Chinese. We have taken practical steps to improve cross‑referencing between dataset contents and the English manuscript text.

We have revised the deposited data files to add bilingual (Chinese‑English) labels for all core keyword entries and dimension categories (cognitive, spatial, affective) directly within the data tables. Each keyword is now paired with its English equivalent used in the manuscript, enabling reviewers and readers to match dataset entries with the descriptions presented in the article.

The complete executable processing R scripts have been uploaded to our public repository. Readers can trace all steps including keyword filtering, zero‑replacement, and Compositional Data Analysis procedures directly from the provided code.

Repository link:

https://github.com/x9w259pp86-stack/datesets-for-Plos-one/blob/main/datasets-updates2026-0901.zip

Comment R5-3. The statement in the previous response that "CA mainly identifies differences in raw count distributions based on chi-square distance, which cannot independently extract structural deviations of proportional compositions" is incorrect. No CA-related literature is cited, suggesting unfamiliarity with existing CA research. The reviewer recommends the open-access paper at [https://econ-papers.upf.edu/papers/1857.pdf], which demonstrates that CA can serve as an alternative to CoDA.

Response

We sincerely thank the reviewer for this valuable comment and for pointing out relevant alternative analytical workflows based on correspondence analysis.

Unfortunately, the direct working‑paper link provided by the reviewer was inaccessible. Following the reviewer’s note that this work has been released in a revised peer‑reviewed journal version, we have identified the formally published article by Greenacre (2024). This reference has been added to the reference list of our revised manuscript.

We recognise that our original statement was oversimplified and overly absolute, dismissing the analytical potential of CA‑based pipelines for compositional‑type research questions. We have revised Section 3.3 of the manuscript to acknowledge that CA‑based approaches can represent feasible alternatives for compositional‑like frequency‑based datasets.

Our choice to adopt the CoDA framework with centered log‑ratio transformation and Aitchison distance is driven by our specific research goal: measuring compositional structural divergence under the strict closed constant‑sum constraint for our keyword proportions. This selection does not imply that CA‑based methods are inherently invalid.

The revised text in Section 3.3 reads:

Correspondence Analysis (CA) operates on raw count contingency tables using chi-square distance. Published literature has demonstrated that CA-based workflows can serve as feasible alternatives for compositional-like frequency-based datasets [53]. Nevertheless, standard CA does not explicitly address the closed constant-sum constraint central to Aitchison-style compositional-data analysis. Given our core objective of quantifying structural divergence within closed proportional keyword compositions, we adopt the centered log-ratio transformation and Aitchison distance within the CoDA framework in the present study.

Comment R5-4. The claim that "70% cumulative word frequency coverage acts as the critical cutoff separating informative high- and medium-frequency terms from trivial long-tail low-frequency words" is incorrect. The appropriate threshold is dataset-dependent, and the best way to justify a threshold is to present cumulative coverage curves. The authors should add a figure demonstrating that the threshold is reasonable for these three datasets.

Response

We thank the reviewer for pointing out this important conceptual caveat. We fully agree that a cumulative word‑frequency coverage value of 70 % should not be treated as a universal critical cutoff, since suitable thresholds for keyword selection are inherently dataset‑dependent. Accordingly, we have removed this absolute claim from the revised manuscript. We now interpret the 70 %‑80 % range reported in Liu et al. (2016) only as a heuristic empirical reference for co‑word analysis, rather than a definitive fixed criterion.

To empirically justify our keyword‑selection procedure, we have added a new figure (Figure 2) showing cumulative word‑frequency‑coverage curves across all six dataset subsets (PGC, TGC, OGC for both the pre‑pandemic 2013‑2019 phase and the pandemic‑impacted 2020‑2022 phase. Curves are plotted based on each subset’s extracted top‑100 candidate keywords. It should be noted that our final 50 keywords used for compositional‑data analysis were selected from a merged global candidate pool aggregated across three media sources, sorted by summed absolute frequency across PGC, TGC and OGC, rather than directly truncating rank 1‑50 from each individual dataset subset, a horizontal dashed line indicates the heuristic 70 % reference level from prior literature. The curves visually illustrate the typical long‑tail distribution of word frequencies within each media‑period subset.

We would like to further clarify the rationality of selecting 50 keywords, and distinguish two different statistical scopes to avoid potential confusion.

First, Figure 2 visualizes cumulative word‑frequency coverage within each individual media‑period subset’s top‑100 candidate pool, which demonstrates word‑frequency long‑tail characteristics. In contrast, the reported coverage values of 78.22 % (Phase 1) and 79.23 % (Phase 2) are calculated on the merged, deduplicated global candidate pool aggregated from PGC, TGC and OGC. These two metrics are computed over different keyword populations and are therefore not expected to match numerically.

Second, we acknowledge that if we expanded the per‑media extraction threshold to top‑200 or top‑300 words, the size of the merged global candidate pool would increase, and the cumulative coverage of the global top‑50 keywords would indeed be diluted. This is exactly why we adopted top‑100 as the per‑media extraction boundary. Extracting substantially more keywords per source would introduce numerous sporadic low‑frequency noise terms with marginal semantic contribution to core destination‑image features. These extra terms would lower the global coverage metric without adding meaningful analytical value.

Third, the 70 %‑80 % heuristic reference range proposed by Liu et al. (2016) operates at this merged‑global‑candidate‑pool level. Our two observed values (78.22 % and 79.23 %) fall well within this empirical interval.

Beyond frequency‑based coverage, semantic interpretability forms another essential constraint for our study. Our analytical framework requires manual classification of each keyword into cognitive, spatial, or affective dimensions. A substantially larger keyword set would bring excessive low‑signal sporadic terms and impair the reliability of dimensional categorization. Conversely, retaining too few keywords would omit essential destination‑image dimensions. Therefore, selecting 50 keywords represents a practical balance between adequate frequency coverage and manageable semantic‑dimensional interpretability for our core goal of measuring structural destination‑image dissonance. Our analysis targets dominant semantic elements of destination images and does not aim to exhaust all infrequent long‑tail vocabulary.

The revised text within Section 3.3 “Construction of a unified analytical matrix” reads:

Liu et al. (2016) reported a heuristic empirical reference range of 70%‑80% cumulative word‑frequency coverage for keyword filtering in co‑word analysis. Importantly, such coverage values are dataset‑specific and should not be treated as a universal fixed cutoff. To visually justify our keyword‑selection procedure, we provide cumulative word‑frequency‑coverage curves for each of the six dataset subsets (PGC, TGC, OGC across both time periods) based on their respective top‑100 candidate keywords (Figure 2). The curves illustrate how word frequency accumulates within each individual media‑period subset. Our selected top‑50 global keywords fall within a stable high‑coverage region and adequately represent dominant semantic features of Sanya‑tourism textual data, supporting the suitability of this keyword subset for subsequent compositional‑data analysis. In addition to frequency‑coverage metrics, semantic interpretability for cognitive‑spatial‑affective classification was also considered when determining the size of the final keyword subset. An excessively large keyword pool would introduce many low‑signal sporadic terms and compromise the reliability of dimensional classification.

We once again thank Prof. Bacon‑Shone for your rigorous evaluation and valuable suggestions, which have substantially strengthened this manuscript. We hope the revised version is now suitable for further consideration for publication.

Sincerely,

The Authors

Attachment Submitted filename: Response_to_Reviewers_auresp_6.docx

# Peer review: Decision Letter 6

**DOI:** 10.1371/journal.pone.0360383.r013

**Authors:** Sulemana Bankuoru Egala

28 Sep 2026

Measuring Destination Image Dissonance: A Compositional Data Analysis of Cross-Media Representations in Sanya's Tourism

PONE-D-25-45869R6

Dear Dr. Xueli Li,

We’re pleased to inform you that your manuscript has been judged scientifically suitable for publication and will be formally accepted for publication once it meets all outstanding technical requirements.

Within one week, you’ll receive an e-mail detailing the required amendments. When these have been addressed, you’ll receive a formal acceptance letter and your manuscript will be scheduled for publication.

An invoice will be generated when your article is formally accepted. Please note, if your institution has a publishing partnership with PLOS and your article meets the relevant criteria, all or part of your publication costs will be covered. Please make sure your user information is up-to-date by logging into Editorial Manager at Editorial Manager® and clicking the ‘Update My Information' link at the top of the page. For questions related to billing, please contact billing support.

If your institution or institutions have a press office, please notify them about your upcoming paper to help maximize its impact. If they’ll be preparing press materials, please inform our press team as soon as possible -- no later than 48 hours after receiving the formal acceptance. Your manuscript will remain under strict press embargo until 2 pm Eastern Time on the date of publication. For more information, please contact onepress@plos.org.

Kind regards,

Sulemana Bankuoru Egala (PhD)

Academic Editor

PLOS One

Additional Editor Comments (optional):

Reviewers' comments:

Reviewer's Responses to Questions

**Comments to the Author**

Reviewer #1: All comments have been addressed

Reviewer #3: All comments have been addressed

Reviewer #4: All comments have been addressed

**********

2. Is the manuscript technically sound, and do the data support the conclusions?

Reviewer #1: Yes

Reviewer #3: Yes

Reviewer #4: Yes

**********

3. Has the statistical analysis been performed appropriately and rigorously?

Reviewer #1: Yes

Reviewer #3: Yes

Reviewer #4: Yes

**********

4. Have the authors made all data underlying the findings in their manuscript fully available?

The PLOS Data policy

Reviewer #1: Yes

Reviewer #3: Yes

Reviewer #4: Yes

**********

5. Is the manuscript presented in an intelligible fashion and written in standard English?

Reviewer #1: Yes

Reviewer #3: Yes

Reviewer #4: Yes

**********

Reviewer #1: This paper addresses interesting and relevant questions regarding destination image dissonance between Press-Generated Content (PGC), Tourism-Generated Content (TGC), and Officially Generated Content (OGC), using Compositional Data Analysis (CoDA). The use of centralized log ratio (CLR) and Aitchison distance transformations is conceptually appropriate for comparing proportional keyword compositions, and this paper provides a useful effort to move destination image analysis beyond simple frequency comparisons. The manuscript has also made several positive methodological improvements, including clarification of the reasons for CoDA, documentation of zero-value treatment, inter-coder reliability assessments, and explicit recognition that the two-period design does not allow for the causal attribution of observed changes to COVID-19.

Reviewer #3: Congratulations on a successful revision! The authors have adequately addressed the review comments. I don't have further comments for improvements.

Reviewer #4: I am happy with all the responses to my previous review, with one exception. Because Correspondence Analysis conditions on row and column totals, it still applies implicit unit sums, although I agree that the distance metric from CoDA may be more directly applied in this case.

**********

what does this mean?). If published, this will include your full peer review and any attached files.

If you choose “no”, your identity will remain anonymous but your review may still be made public.

**Do you want your identity to be public for this peer review?**  For information about this choice, including consent withdrawal, please see our Privacy Policy

Reviewer #1: No

Reviewer #3: **Yes:**  Tingting Zhang

Reviewer #4: **Yes:**  John Bacon-Shone

**********

# Editor report: Acceptance letter

**DOI:** 10.1371/journal.pone.0360383.r014

**Authors:** Sulemana Bankuoru Egala

PONE-D-25-45869R6

PLOS One

Dear Dr. Li,

I'm pleased to inform you that your manuscript has been deemed suitable for publication in PLOS One. Congratulations! Your manuscript is now being handed over to our production team.

At this stage, our production department will prepare your paper for publication. This includes ensuring the following:

* All references, tables, and figures are properly cited

* All relevant supporting information is included in the manuscript submission,

* There are no issues that prevent the paper from being properly typeset

You will receive further instructions from the production team, including instructions on how to review your proof when it is ready. Please keep in mind that we are working through a large volume of accepted articles, so please give us a few days to review your paper and let you know the next and final steps.

Lastly, if your institution or institutions have a press office, please let them know about your upcoming paper now to help maximize its impact. If they'll be preparing press materials, please inform our press team within the next 48 hours. Your manuscript will remain under strict press embargo until 2 pm Eastern Time on the date of publication. For more information, please contact onepress@plos.org.

You will receive an invoice from PLOS for your publication fee after your manuscript has reached the completed accept phase. If you receive an email requesting payment before acceptance or for any other service, this may be a phishing scheme. Learn how to identify phishing emails and protect your accounts at https://explore.plos.org/phishing.

If we can help with anything else, please email us at customercare@plos.org.

Thank you for submitting your work to PLOS One and supporting open access.

Kind regards,

PLOS One Editorial Office Staff

on behalf of

Prof. Sulemana Bankuoru Egala

Academic Editor

PLOS One
